# Supplementary material for: Quantitative Succinyl-Proteome Profiling of Turnip (Brassica rapa var. rapa) in Response to Cadmium Stress
Source: Cells. 2022 Jun 17;11(12):1947. doi: 10.3390/cells11121947 (PMC9221971; doi:10.3390/cells11121947)
Supplement: Supplementary file 1 [file cells-11-01947-s001.zip › Supplementary Protein Sequences.pdf]

Sequences of the 256 succinylated proteins

>Gene0014310.1

MGLVSKTCCPHQTLHSLDYLHARSLAFSLDLIAMADNKKIKIGINGFGRIGRLVARVIL

QRNDVELVAVNDPFITTEYMTYMFKYDSVHGQWKHNELKIKDEKTLLFGEKPVTVF

GIRNPEEIPWGEAGADFVVESTGVFTDKDKAAAHLKGGAKKVVISAPSKDAPMFVV

GVNEHEYKSDLNIVSNASCTTNCLAPLAKVINDRFGIVEGLMTTVHSITATQKTVDGP

SMKDWRGGRAASFNIIPSSTGAAKAVGKVLPQLNGKLTGMSFRVPTVDVSVVDLTVR

LEKAATYDEIKKAIKEESEGKLKGILGYTEDDVVSTDFVGDNRSSIFDAKAGIALSDNF

VKLVSWYDNEWGYSTRVVDLIIHMSKA.

>Gene0001310.1

MANPKVFFDILIGKMKAGRVVMELFADVTPRTADNFRALCTGEKGIGQAGKALHYK

GSAFHRIIPGFMCQGGDFTRGNGTGGESIYGAKFQDENFKLKHTGPGILSMANSGPNT

NGSQFFICTDKTAWLDGKHVVFGKVVDGYNVVKAMEKVGSERGATSEPVVIEDCGE

LKNETSEEVSNKE.

>Gene0314370.1

MASVAASPAFSLLKSTGGAIASSAATRARASLLPITPSSKSISPRPLGFSAVLDSHRFSLH

VASKVHSVRGRGSRGVVSMAKKSVGDLTSADLKGKKVFVRADLNVPLDDNQTITDD

TRIRAAIPTIKYLIENGAKVILSTHLGRPKGVTPKFSLAPLVPRLSELLGIEVKKADDCI

GPEVESLVASLPEGGVLLLENVRFYKEEEKNDPEFAKKLASLADLYVNDAFGTAHRA

HASTEGVTKFLKPSVAGFLLQKELDYLVGAVSNPKRPFAAIVGGSKVSSKIGVIESLLE

KCDILLLGGGMIFTFYKAQGLSVGSSLVEEDKLELATSLLAKAKAKGVSLLLPTDVVV

ADKFAPDANSKVVPASGIEDGWMGLDIGPDSIKTFNEALDTTQTVIWNGPMGVFEME

KFAAGTEAVANKLAELSEKGVTTIIGGGDSVAAVEKVGVAGVMSHISTGGGASLELLE

GKVLPGVIALDEAITVTV.

>Gene0039030.1

MVEQTQNKLMRTGVVSHDIHGYTSSFQRRATYGNYTNAAFQYPLAATSRIVATTTTT

SPVFVQAPSEKGFSSFAIDFLMGGVSAAVSKTAAAPIERVKLLIQNQDEMLKAGRLSEP

YKGIGDCFGRTIKDEGFGSLWRGNTANVIRYFPTQALNFAFKDYFKRLFNFKKDRDG

YWKWFAGNLGSGGAAGASSLLFVYSLDYARTRLANDSKAAKKGGERQFNGLVDVY

KKTLKSDGIAGLYRGFNISCVGIIVYRGLYFGLYDSLKPLLPADLQDSFFASFALGWLIT
NGAGLASYPIDTVRRRMMMTSGEAVKYKSSMDAFQQILKKEGPKSLFKGAGANILR
AIAGAGVLSGYDKLQLLLLGKKYGSGSG.

>Gene0003390.1

MNKQRHPSVFQKIRGQTSLISTLSPTVQPRNHSISGAFVNGGLQSLLQSTSHGTSLIPR
GSLPVLAQAPTEKSSTGFLIDFLMGGVSAAVSKTAAAPIERVKLLIQNQDEMLRAGRL
SEPYKGITDCFTRTVKDEGVLSLWRGNTANVLRYFPTQALNFAFKDYFKRLFNFKKD
KDGYWKWFAGNLASGGAAGASSLLFVYSLDYARTRLANDAKAAKKGGQRQFNGIV
DVYKKTVASDGVVGLYRGFNISCVGIIVYRGLYFGLYDSLKPVVLVDGLEDNFLASFL
LGWGITIGAGLASYPIDTVRRRMMMTSGEAVKYTSSLQAFNQIVKKEGAKSLFKGAG
ANILRAVAGAGVLAGYDKLQLLVFGKKYGSGSG.

>Gene0078230.1

YGNLPSQSQLADWEFTISQHSAVPQGVLEIIQSMPHDAHPMGVLVSAMSALSIFHPDA
NPALSGQDIYKSKQIRDKQIVRILGKAPTIAAAAYLRMAGRPPVLPSGNLSYAENFLY
MLDSMGNRSYKPNPRLARVLDILFILHAEHEMNCSTAAARHLASSGVDVYTAVAGAV
GALYGPLHGGANEAVLKMLAEIGSVENIPEFIEGVKNRKRKMSGFGHRVYKNYDPRA
KVIKKLADEVFSIVGRDPLIEVAVALEKAALSDEYFVKRKLYPNVDFYSGLIYRAMGF
PPEFFTVLFAVPRMAGYLSHWRESLDDPDTKIMRPQQAYTGVWLRHYEPVRQRTLSS
DSDKMGQVSISNASRRRLSGSAL.

>Gene0144720.1

MALSRLSLRSTTFLKPSSLRRHVTTDTTPITIETAAPFTSHQCDPPSRSVETSSAEILSFF
RDMARMRRMEIAADSLYKSKLIRGFCHLYDGQEALAVGMEAAITKKDAIITSYRDHC
TFLGRGGELVDAFSELMGRMRGCSNGKGGSMHFYKKDACFYGGHGIVGAQIPLGCG
LAFAQKYSKEENVSFVLYGDGAANQGQLFEALNIAALWDLPAILVCENNHYGMGTAT
WRSAKSPAYFKRGDYVPGLKVDGMDVLAVKQACKFAKEHALKNGPIILEMDTYRYH
GHSMSDPGSTYRTRDEVSGVRQVRDPIERVRKLLLSHDIATEKELKDMEKEVRKEVD
DAVAQAKESPVPEASELFTNMYVKDCGVESFGADRKELKVTLA.

>Gene0005420.1

MAQILAASPTCQMKLTKPSPIASSKLWSSVMLKQKKQNSSKLRSFKVMALQSDNSTI
NRVESLLNLDTKPFTDRIIAEYIWIGGSGIDLRSKSRTLEKPVEDPSELPKWNYDGSST

GQAPGEDSEVILYPQAIFRDPFRGGDNILVICDTYTPAGEPIPTNKRARAAEIFSNKKVN
EEIPWFGIEQEYTLLQPNVNWPLGWPVGAYPGPQGPYYCGVGAEKSWGRDISDAHY
KACLYAGINISGTNGEVMPGQWEFQVGPSVGIEAGDHVWCARYLLERITEQAGVVLT
LDPKPIEGDWNGAGCHTNYSTKSMREEGGFDVIKKAILNLSLRHMEHISAYGEGNER
RLTGKHETASIDQFSWGVANRGCSIRVGRDTEKKGKGYLEDRRPASNMDPYIVTSLLA
ETTLLWEPTLEAEALAAQKLSLKV.

>Gene0005530.1

MASEDVKRREGVNKAPSNAVIGICKSLIAGGVAGGVSRTAVAPLERLKILLQVQNPHSI
KYNGTVQGLKYIWRTEGFRGLFKGNGANCARIVPNSAVKFFSYEQASKGILYLYRQQ
TGNDDAQLTPLLRLGAGACAGIIAMSATYPMDMVRGRLTVQTDKSPYQYRGMVHAL
STVLRQEGPRALYRGWLPSVIGVVPYVGLNFAVYESLKDWLVKSKPFGIIDNNTSELT
VTTRLACGAIAGTMGQTVAYPLDVVRRRMQMVGWKDASSVITGDGRGKAPIEYSG
MVDAFRKTVRHEGLGALYKGLVPNSVKVVPSIAIAFVTYEKVKDILGVEFRISD.

>Gene0277440.1

MATQISKKRKFVADGVFYAELNEVLTRELAEDGYSGVEVRVTPMRTEIIIRATRTQNVL
GEKGRRIRELTSLVQKRFRFPQDSVELYAEKVANRGLCAIAQAESLRYKLLGGLAVRR
ACYGVLRFVMESGAKGCEVIVSGKLRAARAKSMKFKDGYMVSSGQPTKDYIDSAVR
HVLLRQGVLGIKVKVMLDWDPKGINGPKTPLPDVVIIHAPKEEDVSSAPAQVAAPAA
LLPEAPLTAVDYPEMIPVA.

>Gene0008940.1

MVLGAAARVAIVGCRRLVCSSSHASPLLVSSQCRQMSMDAQSVSEKLRSSGLLRTQG
LIGGKWIDSYDKTTIKVNNPATGEIVADVACMGVKETNDAIASSYEAFQSWSTRTAGE
RSRVLRRWFDLLVAHKEELGQLITLEQGKPLKEAIGEVAYGASFIEYYAEEAKRVYGDI
IPPNASDRRLLVLKQPVGVVGAITPWNFPLAMITRKVGPALASGCTVVVKPSELTPLTA
LAAAELALQAGVPPGALNVVMGNAPEIGDALLASPQVRKITFTGSTAVGKKLMAAA
APTVKKVSLELGGNAPSIIFDDADLDVAVKGTLAAKFRNSGQTCVCANRVLVQDGIY
DKFAEAFSEAVQKLEVGDGFKEGTTQGPLINDAAIQKVESFVQDAVSKGAKILLGGK
KHSLGMTFYEPTVIRDVTSNMIMSKEEIFGPVAPLIRFKTEEDAIRIANDTIAGLAAYIF
TNSVQRSWRVSEALEYGLVGVNEGIISTEVAPFGGVKQSGLGREGSKYGMDEYHEIK
YICMGDMNRQ.

>Gene0009350.1

MVKSEVKTEKLDDDITIKDNKHRRRVVSSAEAIDRKFKRTVLCVAKPSYLLSLLERSS

TRCNYLKRLPKILSELLRQRNWREASGVLSVLMQGTMRDGSPSMNRLKYEAQIQIVS

HLQPDKNNVEEIERIYDTWIGKIGKQHKEERLLVWFEQICHLLEHGMEKEAEYAAIG

MMRSRDLGNLPRTNLYIGITCYRLWCRKYSEELEPKDADCSDSISNMSQSGSGVMAE

CSPRNESVYSVESSGSVRNVSEASVGNCEVNSDASTRDSGSVVEVKVKLENVKVEES

CQHFTEPPRIYASSEENEEPLRDGVSFDPALVQILGKMDPWLLPFKPPEDPDCHGKIVN

DSFYKDAVSYLRLTMQSPRYVSLAALHPLVQLLLIGGRVDEAMKLVEEMCNKVHDIK

PFRIRAAMKEKFHNNSDELAKCYEDVLKIDPSCVTTLKKLIEMSKEDGYSRESLIEMI

ALHVEASFPEPQIWKEFAEMLILFFENVDEDRMSVCLNRDGEEGCQQTYSVRYNRTP

RMFTGTSWTRRAKWWLNRHFSPEILETEMKKLEAEQINGDLEMLRLMSFKAACASR

LYGPEFGYVTTVYGLLESCRNNIQNMCMLENDSNNIGMLENGRNSSELFNFVRRHRQ

NWNRIYNLE.

>Gene0123310.1

MAEEDLECGRWCFVELLRTVKKSSRQVIEKYYSRMTLDFHTNKKILEEVAIIPSKRLR

NKIAGFSTHLMKRIQKGPVRGISLKLQEEERERRMDFVPDESAIKTDVIKVDKES.

>Gene0011240.1

METSVTCYSRGIILPSVSSQRSSTLVSPPSSFSASSSFKRLKSSSIFGESLRVAPRSQLKAT

KAKNNGGLTVTKCEIGQSLEEFLREATPDKGLRTLLMCMGEALRTIAFKVRTASCGGT

ACVNSFGDEQLAVDMLADKLLFEALQYSHVCKYACSEEVPELQDMGGPVEGGFSVA

FDPLDGSSIVDTNFTVGTIFGVWPGDKLTGVTGGDQVAAAMGIYGPRTTYVLAVKGF

PGTHEFLLLDEGKWQHVKETTEINEGKMFSPGNLRATFDNSEYSKLIDYYVKEKYTL

RYTGGMVPDVNQIIVKEKGIFTNVTSPTAKAKLRLLFEVAPLGLLIENAGGFSSDGYKS

VLDKTIVNLDDRTQVAYGSKNEIIRFEETLYGTSRLKNVPIGANA.

>Gene0011600.1

MAAAVSTVGAINRAPLSLNGSGAGAASVPATTFLGKKVVTASRFTQSNNKKSNGSFK

VVAVKEDKQTDGDRWRGLAYDTSDDQQDITRGKGMVDSVFQAPMGTGTHNAVLSS

YEYISQGLKQYNLDNMMDGLYIAPAFMDKLVVHITKNFLTLPNIKVPLILGIWGGKGQ

GKSFQCELVMAKMGINPIMMSAGELESGNAGEPAKLIRQRYREAADMIKKGKMCCL

FINDLDAGAGRMGGTTQYTVNNQMVNATLMNIADNPTNVQLPGMYNKEENARVPII

VTGNDFSTLYAPLIRDGRMEKFYWAPTREDRIGVCKGIFRTDNVKDEDIVTLVDQFPG

QSIDFFGALRARVYDDEVRKFVEGLGVEKIGKRLVNSREGPPVFEQPAMTLEKLMEY

GNMLVMEQENVKRVQLADQYLNEAALGDANADAIGRGTFYGTRPEPSN.

>Gene0107960.1

MAAAVSTVGAINRAPLSLNGSGAGAASVPATTFLGKKVVTASRFAQNNKKSNGSFKV

VAVKEDKQTDGDRWRGLAYDMSDDQQDITRGKGMVDSVFQAPMGTGTHNAVLSSY

EYISQGLKQYNLDNMMDGLYIAPAFMDKLVVHITKNFLTLPNIKVPLILGVWGGKGQ

GKSFQCELVMAKMGINPIMMSAGELESGNAGEPAKLIRQRYREAADLIKKGKMCCLF

INDLDAGAGRMGGTTQYTVNNQMVNATLMNIADNPTNVQLPGMYNKEDNARVPIIV

TGNDFSTLYAPLIRDGRMEKFYWAPTREDRIGVCKGIFRTDKIKDEDIVTLVDQFPGQS

IDFFGALRARVYDDEVRKFVEGLGVEKISKRLVNSREGPPVFEQPEMTLEKLMEYGN

MLVMEQENVKRVQLADQYLNEAALGDANADAIGRGTFFGQTTGQA.

>Gene0013920.1

MALRMWASSTANALKLSSSASRSHLLPAFSISRCFSSVLEGLKYANSHEWVKHEGSVA

TIGISDHAQDHLGEVVFVELPEEKSSVTKEKNFGAVESVKATSEIISPISGEVIEVNTKL

ADSPGLINSSPYEDGWMIKVKPSNPTELESLMGPKEYTKFCEEEDAAH.

>Gene0453640.1

MAAAVEIDAEIQQQLTNEVKLFNRWSFDDVSVTDISLVDYIGVQPAKHATFVPHTAGR

YSVKRFRKAQCPIVERLTNSLMMHGRNNGKKLMAVRIIKHAMEIIHLLTDLNPIQVIID

AIVNSGPREDATRIGSAGVVRRQAVDISPLRRVNQAIFLLTTGAREAAFRNIKTIAECLA

DELINAAKGSSNSYAIKKKDEIERVAKANR.

>Gene0393450.1

MSGKGEGPAIGIDLGTTYSCVGVWQHDRVEIIANDQGNRTTPSYVAFTDSERLIGDAA

KNQVAMNPINTVFDAKRLIGRRFSDSSVQSDMKLWPFKIIAGPAEKPMIVVNYKGEEK

QFAAEEISSMVLIKMREIAEAYLGVTIKNAVVTVPAYFNDSQRQATKDAGVIAGLNVM

RIINEPTAAAIAYGLDKKATSVGEKNVLIFDLGGGTFDVSLLTIEEGIFEVKATAGDTHL

GGEDFDNRMVNHFVQEFKRKSKKDITGNPRALRRFEELNMDLFRKCMEPVEKCLRD

AKMDKSTVHDVVLVGGSTRIPKVQQLLQDFFNGKELCKSINPDEAVAYGAAVQGAIL

SGEGNEKVQDLLLLDVTPLSLGLETAGGVMTTLIARNTTIPTKKEQVFSTYSDNQPGV

LIQVFEGERARTKDNNLLGKFELSGIPPAPRGVPQITVCFDIDANGILNVSAEDKTTGQ

KNKITITNDKGRLSKDDIEKMVQEAEKYKSEDEEHKKKVEAKNALENYAYNMRNTI
QDEKIGEKLPAADKKKIEESIEQAIQWLENNQLGEADEFEDKMKELESICNPIIAKMY
QGAGGEAAGMDDDAPPASGGAGPKIEEVD.

>Gene0016330.1

MIFDSPNTSLLFFLYSKNFATKLNIFFIMRINPTTSDPAVSIREKNNLGRIAQIIGPVLDVA
FPPGKMPNIYNALVVKGRDTLGQEINVTCEVQQLLGNDRVRPRGMDVVDMGNPLSV
PIGGVTLGRIFNVLEEPVDNLGPIDTLTTSPIHKSAPAFIDLDTTLSIFETGIKVVDLLAP
YRRGGKIGLFGGAGVGKTVLIMELINNIAKAHGGVSVFGGVGERTREGNDLYMEMK
ESGVINELNLADSKVALVYGQMNEPPGARMRVGLTALTMAEYFRDVNEQDVLLFIDN
IFRFVQAGSEVSALLGRMPSAVGYQPTLSTEMGSLQERITSTKKGSITSIQAVYVPADD
LTDPAPATTFAHLDATTVLSRGLAAKGIYPAVDPLDSTSTMLQPRIVGEEHYETAQQVK
QTLQRYKELQDIIAILGLDELSEEDRLTVARARKIERFLSQPFFVAEVFTGSPGKYVGLA
ETIRGFNLILSGEFDSLPEQAFYLVGNIDEATAKATNLEMEKVKEIILSTNSGQIGVLPN
HAPIATAVDIEKNSDIDPQEAQQTLEIAEANLRKAEGKRQTIEANLALRRARTRVEALN
TI.

>Gene0078550.1

METPKTSSLIPSFLYSSSPRSILEKSPSPAARSSPAATMVSRKSFLIASPTEPGKGIEMYSP
AFYAACTFGGVLSCGLTHMTVTPLDLVKCNMQIDPAKYKSISSGFGILLKEQGVKGFF
RGWVPTLLGYSAQGACKFGFYEFFKKYYSDLAGPEFAAKYKTLIYLAGSASAEVIAD
VALCPFEAVKVRVQTQPGFARGMSDGFPKFVKSEGYGGLYKGLAPLWGRQIPYTMM
KFASFETIVEMIYKYAIPNPKHECSKGLQLGVSFAGGYVAGVFCAIVSHPADNLVSFLN
NAKGATVGDAVKKIGMVGLFTRGLPLRIVMIGTLTGAQWGLYDAFKVFVGLPTTGG
VTPAPVIAAAEA.

>Gene0017410.1

MEKNIKFPVVDLSKLIGEERDQTMALINDACENWGFFEIVNHGLPHDLMDNVEKMT
KEHYKISMEQKFNDMLKSKGLENLEREVEDVDWESTFYLRHLPQSNLYDIPDMSDEY
RTAMKDFGKRLENLAEDLLDLLCENLGLEKGYLKKVFHGTKGPTFGTKVSNYPACP
KPEMIKGLRAHTDAGGIILLFQDDKVSGLQLLKDGDWIDVPPLNHSIVINLGDQLEVI
TNGRYKSVMHRVVTQKEGNRMSIASFYNPGSDAEISPASSLACKETEYPSFVFDDYM
KLYAGVKFQPKEPRFEAMKNANAVTELNPTAAVETF.

>Gene0017600.1

MATFLTPLVSIKPTVFSFPSQSVTSPHRQTISRVRFIPHAVETEEKPASDPNAESSRRVYI
GNIPRTVDNEQLSKLVEEHGAAENVQVGFATMKSVEDANAVIDKLNGTTIEGREVKV
NITEKPIASSSSPDLSLLQSEDSAFVDSPYKVYVGNLAKTVTKQMLENLFSEKGKVVS
AKVSRVPGTSKSSGFGFVTFSTEEDVEAAILALNNSVIVVEGQKIRVNKA.

>Gene0475550.1

MEKGKGRKEEIVTREYTINLHRRLHSCAIKEIRKFVLKAMGTKDVRVDVKLNKQIWS
RGIHGPPRRVRVCVARKRNDDEDAKEEFYSLVTVAEIPAEGLSSLGTKVIDEDE.

>Gene0329070.1

MASSTAQIHVLGGIGFAASSKRNLNAKANLKPRSAFFGTRAGPFSAQTSAFLKMNTR
KGSRYAVGPVRVANEKVVGIDLGTTNSAVAAMEGGKPTIVTNAEGQRTTPSVVAYTKS
GDRLVGQIAKRQAVVNPENTFFSVKRFIGRRMNEVDEEAKQVSYRVVKDDNGNVKL
ECPAIGKQFAAEEISAQVLRKLVDDASRFLNEKVTKAVVTVPAYFNDSQRTATKDAGR
IAGLEVLRIINEPTAASLAYGFERKSNETILVFDLGGGTFDVSVLEVGDGVFEVLSTSG
DTHLGGDDFDKVNEGIDLLKDKQALQRLTEAAEKAKIELSSLTQTNMSLPFITATADG
PKHIETTLTRAKFEELCSDLLDRCKTPVENSLRDAKLSFKDIDEVILVGGSTRIPAVQEV
VRKLTGKEPNVTVNPDEVVALGAAVQAGVLAGDVSDIVLLDVTPLSIGLETLGGVMT
KIIPRNSTLPTSKSEVFSTAADGQTSVEINVLQGEREFVRDNKSLGSFRLDGIPPAPRGV
PQIEVKFDIDANGILSVSASDKGTGKKQDITITGASTLPKDEVEQMVQEAERFAKDDK
EKREAIDTKNQADSVVYQTEKQLKELGEKIPGEVKEKVEAKLQELKDKLASGTTQEI
KDTMAALNQEVMQIGQSMYNQPGAGAGAGAAAAGPSPGGEDASADSASKGGDDVI
DADFTDSK.

>Gene0443480.1

MASSAAQIHVLGGIGFATTSSKRNLNSKTTSIPRSAFFGTRTGPFSTPNSAFLRINTRNS
PGASRYAAGPVRVVNEKVVGIDLGTTNSAVAAMEGGKPTIVTNAEGQRTTPSVVAYT
KSGDRLVGQIAKRQAVVNPENTFFSVKRFIGRRMNEVDEEAKQVSYRVVKDDNGNV
KLECPAIGKQFAAEEISAQVLRKLVDDASRFLNEKVTKAVVTVPAYFNDSQRTATKDA
GRIAGLEVLRIINEPTAASLAYGFERKSNETILVFDLGGGTFDVSVLEVGDGVFEVLST
SGDTHLGGDDFDKRVVDWLALNFKKDEGIDLLKDKQALQRLTEAAEKAKIELSSLTQ
TNMSLPFITATADGPKHIETTLTRAKFEELCSDLLDRCKTPVENSLRDAKLSFKDIDEVI

LVGGSTRIPAVQEVVRKLTGKEPNVTVNPDEVVALGAAVQAGVLAGDVSDIVLLDVT
PLSIGLETLGGVMTKIIPRNSTLPTSKSEVFSTAADGQTSVEINVLQGEREFVRDNKSL
GSFRLDGIPPAPRGVPQIEVKFDIDANGILSVSASDKGTGKKQDITITGASTLPKDEVEQ
MVQEAERFAKDDKEKRDAIDTKNQADSVVYQTEKQLKELGEKIPGEVKEKVEAKLQ
ELKDKIGNGSTQEIKDTMAALNQEVMQIGQSMYNQPGAGAGAGAGAGAGAGSSPG
GEGDSSSSKGGDDVIDADFTDSN.

>Gene0019260.1

MASSAAQIHVLGGMGFTTSSSKRNLNGKSSFMPRSAFFGARNGPFSTSTSSFLRMRSR
NGGSSRYAVGPVRVVNEKVVGIDLGTTNSAVAAMEGGKPTIVTNAEGQRTTPSVVAY
TKSGDRLVGQIAKRQAVVNPENTFFSVKRFIGRRMNEVAEESKQVSYRVVEDENGNV
KLECPAIGKQFAAEEISAQVLRKLVDDASRFLNDKVTKAVITVPAYFNDSQRTATKDA
GRIAGLDVLRIINEPTAASLAYGFERKSNETILVFDLGGGTFDVSVLEVGDGVFEVLST
SGDTHLGGDDFDKRVVDWLASNFKKDEGIDLLKDKQALQRLTEAAEKAKIELSSLTQ
TNMSLPFITATADGPKHIETTLTRAKFEELCSDLLDRCKTPVENSLRDAKLSFSDIDEVI
LVGGSTRIPAVQEVVRKLTGKEPNVTVNPDEVVALGAAVQAGVLAGDVSDIVLLDVT
PLSIGLETLGGVMTKIIPRNTTLPTSKSEVFSTAADGQTSVEINVLQGEREFVKDNKSL
GSFRLDGIPPAPRGVPQIEVKFDIDANGILSVSASDKGTGKKQDITITGASTLPKDEVD
QMVQEAERFAKDDKEKRDAIDTKNQADSVVYQTEKQLKELGEKIPGEVKGKVEAKL
QELKDKIASGTTQEIKDTMAALNQEVMQIGQSMYNQPGAGAGAGPSPGGEDASSAD
SSSKDGDDVIDADFTDSK.

>Gene0336320.1

MAASTMALSSPAFAGKAVKLSPGASEVFGTGRVTMRKTVKPTGPSGSPWYGSDRVK
YLGPFSGEPPSYLTGEFPGDYGWDTAGLSADPETFARNRELEVIHCRWAMLGALGCV
FPELLARNGVKFGEAVWFKAGSQIFSEGGLDYLGNPSLVHAQSILAIWATQVILMGAV
EGYRVAGEGPLGEAEDLLYPGGSFDPLGLATDPEAFAELKVKEIKNGRLAMFSMFGFF
VQAIVTGKGPLENLADHLADPVNNNAWAFATNFVPGK.

>Gene0403280.1

MASSLTAAGTQPSAPSFSGLRRTCPKLDAAVSFSHRVNSSVRLVSSSQRSPRGVVAMA
GSGKFFVGGNWKCNGTKDTITKLVSDLNTATLESDVDVVVSPPFVYIDQVKSSLTDRI
EISGQNSWVGKGGAFTGEISVEQLKDIGCKWVILGHSERRHVIGEKDEFIGKKAAYAL

SEGLGVIACIGEKLEEREAGKTFDVCFDQLKAFADAVPSWDKVVVAYEPVWAIGTGK

VASPQQAQEVHVAVRDWLKKNVSEEVASKTRIIYGGSVNGGNCAELAKEEDIDGFLV

GGASLKGPEFATIVNSVTSKKVAA.

>Gene0023780.1

MGLCQLSVTTDKKRNISHAKSAIEEAASKGAKLVLLSEMWNTPYTKGSFRAFAEDID

AGGDASPSTAMLSEVSKRLEITIIGGSMPEKSGGRLYNTCCVFGSHGELKAKHRKIHL

FDIDIPGKLTYMESRTFTAGETPTVVDTDVGRIGIGICYDIRFQELAMMYAARGAHLLC

YPGAFNMTTGPLHWEILQRARATDNQLYVATCSSARDYGSCYVAWGHSSLVGPFGEV

IATTEHEEAIIMAEIDYSFLEQRRSSLPLNKQRRGDLYQLVDIQRSHTWSIVPLYLTVKE

QVPYACGEEARTCEGPICRREHTHNNASRDSVGPRTDTVESFTA.

>Gene0037720.1

METLKSLIPEHLLQTVKSSSSSVDDLLSSSSSLLRFLLGLPQFHQAVSELADPDLGCCG

KSQESSLDLKRRGNLCFRSRSFDDALRFYSKALRVARDNTLLASLFLNRANALHNLG

LLQESLRDCHRALRIDPFYSKAWFRRGKLNTLLGNYKDAFRDITVSISLESSPAGKKQ

LQNELLAIPDFQNKQTSENTLCYATADLPNVQSEVKLRCVSTKEKGRGMVSESDIEQP

SVIHVEEPFCVVISKSCRETHCHFCLNELPADSVPCPSCSVPVYCSESCQIQSGGTLSTN

EMVKNSTFQNLPDDIVEHIKGVTSAVRYYSETECVQEHQHECRGANWPAVLPSDAVL

AGRVIMKLINQGETPTDLSNLQEKLDLSHSYSKMSPESKLELHLLSIVLIWCLNKSCCP

DLSVCEASVSQTIILLSQIKVNSMAIVRMKSSGDSFKCIPAGNVSAKEPIQSLEQIRVGQ

ALYKIGSLFNHSCKPNIHLYFLSRGLVMRTTEFVPLGCPLELSYGPEVGKWDCKDRIRF

LEEEYFFHCRCCGCSQMNISDLVINGYCCVNSSCTGVVLDSSVAACESEKLNHFLAAP

KSLDHHFLVSEKVYAGVSEVASSLLIKPSGSLHIKADKCLKCGSRCEVENSHAEVNKA

WNHLRRVEELMNSGRANISVLSDCLRSIAVLRTSLHMYNKDIADAEDKVAQACYLAG

EMKGAREHCEASIKILKRLYGDEHVVIGNELVKLASIQLASGDSSGAWETTKRLSQIFS

KYYGSHAETLFSYLSCLKLEAAKAVNL.

>Gene0558360.1

MVDPANTVGIPVNPTPLLKDELDIVIPTIRNLDFLEMWRPFLQPYHLIIVQDGDPTKKI

HVPEGYDYELYNRNDINRILGPKASCISFKDSACRCFGYMVSKKKYIFTIDDDCFVAK

DPAGKAVNALEQHIKNLLCPSTPLFFNTLYDPYREGADFVRGYPFSLREGVSTAVSHG

LWLNIPDYDAPTQLVKPKERNARYVDAVMTIPKGTLFPMCGMNLAFDRDLIGPAMYF

GLMGDGQPIGRYDDMWAGWCIKVICDHLGLGVKTGLPYIYHSKASNPFVNLKKEYK
GIFWQEDIIPFFQNVKLSKEATTVQQCYIELSKMVKEKLSSLDPYFDKLADAMVTWIE
AWDELNPPATA.

>Gene0534360.1

MATIVQCLSSCAILNSKFKSLSLNGASSSSPTSSFSTRRGVCSSLSFAQSVSQCVAFSSG
NTWVQKKPVRQLTVCEAAPTKKADSAAKRARQAEKRRVYNKSKKSEARTRMKKVL
EALDGLKKKADAVPDEIVTVEKLIGEAYSAIDKAVKVRALHKNTGARRKSRLARRKK
AVEIHHGWYVPATAPEAATMAA.

>Gene0370290.1

MAKPMAIEVYNPNGKYRVVSTKPMPGTRWINLLVDQGCRVEICHLKKTILSVEDIIDL
IGNKCDGVIGQLTEDWGETLFSALSKAGGKAFSNMAVGYNNVDVEAANKYGIAVGN
TPGVLTETTAELAASLSLAAARRIVEADGFMRAGLYEGWLPHLFVGNLLKGQTVGVI
GAGRIGSAYARMMVEGFKMNLIYFDLYQSTRLEKFVTAYGQFLKANGEQPVTWKRA
SSMEEVLREADLISLHPVLDKTTYHLVNKERLAMMKKEAILVNCSRGPVIDEVALVD
HLRENPMFRVGLDVFEEEPFMKPGLADMKNAIVVPHIASASKWTREGMATLAALNV
LGRIKGYPIWSDPNRVDPFLNENASPPNASPSIVNSKALGLPVSKL.

>Gene0385090.1

MEGKEEDVRVGANKFPERQPIGTSAQSDKDYKEPPPAPLFEPGELASWSFWRAGIAEF
IATFLFLYITVLTVMGVKRSPSMCASVGIQGIAWAFGGMIFALVYCTAGISGGHINPAVT
FGLFLARKLSLTRAVYYIVMQCLGAICGAGVVKGFQPKQYQALGGGANTVAPGYTK
GSGLGAEIIGTFVLVYTVFSATDAKRNARDSHVPILAPLPIGFAVFLVHLATIPITGTGIN
PARSLGAAIIFNKDNAWDDHWVFWLDHSSVLHLLLFTT.

>Gene0274130.1

MVGGGFIQQILRRKLHSQSLATPVLSLFSSKKVNEDAGSSGVRALTLLGAGVTGLLSF
STVASADEAEHGLACPDYPWPHDGILSSYDHASIRRGHQVYQQVCASCHSMSLISYR
DLVGVAYTEEEAKAMAAEIEVVDGPNDEGEMFTRPGKLSDRLPQPYANESAARFANG
GAYPPDLSLITKARHNGQNYVFALLTGYRDPPAGISIREGLHYNPYFPGGAIAMPKML
NDEAVEYEDGVPATEAQMGKDVVSFLSWAAEPEMEERKLMGFKWIFLLSLALLQAA
YYRRLKWSVLKSRKLVLDVVN.

>Gene0288790.1

MRRIGFHNLNQQRTRRVLTSSSSSSSSTVLPHLAPSCQETILSPQDPKPKTDLPSSFSFLR
ETKPCISCPRNQVTDSVNRVKLRDGRFLAYKERGVPKNEAKYKIILIHGFGSSKEMNF
SASKELIEELGVYFLLYDRAGYGDSDPNPERSLQSEALDIEELAHNLQIGPKFYLIGVS
MGSYPTWSCLKHIPHRLAGVAFVAPVVNYGWRSLSKKLIKKDYRRGIIKWCFRLSKY
APGLLHWWISQKLFPSTTSVLESNQLYFNTSDIEVLKRTTGFPMLTKDKLRERSVFDTL
KDDFLAGFGQWDFDPSDVRMSGENSVHIWHGKEDKVVPFQLHRCILKKLPWIKYHE
IPHGGHLIVHYDVVRPIALARTFRILQWVLKLCKALYIDHEEDHVRYICRLKRRVRVSS
ASPLRFRDSFNREKLKMSKLQSEALREAITTIKGKSEDKKRNFVETIELQIGLKNYDPQ
KDKRFSGSVKLPHIPRPKMKVCMLGDAQHVEEAEKMGLDSMDVEALKKLNKNKKL
VKKLAKKYHAFLASESVIKQIPRLLGPGLNKAGKFPTLVSHQESLEGKVNETKATVKF
QLKKVLCMGVAVGNLSMEEKQLFQNVQMSINFLVSLLKKNWQNVRCLYLKSTMGPP
QRIF.

>Gene0276920.1

MSRRSLTLLKHLANSRTQTRSVTYMPRPGDGAPRAVTLIPGDGIGPLVTNAVEQVMEA
MHAPIYFEKYDVQGEMSRVPAEVMESIRKNKVCLKGGLKTPVGGGVSSLNVQLRKE
LDLFASLVNCFNLPGLPTRHENVDIVVIRENTEGEYAGLEHEVVPGVVESLKVITKFCS
ERIAKYAFEYAYLNNRKKVTAVHKANIMKLADGLFLESCREVAKKYPGITYNEIIVDN
CCMQLVAKPEQFDVMVTPNLYGNLVANTAAGIAGGTGVMPGGNVGADHAVFEQGA
SAGNVGKDSIVRENKANPVALLLSSAMMLRHLQFPSFADRLETAVKKVISEGKCRTK
DLGGQSTTQEVVDAVIAKLE.

>Gene0037100.1

MGSNSVVDMIQASSKVHFSGFHVNGHVNGLAQKAVSKETISASGEIQRQPFVIGVAG
GAASGKTTVCDMIIQQLHDQRVVLINQDSFYHSLTEEELARVHEYNFDHPDAFDTDH
LLSCMEKLRQGEAVDIPKYDCKTYKSSVFRRVNPTDVIILEGILLFHDPRVRRLMNMK
IFVCTDSDVRLERRIRRDTVENGRDIGTVLDQYSKFVKPAFDDFILPTKKYADIIIPRGG
DNHVAVDLIVQHICTKLGQHDLCKIYPNLYVIHSTFQIRGMHTLIRDAQTTKHDFVFYS
DRLIRLVVEHGLGHLPFTEKQVITPTGCVYSGVDFCKRLCGVSVIRSGESMENALRAC
CKGIKIGKILIHREGDNGQQLIYEKLPNDISERHVLLLDPILGTGNSAVEAINLLISKGVP
EGNIVFLNLISAPQGVHVVCKKFPRIKIVTSEIDDGLNEEFRVIPGMGELWIHPPSYPLQ
LLPKLSNESSLTLLVFPFKRPQVLGNSFELLSKSLLQLQKATMSAFVGKYADELIKTAK

YIATPGKGILAADESTGTIGKRFSSINVENIESNRQALRELLFTSPGALPCLSGVILFEET
LYQKTSDGKPFVEFLQENGVIPGIKVDKGVVDLAGTNGETTTQGLDSLGARCQEYYK
AGARFAKWRAVLKIGATEPSELSIQENAKGLARYAIICQENGLVPIVEPEVLTDGSHDIK
KCAAVTETVLSAVYKALNDHHVLLEGTLLKPNMVTPGSDSAKVAPEVIAEYTVTALR
RTVPPAVPGIVFLSGGQSEEEATLNLNAMNKLDLLKPWTLTFSFGRALQQSTLKAWGG
KKENVAKAQATFLVRCKANSDATLGKYIAGASGDSAASESLFVKGYSY.

>Gene0414220.1

MSAFTSKFADELIANAAYIGTPGKGILAADESTGTIGKRLASINVENVETNRRALRELL
FTAPGALPCLSGVILFEETLYQKSSDGKLFVDILKEGGVLPGIKVDKGTVELAGTNGET
TTQGLDGLGERCKKYYEAGARFAKWRAVLKIGENEPSELSIHENAYGLARYAVICQE
NGLVPIVEPEILVDGSHDIHKCAAVTERVLAACYKALSDHHVLLEGTLLKPNMVTPGS
DSAKVAPEVIAEHTVRALQRTVPAAVPAIVFLSGGQSEEEATKNLNAMNQLKTKKPW
SLSFSFGRALQQSTLKTWAGKEENVKAAQEALYVRCKANSEATLGTYKGDAKLGDG
AAESLHVKDYKY.

>Gene0037920.1

MSPASSLRRLAVGAAVIAAASGGGAVYLSPSVASSDRGGGPVLDSLRRRIGDPNASVP
SRSAQESALIGASASDPLDVLVIGGGATGSGVALDAVTRGLRVGLVEREDFSSGTSSRS
TKLIHGGVRYLEKAVFNLDYGQLKLVFHALEERKQLIENAPHLCHALPCMTPCFDWF
EVVYFWMGLKMYDLVAGPRLLHLSRYYSAQESAELFPTLARKGKDRSLRGTVVYYD
GQMNDSRLNVGLACTAALAGAAVLNHAEVVSLITDEATKRIVGARVRNNLTGKEFDS
YAKVVVNAAGPFCDSIRKMVDEDTKPMICPSSGVHIVLPDYYSPEGMGLIVPKTKDG
RVVFMLPWLGRTVAGTTDSNTSITPLPEPHEDEIQFILDAISDYLNIKVRRTDVLSAWS
GIRPLAMDPTAKSTESISRDHVVFEENPGLVTITGGKWTTYRSMAEDAVDAAIKSGKL
SPTNGCVTQKLQILGSHGWEPSSFTALAQQYVRMKKTYGGKVVPGAMDTAAAKHLS
HAYGSMADRVAIIAQEEGLGKRLAHGHPFLEAEVAYCARHEYCESAVDFIARRCRIAF
LDTDAAARALQRVVEILASEHKWDKSREKQELQKAKEFLQTFKSSKNAQFHDGKHN
.

>Gene0038290.1

MPPKTSGKAAKKSGKAQKNISKSDKKKKKHKRKESYAIYIYKVLKQVHPDTGISSKA
MSIMNSFVNDIFERIAAEASRLAHYNKRSTITSREVQTSVRLLLPGELAKHAVSEGTKA

VTKYTSSKYSTRYTEPVRTSRAERYPSPRAPRLRLTPPRSPYRYYKYSCTKRDAPRKQ
LATKAARKSAPATGGVKKPHRYRPGTVALREIRRYQKSTELLIRKLPFQRLVREIAQDF
KTDLRFQSSAVMALQEASEAYLVGLFEDTNLCAIHAKRVTIMPKDIQLARRIRGERA.

>Gene0038850.1

MAARQQPRGTGLGVQHEDFVPKSEWKNQPKAILLTIDLPGFTKEQIKVTYVHTSKML
KVTGERPLAGPRRWSRFNEVFSVPPNCLVDKIYGTFNNNSLTITMPKKTIRKMPDLPE
ASKTGDEKVEKLEEKRFLEESIRKAKEEEAEKKKKLHEEREAILRKLQEEAKTKEMA
ERRKGTGLEVQYLDFVPKYGWKDEREATLLIIDLPGFTKEQIKSTYVHTSNTLRVTGE
RPLAGPRRWSRFNEVFTVPHNCLVNKIHGNFNNNSLTITMPKKTIIEMPNLWETYKTV
AEKVEKVEKLEEKRLLEESKRKKEEEEAEKKKKLLEEKESILKKLQEEAKAKEMVES
MRLQEAAIAKERAEAKKLQEEANIKEMAEARKLQEAAIAKELAEARKLLEEAIAEEK
AEVRKLQEAAIAKEMAEARRLLEKAIEEEKAEARRLQEIAKENEIAEAKKLQEAKAK
EMAEERKLQEETIAKERADARNFNNNTLTITMPKETITKMPNLPETSKTMADKVEKL
EEKRLLEESTRKDKEAEAEKKKKLLEEKEGVLRKLQEEAKAKEMAEANKLQEEAIA
KEKAEARRVQEEAKAKEMVEARRLQEEVIAKEKAEARRLQEEAKAKEMVEARRLQ
EEVVAKEKAEARRLQEEAKAKEMAEARRLQAEAIAKEKAEARRLQEAAIANEKLLE
EAALEKKIQDRKSMDEFVRKEELKEKLSYSVLEENKTGKGIMEKIRGREITSEEKKLM
MNVGVSAFVIFALGAYVSYRPE.

>Gene0248160.1

MASFDEAPPGNSKAGEKIFRTKCAQCHTVDKGAGHKQGPNLNGLFGRQSGTTAGYS
YSAANKNKAVEWEEKTLYDYLLNPKKYIPGTKMVFPGLKKPQDRADLIAYLKEATA.

>Gene0168860.1

MAERGGERGAERGGDRGGFGRGFGGGRGDRGGRGGPRGRGGRRGGRPTEEEKWTP
VTKLGRLVQAGKIQKLEQIYLHSLPVKEYQIIDLLVGPTLKDEVMKIMPVQKQTRAG
QRTRFKAFVVVGDTNGHVGLGVKCSKEVATAIRGGIILAKLSVIPVRRGYWGNKIGKP
HTVPCKVTGKCGSVTVRMVPAPRGAGIVAARVPKKVLQFAGIDDVFTSSRGSTKTLG
NFVKATFDCLQKTYGFLTPEFWKETSFKKSPYQEYTDLLAEKGIATKAITEVEDQAAS.

>Gene0211680.1

MTATLKRFNYLSFINSPLLHNSNRHRLPRFFQPPNHLFSSQCGSLMEVFKAAFSEGSNA
SDRIAIKADGKSYSYGQLTSSALTISKLFHSDDAKNGGETRKCDGFGSLQGARVGIVA

KPSAEFVAGVLGTWFSGGVAVPLALSYPEAELLYVMSNSDISVLLSTEDHSETMKTIA

AKSDARFHLIPSVLNSTSETVTRNQFQDDSFEEDGKLLDDPALIVYTSGTTGKPKGVV

HTHKSINSQVRMLTEAWEYTSADHFLHCLPLHHVHGLFNALFAPLYARSSVEFLPKFS

VSGIWRRWRESYPVNDDKTDNPITVFTGVPTMYTRLIQGYEAMDQETKESSAFAAQK

LRLMMSGSSALPRPVMHQWESITGHRLLERYGMTEFVMAISNPLRGERKAGTVGKP

LPGVEAKIVQDKNDTDGVGEICVKSPSLFKEYWNLPEVTKESFTEDGYFKTGDAGRV

DEDGYFVILGRTSADIMKVGGYKLSALEIESTLLEHPTVAECCVLGLPDKNYGEAVTA

IIVAEAGAKRNREEESKPVMTLEELCGWAKDKLAPYKLPTRLLIWESLPRNAMGKVN

KKELKKSLDHQE.

>Gene0043930.1

MSEKAVTIRTRKFMTNRLLSRKQFVIDVLHPGRANVSKAELKEKLARMYEVKDPNAI

FVFKFRTHFGGGKSSGFGLIYDNVESAKKFEPKYRLIRNGLDTKIEKSRKQIKERKNR

AKKIRGVKKLIGNTPMVYLNKVVDGCLARIAAKLEMMEPCSSVKDRIAYSMIKDAE

DKGLITPGKSTLIEPTAGNTGIGLACIGAARGYKVILLMPSTMSLERRIILKALGAELHL

TDVKIGIQGMLEKTEEILSKTPGGFVPQQFENPANPEIHYRTTGPEIWRDSAGKVDILV

AGVGTGGTISGVGKFLKEMNKDIKVCAVEPAESPVLSGGERGPHLIQGIGSGIIPTNLE

LSIVDEIIQVSEGEEAIETAKLLALKEGLLVGISSGAAAAAALKVAKRPENAGKLIVVV

FPSGGERYLSTKLFESVRFEAENLPIE.

>Gene0045360.1

MAATSTAAAASSIMGTRVVSDINSGSSRFTARFGFGTKKAAAPKKAKTVISDRPLWFP

GAKSPEYLDGSLVGDYGFDPFGLGKPAEYLQFDLDSLDQNLAKNIAGDVIGTRTEAA

DPKSTPFQPYSEVFGLQRFRECELIHGRWAMLATLGALSVEWLTGVTWQDAGKVELV

DGSSYLGQPLPFSISTLIWIEVLVIGYIEFQRNAELDSEKRLYPGGKFFDPLGLASDPEK

KAQLQLAEIKHARLAMVAFLGFAVQAAATGKGPLNNWATHLSDPLHTTIIDTFSSS.

>Gene0133730.1

MYRLISSIASKARVARNCTSQIGSRLSSTRNYAAKDIKFGVEGRALMLRGVEELADAV

QVTMGPKGRNVIIEQSWGAPKVTKDGVTVAKSIEFKDRVKNVGASLVKQVANATND

VAGDGTTCATVLTRAIFTEGCKSVAAGMNAMDLRRGIKLAVDTVVTNLKSRARMIST

SEEIAQVGTISANGDREIGELIAKAMESVGKEGVITIQDGKTLFNELEVVEGMKIDRG

YISPYFITNQKNQKCELEDPLILIHEKKISNLNSMVKVLELALKSQRSLLIVAEDLESDA

LAVLILNKLRAGIKVCAVKAPGFGENRKANMHDLATLTGAQVITEELGMNLEKIDLS
MLGNCKKITVSKDDTVFLDGAGDKKAIGERCEQIRSMVEASESDYDKEKLQERLAK
LSGGVAVLKIGGASESEVGEKKDRVTDALNATKAAVEEGIVPGGGVALLYASKELDKL
STANFDQKIGVQIIQNALKTPVYTIASNAGVEGAVIVGKLLESDNPDLGYDAAKGEYV
DMVKSGIIDPVKVIRTALVDAASVSSLLTTTEAVVTEIPTKEDASPAMGGGGGGMGGM
GGMGGMGF.

>Gene0048660.1

MALRMWASSTANALKLSSSASKSHLLPAFSISRCFSSVLEGLKYATSHEWVKHDGSVA
TIGITDHAQDHLGEVVFVELPEEKSAVSKEKNFGAVESVKATSEILSPISGEVIEVNTKL
TDSPGLINSSPYEDGWMIKVKPSNPAELETLMGPKEYTKFCEEEDAAH.

>Gene0423510.1

MASVALRNPTSKRLLPFSTQIYSRCGGSISSSPSISHSIGGGDDLSPSSFGASLWRSMATF
TRNKPHVNVGTIGHVDHGKTTLTAAITKVLAEEGKAKAIAFDEIDKAPEEKKRGITIAT
AHVEYETAKRHYAHVDCPGHADYVKNMITGAAQMDGGILVVSGPDGPMPQTKEHIL
LARQVGVPSLVCFLNKVDVVDDPELLELVEMELRELLSFYKFPGDDIPIIRGSALCALQ
GTNDEIGRQAILKLMDAVDEYIPDPVRVLDKAFLMPIEDVFSIQGRGTVATGRIEQGVI
KVGEEVEILGLKDGPPMKSTVTGVEMFKKILDNGQAGDNVGLLLRGLKREDIQRGM
VIAKPGSCKTYKKFEAEIYVLTKDEGGRHTAFLSNYRPQFYLRTADITGRVELPDDVK
MVMPGDNVTAVFELIMPVPLEIGQRFALREGGRTVGAGVVSKVMT.

>Gene0444870.1

MADGEDIQPLVCDNGTGMVKAGFAGDDAPRAVFPSIVGRPRHTGVMVGMGQKDAY
VGDEAQSKRGILTLKYPIEHGIVSNWDDMEKIWHHTFYNELRVAPEEHPVLLTEAPLN
PKANREKMTQIMFETFNVPAMYVAIQAVLSLYASGRTTGIVLDSGDGVSHTVPIYEGY
ALPHAILRLDLAGRDLTDSLMKILTERGYMFTTTAEREIVRDIKEKLAYVALDFEQELE
TAKSSSSVEKNYELPDGQVITIGAERFRCPEVLFQPSLIGMEAPGIHETTYNSIMKCDV
DIRKDLYGNIVLSGGSTMFPGIADRMSKEITALAPSSMKIKVVAPPERKYSVWIGGSIL
ASLSTFQQMWISKGEYDESGPSIVHRKCF.

>Gene0052470.1

MVAMASASGSALCFADASSSPAIRRDLGSFCLPPSTVTFGFVDKPIINLQRLRLSRLKP
RAANATAVENGKQDSDEVPTPVVIIDQDSDPDATVVEVTFGDRLGALLDTMNALKNL

GLNVVKANVYLDSSGKHNKFAITKADSGRKVEDPELLEAIRLTVINNLLEFHPESSSQ
LAMGAAFGVLPPTEQVDVDIATHIKIEDDGPDRSLLYIETADRPGLLVELVKNITDISVA
VESGEFDTEGLLAKVKFHVSYRNKALIKPLQQVLANSLRYFLRRPSTDDSSF.
>Gene0280990.1
MATKPAKQSVQCFGRKKTAVAVTHCKPGCGMIKLNGSPIELFQPEILRFKIFEPVLLLG
KHRFAGVDMRIRVNGGGHTSQVYAIRQSIAKALVAFYQKYVDEQSKKEVKDILIRYD
RTLLVADPRRCEPKKFGGRGARSRFQKSYR.
>Gene0401760.1
MAFRNFWEISRDRGDQSYDGGSTDLESSPIWGGDWEFGKSSIRFGRGEEERAWSRNT
RWEQPRYQVQDDRNRYQRGARNARSAVERERQEPREEGEIGVIERGPRNLHKVEKPQ
LALPEPNGAKLAISGASATGMDVEGELGDSTEIENLVNGLEQAMDLVGNGNAVGDNV
AMTLDGSVAIEGRKEELSEGGEEELGGNGDQDAQVGEEEKKKGARKVLFKKPPGIA
MGTSKLRLVQAVLSPRKNGASKSSKRQGGGEGLKQAEEKGPLNPKNSAKPG.
>Gene0195530.1
MSRQVARLLGSLSRRCSSTSSEAIPPTLSSFTQSRSFASDPPPPAAVFVDKNTRVMCQGI
TGKNGTFHTEQAIEYGTKMVAGVTPKKGGTEHLGLPVFNSVAEAKAETKANASVIYV
PAPFAAAAIMEGIEAELDLIVCITEGIPQHDMVRVKHALNSQSKTRLIGPNCPGIIKPGE
CKIGIMPGYIHKPGKIGIVSRSGTLTYEAVFQTTAVGLGQSTCVGIGGDPFNGTNFVDC
LEKFFVDPQTEGIVLIGEIGGTAEEDAAALIKESGTDKPVVAFIAGLTAPPGRRMGHAG
AIVSGGKGTAQDKIKSLNDAGVKVVESPAKIGAAMYDLFKERGLLKQ.
>Gene0071120.1
MSLTIPTNLVLNKKTLTQSLPKSTARFVCSTDDNKSLKAFSAAVALSSILLSSPMPAAAD
ISGLTPCKESKQFAKREKQQIKKLQSSLKLYAPESAPALALNAQIEKTKRRFDNYGKY
GLLCGADGLPHLIVNGDQRHWGEFITPGLLFLYIAGWIGWVGRSYLIAISDEKKPAMK
EIIIDVPLASRLIFRGFIWPVAAYRALLNGDLIAKDV.
>Gene0403540.1
MALSVSNLASSLSSLSFSSQVSHGPNALSFPRSNYLFSLPAKSPRRASLSITATVAAPAE
VSEDDTMELKKYVKSRLPGGFAAQKIIGTGRRKCAIARVVLQEGTGKVIINYRDAKE
YLQGNPLWLQYVKVPLVTLGYENSYDVFVKAHGGGLSGQAQAITLGVARALLKVSA
DHRSPLKKEGLLTRDARVVERKKVGLKKARKAPQFSKR.

>Gene0057450.1

MLNRTTLVSSSSVSLLPNSKPFFSVKAFSGFRSSSFSGGIVRRIDHKPLRVMTPNLTPRA

MAAQQLENADQLIDSVETFIFDCDGVIWKGDKLIEGVPETLDMLRAKGKRLVFVTNN

STKSRKQYGKKFETLGLNVNEEEIFASSFAAAAYLQSINFPKDKKVYVIGEEGILKELD

LAGFQYLGGPDDGKKQIELKPGFLMEHDNDVGAVVVGFDRYFNYYKIQYGTLCIRE

NPGCLFIATNRDAVTHLTDAQEWAGGGSMVGALVGSCQREPLVVGKPSTFMMDYLA

DKFGIEKSQICMVGDRLDTDILFGQNGGCKTLLVLSGVTSISMLESPENKIQPDFYTSK

ISDFLSLKAANV.

>Gene0451630.1

SLVANEEFQHILRVLNTNVDGKQKIMFALTSIKGIGRRLANIVCKKADVDMNKRAGEL

SAAEIDNLMTIVANPKQYKIPDWFLNRQKDYKDGKYSQVVSNALDMKLRDDLERLK

KIRNHRGLRHYWGLRVRGQHTKTTGRRGKTVGVSKKR.

>Gene0058100.1

MTSRVMMILLKNTWIQDPLHVRPIAHAIWDPHFGQPAVEAFTRGGALGPVNIAYSGV

YQWWYTIGLRTNEDLYTGALFLLFLSALSLIGGWLHLQPKWKPRVSWFKNAESRLN

HHLSGLFGVSSLAWTGHLVHVAIPASRGEYVRWNNFLSVLPHPQGLGPLFTGQWNLY

AQNPDSSSHLFGTSQGSGTAILTLLGGFHPQTQSLWLTDMAHHHLAIAILFLIAGHMY

RTNFGIGHSIKDLLEAHIPPGGRLGRGHKGLYDTINNSIHFQLGLALASLGVITSLVAQH

MYSLPAYAFIAQDFTTQAALYTHHQYIAGFIMTGAFAHGAIFFIRDYNPEQNEDNVLA

RMLDHKEAIISHLSWASLFLGFHTLGLYVHNDVMLAFGTPEKQILIEPIFAQWIQSAHG

KTSYGFDVLLSSTNGPAFNAGRSIWLPGWLNAINENSNSLFLTIGPGDFLVHHAIALGL

HTTTLILVKGALDARGSKLMPDKKDFGYSFPCDGPGRGGTCDISAWNTFYLAELIDTL

AWAHERTPLANLIRWKDKPVALSIVQARLVGLAHFSDPICIIIIDNKRNLSIMAKKSLIY

REKKRQKLEQKYHLIRRSLKKEISEIPSLSEKWKIHGKLQSPPRNSAPTQLTIETLDYLD

TSFGKWFRHVYCQGQQDQAG

>Gene0059360.1

MAQAMTSMAGLRGASQALQTNDSNRLSISRVTVGSKRTGLVIRAQQNESVPESSRRS

VIGLVAAGLVGGSFVKSAVAAEALAIKVGPPPPPSGGLPGTDNSDQARDFSLKLKDRF

YLQPLSPTEAAARAKESAKEIINVKTLIDKKAWPYVQNDLRLRASYLRYDLNTVISAK

PKEEKQSLKELTGKLFQTIDNLDYAARSKSSPDAEKYYSETVSSLNNVLAKIG.

>Gene0059550.1

MAKTLSSICFTTLLLVVLLISAGIPKSEATCTKYLGEAILAYPCKESYCEAKCAESYHES

CRGECEDHDHHHGVHLTNDHDDHCHCYGRY.

>Gene0441070.1

MGTTLSLSKTKGLLQFSLSPELELAGSLLTSRNVFVQILRSTFPYQPGPTTTTSGEMHK

SLKRRTITTKSLKRRTITTIQTKGLDFSGLMDDVKNAPEGSFFLLHACAHNPTGVDPTE

EQWREISQLFKQFSGQKPFCILRHGLSRLASGDPVRDAKSIRIFLEDGHHIGISQSYAKN

MGLYGQRVGCLSVLCEDEKQAVTVKSQLQQLARPMYSNPPLHGAQIVSTILGDPALK

GLWLKEVKVMADRIIGMRTALRESLEKLGSPLSWEHVTKQIGMFCYSGMTPEQVDRL

TSEYHIYMTRNGRISMAGVTTGNVGYLANAIHEVTKSS.

>Gene0061390.1

MGLPWYRVHTVVLNDPGRLLSVHIMHTALVAGWAGSMALYELAVFDPSDPVLDPM

WRQGMFVIPFMTRLGITNSWGGWNITGGTITNPGLWSYEGVAAAHIVFSGLCFLAAI

WHWVYWDLEIFCDERTGKPSLDLPKIFGIHLFLSGVACFGFGAFHVTGLYGPGIWVSD

PYGLTGKVQPVNPAWGVEGFDPFVPGGIASHHIAAGTLGILAGLFHLSVRPPQRLYKG

LRMGNIETVLSSSIAAVFFAAFIVAGTMWYGSATTPIELFGPTRYQWDQGYFQQEIYRR

VSAGLAENQSVSEAWSKIPEKLAFYDYIGNNPAKGGLFRAGSMDNGDGIAVGWLGH

PVFRNKEGRELFVRRMPTFFETFPVVLVDGDGIVRADVPFRRAESKYSVEQVGVTVE

FYGGELNGVSYSDPATVKKYARRAQLGEIFELDRATLKSDGVFRSSPRGWFTFGHASF

ALLFFFGHIWHGSRTLFRDVFAGIDPDLDAQVEFGAFQKLGDPTTKRQAV.

>Gene0509350.1

MVSGSGICSKRVVVDARHHMLGRLASIVAKELLNGQKVVIVRCEEICLSGGLVRQKM

KYMRFLRKRMNTKPSHGPIHFRAPSKIFWRTVRGMIPHKTKRGAAALARMKVFEGV

PPPYDKVKRMVIPDALKVLRLQAGHKYCLLGRLSSEVGWNHYDTIKELETKRKERS

QVVYERKKQLNKLRAKAEKVAEEKLGAQLEILAPVKY.

>Gene0500550.1

MVAAGIDMDEGALEIGMEYRTVSGVAGPLVILEKVKGPKYQEIVNIRLGDGSMRRGQ

VLEVDGEKAVVQVFEGTSGIDNKFTTVQFTGEVLKTPVSQDMLGRIFNGSGKPIDNGP

PILPEAYLDISGSSINPSERTYPEEMIQTGISTIDVMNSIARGQKIPLFSAAGLPHNEIAAQ

ICRQAGLVKRLEKTENLIEDHGEDNFAIVFAAMGVNMETAQFFKRDFEENGSMERVT

LFLNLANDPTIERIITPRIALTTAEYLAYECGKHVLVILTDMSSYADALREVSAAREEVP
GRRGYPGYMYTDLATIYERAGRIEGRKGSITQIPILTMPNDDITHPTPDLTGYITEGQIY
IDRQLHNRQIYPPINVLPSLSRLMKSAIGEGMTRRDHSDVSNQLYANYAIGKDVQAMK
AVVGEEALSSEDLLYLEFLDKFERKFVMQGAYDTRNIFQSLDLAWTLLRIFPRELLHRI
PAKTLDQFYSRDSTS.

>Gene0067660.1

MAQTMLLTSGISANHFLRNKNPLAQPKVHHLFLSGNSPVTLPSRRPSLVPLAIFKPKTK
AAPKKAEKVKPKVEDGIFGTSGGIGFTKQNELFVGRVAMIGFAASLLGEALTGKGILA
QLNLETGIPIYEAEPLLLFFILFTLLGAIGALGDRGKFVDDPPTGLEKAVIPPGKGVRSA
LGLKEQGPLFGFTKANELFVGRLAQLGIAFSLIGEIITGKGALAQLNI

>Gene0260070.1

MSSQICRSASKAARSLLSSAKNARFFSEGRAIGAAAAVTASGKMPLYASNFARSSGSS
KSWITGLLALPAAAFMVQDQEVFAAEMERTFIAIKPDGVQRGLISEIISRFERKGFKLV
GIKVVVPSKDFAQKHYHDLKERPFFNGLCDFLSSGPVIAMVWEGEGVIRYGRKLIGAT
DPQKSEPGTIRGDLAVVVGRNIIHGSDGPETAKDEINLWFKPQELVSYTNNAEKWIYG
DN.

>Gene0068920.1

MLLRTSLQQGKNNLRPRFLRCLLSTMSSTQPPRVPNLIGGSFVDSQASSHIDVINPATQ
EVVSQVPLTTNEEFKAAVSSAKKAFPSWRNTPITTRQRVMLKFQELIRKNMDKLALSI
TTEQGKTLKDAHGDIFRGLEVVEHACGMATLQMGEYVPNVSNGVDTYSLREPLGVC
AGICPFNFPAMIPLWMFPIAVTCGNTFVLKPSEKDPGASVLLAELAMEAGLPDGVLNI
VHGTNDTVNAICDDDDIRAVSFVGSNTAGMHIYARAAAKGKRIQSNMGAKNHGVVL
PDANVDATLNALLAAGFGAAGQRCMALSTVVFVGNSKSWEDKLVERAKALKVSCG
TEPDADLGPVISIQAKERICRLIQSGVDDGAKLLLDGRNIVVPGYEKGNFIGPTILSGVT
PDMECYKEEIFGPVLVCMEASSFDEAIDILNRNKYGNGAAIFTASGAAARKFQMEIEA
GQIGINVPIPVPLPFFSFTGNKASFAGDLNFYGKAGVDFFTQIKTVTQQWKDIPTSVSL
AMPTSQKQ.

>Gene0071260.1

SRRKTREPKEETVTLGPAVRDGEQVFGVVHIFASFNDTFIHVTDLSGRETLVRITGGMK
VKADRDESSPYAAMLAAQDVAQRCKELGITAMHVKLRATGGNKTKTPGPGAQSALR

ALARSGMKIGRIEDVTPIPTDSTRRKGGRRGRRL.

>Gene0122630.1

MAMAMALRRLSSSVDKPIRPLIRSSSCYMSSLPSEAVDDKERSRVTWPKQLNASLEEV

DPEIADIIEHEKARQWKGLELIPSENFTSVSVMQAVGSVMTNKYSEGYPGARYYGGN

EFIDMAETLCQKRALEAFRLDPEKWGVNVQPLSGSPANFHVYTALLKPHERIMALDL

PHGGHLSHGYQTDTKKISAVSIFFETMPYRLDESTGFIDYDQMEKSATLFRPKLIVAGA

SAYARLYDYARIRKVCNKQKAVMLADMAHISGLVAAGVIPSPFEYADVVTTTTHKSLR

GPRGAMIFYRKGVKEINKQGKEVLYDFEDKINQAVFPGLQGGPHNHTITGLAVALKQ

ATTSEYKAYQEQVLSNSAKFAQTLMEKGYELVSGGTDNHLVLVNLKPKGIDGSRVEK

VLEAVHIASNKNTVPGDVSAMVPGGIRMGTPALTSRGFVEEDFAKVAEYFDKAVKLA

LKVKSEAQGTKLKDFVSAMESSSAIQSEIAKLRHDVEEFAKQFPTIGFEKETMKYKN.

>Gene0359240.1

MMALALRRLSSSLKKPIFSNGVSLRSMSSLPTSAMADSERSRSSWIKQLNAPLEEIDPE

IADIIELEKARQWKGFELIPSENFTSASVMEAVGSVMTNKYSEGYPGARYYGGNEYID

MAESLCQKRALEAFHLDPSKWGVNVQSLSGSPANFQVYTALLKPHERIMALDLPHG

GHLSHGYQTDTKKISAVSIFFETMPYRLDESTGYIDYDQLEKSAVLFRPKLIVAGASAY

ARLYDYARIRKVCDKQKAVMLADMAHISGLVAAGVIPSPFEYADVVTTTTHKSLRGP

RGAMIFFRKGLKEVNKQGKEVMYDYEDRINAAVFPGLQGGPHNHTITGLAVALKQV

KSPEYKAYQDQVLRNCSKFAETLLSKGYDLVSGGTDNHLVLVNLKNKGIDGSRVEKV

LESVHIAANKNTVPGDVSAMVPGGIRMGTPALTSRGFIEEDFAKVAEYFDLAVKIALKI

KAESQGTKLKDFVATMQSNEKLQSETAKLREMVEEYAKQFPTIGFEKETMRYKE.

>Gene0074190.1

MAASFLWASRVASHLRISVAQRGFSSVVLKDLKYAESHEWVKIDGNKATFGITDHLPD

LGRSVSQGESFGAVESVKATNDINSPVSGTVVEVNEVNTSPYEEGWILKVELSDAAEA

EKLMDSDKYSKFCKEEDAKH.

>Gene0541190.1

MASKLIQVQSKACEASKFVAKHGTSYYRQLLEKNKHYIQEPASVDKCQELSKQLLYT

RLASIPGRCETLGKEVDYAKNLWKNRTDLKVEDAGVAALFGLECFAWYCAGEIIGRG

FTFTGYYP.

>Gene0384860.1

MSVAAKGKVCVTGAGGFLASWVVDLLLSKDYFVHGTVRDPDNEKYSHLKKLEKAG
DKLKLVKADLLDYASLQSAIAGCIGVFHVASPVPSSSVPNPEVEVMAPAVDGTLNVLK
ACVEANVKRVVYVSSAAALMMNPNWSKDRVIDESCWSDLEFCKRTENWYCASKTQ
AESEAFEFAKRTGISLVSICPTMVFGPVLQQHTVNASTLALAKLLKEGFESRENQVRLI
VDVRDVAQALLLVYEKPEAEGRYICTAHKAKEKDVVEKLKSLYPNYNYPKSYVEVE
ERSTMTSEKLQKLGWSFRPLEETLVDSVESYRKAKILD.

>Gene0121430.1

VKFTSDELRRIMDYKHNIRNMSVIAHVDHGKSTLTDSLVAAAGIIAQEVAGDVRMTD
TRADEAERGITIKSTGISLYYEMTDASLKSFTGARDGNEYLINLIDSPGHVDFSSEVTA
ALRITDGALVVVDCIEGVCVQTETVLRQALGERIRPVLTVNKMDRCFLELQVDGEEA
YQTFQRVIENANVIMATYEDPLLGDVQVYPEKGTVAFSAGLHGWAFTLTNFAKMYAS
KFGVDETKMMERLWGENFFDPATRKWSGKNTGSATCKRGFVQFCYEPIKQIIATCMN
DQKDKLWPMLQKLGVQMKSDEKELMGKPLMKRVMQTWLPASTALLEMMIFHLPSP
HTAQRYRVENLYEGPLDDQYATAIRNCDPNGPLMLYVSKMIPASDKGRFFAFGRVFSG
KVSTGMKVRIMGPNFVPGEKKDLYVKSVQRTVIWMGKRQETVEDVPCGNTVAMVG
LDQFITKNATLTNEKEVDAHPIRAMKFSVSPVVRVAVQCKVASDLPKLVEGLKRLAKS
DPMVVCTMEESGEHIVAGAGELHLEICLKDLQDDFMGGAEIVKSDPVVSFRETVLER
SVRTVMSKSPNKHNRLYMEARPLEDGLAEAIDDGRIGPRDDPKIRSKILAEEFGWDK
DLAKKIWAFGPETTGPNMVVDMCKGVQYLNEIKDSVVAGFQWASKEGPLCDENMR
GICFEVCDVVLHSDAIHRGGGQVIPTARRVIYASQLTAKPRLLEPVYMVEIQAPEGALG
GIYSVLNQKRGHVFEEMQRPGTPLYNIKAYLPVVESFGFSSQLRAATSGQAFPQCVFD
HWEMMSSDPLEAGSQASTLVADIRKRKGMKEQMTPLSDFEDKL.

>Gene0238450.1

MMKRLVPTFNRILVQRVIQPAKTESGILLPEKASKLNSGKVIAVGPGSRDKDGKLIPVS
VKEGDTVLLPEYGGTISSGMRMCWEPCTRI

>Gene0083450.1

MIKTFALASSAHRILYPKYSSTFSSHHILRFSLSDRRTFSAMAGAGGDEFVKGNVYPN
GVAVITLDRTKALNAMNLEMDLKYKSFLDEWESDPRVKCVIIEGSTPRAFCAGMDIK
GVAAEIQKDKNTPLVQKVFTAEYTLICAIAGYKKPYISLMDGITMGFGLGLSGHGRYR
VITERTVLAMPENGIGLFPDVGFSYIAAHSPGGGSVGAYLGLTGKRISTPSDALFVGLG

THYVPSEKLASLKEAILSANLSADPNQDIEAALSKYSGNTESEAHLKSLLPQIESAFSS
NKSVKETIEELKKYQQSTEPSVVEWAKEALKGLEKGAPFSLYLTQKYFSNVACAKGK
AENELATLNGVMKTEYRIALRSALRGDFAEGVRAVLIDKDQNPKWNPASVEEVDESE
VEALFKPLSSEVEELKV.
>Gene0496910.1
ASEKKLSNPMRDIKVQKLVLNISVGESGDRLTRASKVLEQLSGQTPVFSKARYTVRSF
GIRRNEKIACYVTVRGDKAMQLLESGLKVKEYELLRRNFSDTGCFGFGIQEHIDLGIK
YDPSTGIYGMDFYVVLERPGYRVARRRRCKTRVGIQHRVTKDDAMKWFQVKYEGVI
LNKSQNITG.
>Gene0086470.1
MWGILRQRGIDGGGLSLRRTRSASVSARSYAAGSKEMTVRDALNSAIDEEMSADPKV
FVMGEEVGQYQGAYKITKGLLEKYGPERVYDTPITEAGFTGIGVGAAYAGLKPVVEF
MTFNFSMQAIDHIINSAAKSNYMSAGQINVPIVFRGPNGAAAGVGAQHSQCYAAWY
ASVPGLKVLAPYSAEDARGLLKAAIRDPDPVVFLENELLYGESFPISEEALDSSFCLPIG
KAKIEREGKDVTIVTFSKMVGFALKAAEKLAEEGISAEVINLRSIRPLDRETINASVRK
TSRLVTVEEGFPQHGVCAEICASVVEESFSYLDAPVERIAGADVPMPYAANLERLALP
QVEDIVRAAKRACYRSK.
>Gene0554050.1
MAPKAGKKPAEKKPAAAAEKPAEEVAEKAPAEKKPKAGKKLPKEAGAIDKKKKRNK
KSIETYKIYIFKVLKQVHPDIGISSKAMGIMNSFINDIFEKLAQEASKLARYNKKPTITS
REIQTAVRLVLPGELAKHAVSEGTKAVTKFTSS.
>Gene0089040.1
MAAVQDCPGNLKRQVEKLFDASLRSTVPDETGVQTEVTASLPGKPGDYQCNNAMGL
WSIIKGKGTQFKGPPAVGQALLKNLPTSEMVESCSVAGPGFVNVILSSKWMAKSIETM
LMDGIDTWAPSLPVKRAVVDFSSPNIAKEMHVGHLRSTIIGDTLARMLEYSKVEVLRR
NHVGDWGTQFGMLIEYLFEKFPDTESVTETAIGDLQSFYKESKSKFDADPEFKEKAQ
KAVVRLQGGDAIYRKAWTKICDISRAEFAKVYQRLRVELEEKGESFYNPYIAKVIEEL
NSKGLIEESEGARVIFLEGFNIPLMVVKSDGGFNYASTDLTALWYRLNEEKAEWIVYV
TDVGQQQHFSMFFKAARKAGWLPESDKTYPRVDHVGFGLVKGEDGKRFRTRSSEVV
RLVDLLDEAKTRSKTALIERGKDKEWTPEELDQTAEAVGYGAVKYADLKNNRSTSYT

FNFDQMLSDKGNTAVYLLYAHARICSIIRKSGKDIDELKKTGKLVLDHPEERALGLHL
LRFAETVEAACANLLPHVLCEYLYELSERYTSFYSVHQVIGSAEEASRLLLCEATAIVM
RKCFHLLGITPVYKI.

>Gene0089880.1

MSLLLQRALWRIMASRRPRLFSSPLSPSLHRHCSSLSPPPRINFQLSKVLSQGLIQRNAI
STISTESFQESATSKGYSSEQIQVLEGLDPVRKRPGMYIGSTGPRGLHHLVYEILDNAID
EALAGYASKVDVVLHADGSVSIMDDGRGIPTDLHPATKKSSLETVLTVLHAGGKFGG
TSSGYSVSGGLHGVGLSVVNALSEPLSSFDHNTIAGRIRELAFLNPKVTISLKKEDDDP
EKNQYTEHFYAGGLSEYVSWLNTDKNPIHDVLGFRKEINGATINVALQWCSDAYSDT
MRGYANSIRTIDGGTHIEGVKASLTRTLNTLAKKSKAVKEKDINLSGEHVREGLTCIVS
VKIPNPEFEGNPEVRKIVDQSVQEYLTDYLELHPDVLERIISKSLNAYKAALAAKRAR
ELVRSKSVLKSSSLPGKLSDCSSTNPEESEIFIVEGDSAGGSAKQGRDRRFQAILPLRGK
ILNIERKDEAAMYKNEEIQNLILALGLGVKGEDFKLENLRYHKIIILTDADVDGAHIRT
LLLTFFFRYQRALFDAGCIYVGVPPLFKVERGKQAQYCYDDADLKKITADFPANASYS
TQRFKGLGEMMPEQLWETTMNPETRILKQLVVDDIAEANMTFSYLMDARVDVRKEL
IKNAATRINLQHLDI.

>Gene0090500.1

MASLSSMQMVHTSQIGVKSQLVSANRTSQSVCVGARSSGSALSSRLHYAASFPLKKQ
FSGAYATIKNQRTACVKSMAAEEEEVIEPQAKVTNKVYFDVEIGGEVAGRIEMGLFGD
VVPKTVENFRVLCTGEKKYGYKGSSFHRIIKDFMIQGGDFTEGNGTGGISIYGAKFED
ENFTLKHTGPGILSMANAGPNTNGSQFFICTVKTPWLDGKHVVFGQVIKGMKLVRTL
ESQETRAFDVPKKGCRIYACGELPLDA.

>Gene0525110.1

MASRRVLSSLLRSSSGRSAAKFTSRNPRLPSPSPARCAAPFGNLLGRVAEYSTSSPAPPS
SAAPAKDEAKKTYDYGGKGAIGKVCQVIGAIVDVRFEDQEGLPPIMTSLEVQDHPTR
LVLEVSHHLGQNVVRTIAMDGTEGLVRGRRVLNTGAPITVPVGRATLGRIMNVLGEPI
DERGEIKTEHYLPIHRDAPALVDLATGQEILATGIKVVDLLAPYQRGGKIGLFGGAGV
GKTVLIMELINNVAKAHGGFSVFAGVGERTREGNDLYREMIESGVIKLGEKQSESKCA
LVYGQMNEPPGARARVGLTGLTVAEYFRDAEGQDVLLFIDNIFRFTQANSEVSALLGR
IPSAVGYQPTLASDLGALQERITTTKKGSITSVQAIYVPADDLTDPAPATTFAHLDATTV

LSRQISELGIYPAVDPLDSTSRMLSPHILGEEHYNTARGVQKVLQNYKNLQDIIAILGM
DELSEDDKLTVARARKIQRFLSQPFHVAEIFTGAPGKYVDLKENINSFQGLLDGKYDD
LPEQSFYMVGGIDEVVAKAEKISKEAAA.

>Gene0094450.1

MASTSLLKASPVLDKSEWVKGQSVLFRQPSSAAVVIRNRATSLTVRAASSYADELVKT
AKTIASPGRGILAMDESNATCGKRLDSIGLENTEANRQAYRTLLVSAPGLGQYISGAIL
FEETLYQSTTEGKKMVDVLVEQNIVPGIKVDKGLVPLVGSNNESWCQGLDGLSSRTA
AYYQQGARFAKWRTVVSIPNGPSALAVKEAAWGLARYAAISQDSGLVPIVEPEILLDG
EHDIDRTYEVAEKVWAEVFFYLAQNNVMFEGILLKPSMVTPGAESKDRATPEQVASY
TLKLLRNRIPPAVPGIMFLSGGQSELEATLNLNAMNQAPNPWHVSFSYARALQNTCLK
TWGGRAENVNAAQTTLLARAKANSLAQLGKYTGEGESEEAKEGMFVKGYTY.

>Gene0094930.1

MASRRVSSMLSRSFMSSPSLFALRGKHHNMNRGVYGYSNVAAGEDTITPPVKVEHTQ
LLIGGKFVDAASGKTFPTLDPRTGEVIAQVAEGDVEDVNRAVSAARKAFDEGPWPRM
TAYERSKILLRFADLVDKHNDEIAAIETWDNGKPFEQSSKIEVPMLARVFRYYAGWAD
KIHGMTVPGDGSHHVQTLHEPIGVAGQIIPWNFPLLMLSWKLGPALACGNTVVLKTA
EQTPLSALLVGRLLHEAGLPEGVVNIVSGFGPTAGAAIASHMDIDKVAFTGSTDVGKII
LQLASKSNLKAVTLELGGKSPFIVCEDADVDQAVELAHFALFFNQGQCCCAGSRTFV
HERVYDEFVEKAKARAIKRAVGDPFKSGIEQGPQVDSEQFKKILKFIKHGVESGATLQ
AGGDRFGSKGYYIQPTVFSDVKDDMLIATDEIFGPVQTILKFKNLDEVIARANNSRYG
LAAGVFTQNLDTANRLMRALRVGSVWINCFDVFDATIPFGGYKMSGIGREKGIYSLN
NYLQVKAVVTAIKNPAWL.

>Gene0460730.1

MMWRNIAGFSKAAAAAARTHGSRRYLSSAIPGPCIVHKRGADILHDPWFNKDTGFPL
TERDRLGLRGLLPPRVISFEQQYARFIESFRSLERNTQGQPDNVVSLAKWRILNRLHDR
NETLYYRVLIDNIKDFAPIIYTPTVGLVCQNYSGLYRRPRGMYFSAKDKGEMMSMIYN
WPAHQVDMIVITDGSRILGLGDLGVQGIGIPIGKLDMYVASAGINPQRVLPIMLDVGT
NNQKLLQNPLYLGLRQPRLEGEEYLEIVDEFMEAAFTRWPKAVVQFEDFQAKWAFET
LDRYRKKFCMFNDDVQGTAGVALAGLLGTVRAQGRPLSDFVNQKIVVVGAGSAGL
GVTKTAVQAVARMAGISFAEATKNFYLIDKDGLVTTERSKLDPAVVPFAKNPAEIREGA

SIVEVVKTVRPHVLLGLSGVGGIFNEEVLKAMRESDSCKPAIFAMSNPTLNAECTAAD
AFKHAGENIVFGSGSPFENVQLENGSVGHVNQANNMYLFPGIGLGTLLSGARIVTDG
MLLAAAECLASYMTDEEVQKGILYPSINNIRHITAEVGAAVLRAAVTDDIVEGHGDVG
PRDLSHMSKEETVDYITRNMWFPIYSPLVHEK.

>Gene0330430.1

MATSAIQQSSFAGQTALKPSNDLLRKVGVSGGGRVTMRRTVKSTPQSIWYGPDRPKY
LGPFSENTPSYLTGEYPGDYGWDTAGLSADPETFAKNRELEVIHSRWAMLGALGCTF
PEILSKNGVKFGEAVWFKAGSQIFSEGGLDYLGNPNLIHAQSILAIWACQVVLMGLIE
GYRIGGGPLGEGLDPLYPGGAFDPLNLAEDPEAFAELKVKELKNGRLAMFSMFGFFV
QAIVTGKGPIENLFDHLADPVANNAWSYATNFVPGK.

>Gene0338490.1

MAEGLVLKGTMRAHTDQVTAIATPIDNSDIIVSASRDKSIILWKLTKDDKSYGVAQRRL
TGHSHFVEDVVLSSDGQFALSGSWDGELRLWDLAAGVSTRRFVGHTKDVLSVAFSL
DNRQIVSASRDRTIKLWNTLGECKYTIAEGGEGHGDWVSCVRFSPNTLQPTIVSASW
DKTVKVWNLSNCKLRSTLAGHSGYVSTVAVSPDGSLCASGGKDGVVLLWDLAEGK
KLYSLEANSVIHALCFSPNRYWLCAATEQGIKIWDLESKSVVEDLKVDLKAEAEKSD
GSGTAGNKKKVIYCTSLSWNADGSTLFSGYTDGVIRVWGIGRY.

>Gene0141450.1

MAARSSLIRFAFVCIVLAVLVMTAESHNGVNHGPAKSPSSHDPKAHAPAPSAATFSAYP
QLIATALVGVISYGEGLGSVLCRIVSLDPHDGFSHASSSPLKKDTEEKMAQMVAMPVA
HSLSLICSWTKSNPLSRNTLALPPSNAPSKQSLSIRCARVGGVEIPSNKRIEYSLQYIHGI
GRTRARQILVDLQMENKITKDMAEEELIVLRDEVSKYMIEGDLRRFNALAIKRLKEIQ
CYRGVRHIQGLPCRGQRTKNNCRTLKGKKIAIAGKKKVSK.

>Gene0494890.1

MAYAAMKSTKAGLEEPLEQIHKIRITLSSKNVKNLEKVCADLVRGAKDKRLRTKGPF
TFVILTGTNTWDRFELRVHKRVIDLFSSPDVVKQITSITIELGVEVEVTIADS.

>Gene0101750.1

MLRSLLLRRSSNARSLRTPTSPFPPLRTLCTSGQTLTPPPPPPPPPISSSASEKEFRKYAGY
AALALFSGAATYFSFPFPENAKHKKAQIFRYAPLPEDLHTVSNWSGTHEVQTRNFNQP
ETLADLEALVKEAHEKKNRIRPVGSGLSPNGIGLSRSGMVNLALMDKVLEVDKEKKR

VRVQAGIRVQQLVDAIQEYGLTLQNFASIREQQIGGIIQVGAHGTGARLPPIDEQVIGM
KLVTPAKGTIQLSKDKDPELFHLARCGLGGLGVVAEVILQCVERQELVEHTYVSTLEE
IKKNHKKLLSTNKHVKYLYIPYTDTVVVVTCNPVSKWSGAPKDKPKYTTDEALKHV
RDLYRESIVKYRVQDSSKKTPDSREPDINELSFTELRDKLIALDPLNDVHVGKVNQAE
AEFWKKSEGYRVGWSDEILGFDCGGQQWVSETCFPAGTLAKPSMKDLEYIEQLKELI
EKEAIPAPSPIEQRWTGRSKSPMSPAFSTAEDDIFSWVGIIMYLPTADPRQRKDITDEFF
HYRHLTQAKLWDQYSAYEHWAKIEIPKDKEELEALQERLRKRFPVDAYNKARRELDP
NRILSNNMVEKLFPVSKTA.
>Gene0102170.1
MATSLSVSRLLSSSAISVAKPLLSPTSAFTAPISFSRSLAPNLSLKSPTRRTSISAARSFSA
TTVSASISVGDKLPDSTLSYLDPATNDVKTVTVSSLTAGKKTILFAVPGAFTPTCSQKH
VPGFVSKAGELRSKGVDVIACVSVNDAFVMEAWRKDLGISDEVMLLSDGNGEFTGK
LGVELDLRDKPVGLGVRSRRYAILAEDGVVKVLNLEEGGAFTNSSAEDMLKAL.
>Gene0103240.1
MLATVFLGFVLAKMTKNYPAVSEEYQKAIEKCKRKLRGLIAEKNCAPIMVRLAWHSA
GTFDCASRTGGPFGTMRFDDELAHGANNGLHIALRLLEPIREQFPTISHADFHQLAGV
VAVEVTGGPEIPFHPGREDKPQPPPEGRLPDATKGCDHLRQVFLKQMGLTDQDIVALS
GAHTLGRCHKDRSGFEGAWTSNPLIFDNSYFKELLSGEKEGLLQLPSDKALLDDPVF
RPLVEKYAADEEAFFADYAEAHLKLSELGFADA.
>Gene0104950.1
MTENADLLEWPKKDKRRFLHVVYRVGDLDRTIQFYTECFGMKLLRKRDVPEEKYSN
AFLGFGPETSNFVVELTYNYGVSSYDIGTGFGHFAISTQDVSKMVEAVRAKGGNVTR
EPGPVKGGGSVIAFVKDPDGYMFELIQRGPTPEPLCQVMLRVGDLDRAIKFYEKALG
MRLLRRIERPEYKYTIGMMGYAEEYESIVLELTYNYGVTEYTKGNAYAQIAIGTDDVY
KSAEVVKIANQELGGKITREAGPLPGLGTKIVSFLDPDGWKTVLVDNEDFLKELE.
>Gene0108910.1
MALRMWASSTANALKLSSSTSKSHLLPAFSISRCFSSVLEGLKYATSHEWVKHDGSVA
TIGITDHAQDHLGEVVFVELPEEKSSVSKEKNFGAVESVKATSEILSPISGEVIEVNTKL
TDSPGLINSSPYEDGWMIKVKPSNPAELETLMGSKEYTKFCEEEDAAH.
>Gene0109040.1

MASFKVMHSPTSTNSDPSRRIPSSSIRPRRDPNARILGGYDGYSPAAMTVEGILHETFA
SDPPATDSSLLMDASPTATVSGGTDTRGKSVDEVWRGMKEEEEMMTLEDFLAKATG
EDGGGESDDVDVKIPPAESYGFDHHNPFQMIDKVEGSIVAFGNGVDVYGGGARGKR
ARVMMEPLDKAAAQRQRRMIKNRESAARSRERKQAYQVELETLAAKLEEENEKLSV
EIEEKRKERYQKLMEFVIPVVERPKQEPRFLRRIRSLEW.

>Gene0135480.1

MWRCVSRSLRAPSSRTSLSGSRFSRFLSTGSQTGDYTIVDHTYDAVVVGAGGAGLRA
AIGLSEHGFNTACITKLFPTRSHTVAAQGGINAALGNMSEDDWRWHMYDTVKGSDW
LGDQDAIQYMCREAPKAVIELENYGLPFSRTEEGKIYQRAFGGQSLDFGKGGQAYRC
ACAADRTGHALLHTLYGQAMKHNTQFFVEYFALDLLMASDGTCQGVIALNMEDGT
LHRFRSAQTILATGGYGRAYFSATSAHTCTGDGNAMVARAGLPLQDLEFVQFHPTGIY
GAGCLITEGSRGEGGILRNSEGERFMERYAPTAKDLASRDVVSRSMTMEIREGRGVGP
HKDHIYLHLNHLPPEVLKERLPGISETAAIFAGVDVTKEPIPVLPTVHYNMGGIPTNYH
GEVVTIKGDDPDAVVPGLMAAGEAACASVHGANRLGANSLLDIVVFGRACANRVAE
ISKPGEKQRPLEENAGKKTIEWLNKLRHSSGSLPTSSIRLNMQRIMQNNAAVFRTQET
LEEGCQLIDKAWESFEDVQVKDRSLIWNSDLIETIELENLLINASITMHSAEARKESRG
AHAREDFTKREDGEWMKHTLGYWEDEKVRLEYRPVHMDTLDDEIDTFPPKARVY.

>Gene0554550.1

MAPKAAEKKPAEKKPAEKAPAEKKPKAGKKLPKDPSAVAGDKKKKRSKKSVETYKI
YIFKVLKQVHPDIGISSKAMGIMNSFINDIFEKLAGESSKLARYNKKPTITSREIQTAVR
LVLPGELAKHAVSEGTKAVTKFTSS.

>Gene0112020.1

MEHNGNIFRRSEVSGLTIFTCYPDSFSGSKRDTKYPSTNELLSQYYSLVDEKSFRPRTS
TSLSKRVAWVDPPHNNFTF.

>Gene0113410.1

MAMTLAASSSVVVMIPRVSTVSYLPPLPPRSFGRSSFTVPLKLVSGNGLQKVELMKTR
ASSSDETSASIDTNELFNDLKEKWDGLENKTTVVIYGGGAIVAVWLSSILVGAINSVPL
LPKVMELVGLGYTGWFVYRYLLFKSSRKELAEDIDSLKKKIAGTE.

>Gene0117160.1

MAGSATQFDDARQFDQALSEILEGQDEFFTSYDEVYESFDGMGLQENLLRGIYAYGF

EKPSAIQQRGIVPFCKGLDVIQQAQSGTGKTATFCSGVLQQLDVTLVQCQALVLAPTR
ELAQQIEKVMRALGDYIGVKVHACVGGTSVLDDKRILQAGVHVVVGTPGRVFDML
QRQSLRSDCIKMFVLDEADEMLSRGFKDQIYEIFQLLPPKIQVGVFSATMPPEALEITR
KFMSKPVRILVKRDELTLEGIRQFYVDVDKEEWKFETLCDLYETLAITQSVIFVNTRR
KVDWLTEKMRSRDHTVSATHGDMDQNTRDIIMREFRSGSSRVLITTDLLARGIDVQQ
VSLVINFDLPTQPANYLHRIGRSGRFGRKGAAINFVTRDDEKMLADIQKFYNMVVEE
LPSNLADLL.

>Gene0542010.1

MASHIVGYPRMGPKRELKFALESFWDKKSTAEDLQKVSADIRSGIWKQMSEAGTKYI
PSNTFAHYDQVLDTTAMLGAVPPRYGYTGGEIGLDVYFSMARGNASVPAMEMTKWF
DTNYHYIVPELGPEVNFSYASHKAVNEYKEAKALGVDTVPVLVGPVSYLLLSKAAKG
VEKSFDLLSLLPKILPVYKEVITELKAAGATWIQLDEPVLVMDLEGHKLQAFTGAYAE
LESTLSGLNVLVETYFADIPAEAYKTLTSLKGVTAFGFDLVRGTKTLDLVKAGFPEGKY
LFAGVVDGRNIWANDFAASLSTLEALEGVVGKDKLVVSTSCSLLHTAVDLVNETKLD
DEIKSWMAFAAQKVVEVNALAKALAGQKDEALFSANAAALASRRSSPRVTNEGVQ
KAAAALKGSDHRRATNVSARLDAQQKKLNLPILPTTTIGSFPQTVELRRVRREYKAK
KVSEEDYVKAMKEEIKKVVDLQEELDIDVLVHGEPERNDMVEYFGEQLSGFAFTANG
WVQSYGSRCVKPPVIYGDVSRPKAMTVFWSAMAQSMTSRPMKGMLTGPVTILNWS
FVRNDQPRHETCYQIALAIKDEVEDLEKGGIGVIQIDEAALREGLPLRKSEHAFYLDW
AVHSFRITNCGVQDSTQIHTHMCYSHFNDIIHSIIDMDADVITIENSRSDEKLLSVFREG
VKYGAGIGPGVYDIHSPRIPSSEEIADRVNKMLAVLEQNILWVNPDCGLKTRKYTEVK
PALKNMVDAAKLIRSQLASAK.

>Gene0330690.1

MVYASRIISHSKKLKHVSTLLRREHAVAVRGFSNTTQPSLTGREDLLKTRLNYSPAERI
SKCSASNVPMPSGISAMRTKPMTGPTFFREYISSQIRSVRGFSSGSDLPPHQEIGMPSLS
PTMTEGNIARWLKKEGDKVAPGEVLCEVETDKATVEMECMEEGYLAKIVKEEGAKE
IQVGEVIAITVEEEEDIQKFKDYTPSSGTPPAAPEAKPAPSPPKEEKVEKPASAPEAKTS
KPTSSPSEDRIFASPLAKKLAEDSNVPLSSIRGTGPEGRIVKADVEEFLASRGKETTAKP
SKPTDSKAPALDYVDIPHTQIRKVTASRLAFSKQTIPHYYLTVDTCVDKLMGLRSQLN
SFQEESGGKRISVNDLVIKAAALALRKVPQCNSSWTDEYIRQFSNVNINVAVQTENGL

YVPVVKDADKKGLSKIGEEVRFLAQKAKENSLKPEDYEGGTFTVSNLGGPFGIKQFC
AVINPPQAAILAIGSAEKRVVAGSGPDQFNVASYMSVTLSCDHRVIDGAIGAEWLKAF
KGYIETPESMLL.

>Gene0118450.1

MAYASRIIIHSNKLKHVSTLLRREHSVAARGFSNSSTHSSLTAREDMLKTRPPVERISKC
CATTVPRPSGISTMSTKPMTGPSFFREYISSQMRSVRGFSSGSDLPPHQEIGMPSLSPTM
TEACTSLNFLTTIHGNIAKWMKKEGDKVAPGEVLCEVETDKATVEMECMEEGYLAKI
LKEEGTKAIQVGEVIAITVEEEEDIQKFKDYTPSSGGSPAAPEAKPKPASAPEAETSKPS
PAPSEDRIFASPLAKKLAEDNNVPLSSIKATGPEGRIVKADVEEFLASRGKETTAKPSKP
TDSKVPALDYVDIPHTQIRKVTASRLAFSKRTIPHYYLTVDTCVDKMMGLRSQLNSFQ
EANGGKRISVNDLVIKAAALALRKVPQCNSSWTDEYIRQFSNVNINVAVQTENGLYVP
VVKNADKKGLSTIGEEVRFLAQKAKENSLKPEDYEGGTFTVSNLGGPFGIKQFCAVIN
PPQAAILAIGSAEKRVVAGGGPDQFNVASYMSVTLSCDHRVIDGAIGAEWLKAFKGYI
ETPESMLL.

>Gene0119370.1

MASTFMSSSSVLTPTPFLGQTKGSTFNPLRDAVSLGSPKYTMGNDLWYGPDRVKYLG
PFSVQTPSYLTGEFPGDYGWDTAGLSADPEAFAKNRALEVIHGRWAMLGALGCITPE
VLQKWVRVDFKEPVWFKAGSQIFSEGGLDYLGNPNLVHAQSILAVLGFQVILMGLVE
GFRINGLDGVGEGNDLYPGGQYFDPLGLADDPVTFAELKVKEIKNGRLAMFSMFGFF
VQAIVTGKGPLENLLDHLDNPVANNAWAFATKFVPGA.

>Gene0120510.1

MSWDDGKHAKVKKVQLTFDDVIRSIEVEYEGTNLKSQRRGTVGTRSDGFTLSTDEYI
TSVSGYYKTTFSGDHITALTFKTNKKTYGPYGNKTQNYFSADAPKDSQIAGFLGTSGN
ALSSLDVHFAPIPTPGSIKPQPGGSGTGGGGSKPGGSGNESGGGGGGSKPGGSGNESG
GGSSKPGGSGNESGGGGGGGSKPGGSGNESGGGGGSGNETGNDGPGKMGPLGGDK
GNVFDDVGFEGVKKITVGADQYSVTYIKIEYIKDGQVVVREHGTVRGELKEFSVDYP
NDNITAVSGTYKHVYTYDTTLITSLYFTTSKGFTSPLFGIDSEKKGTEFEFKGENGGKL
LGFHGRGGNAIDAIGAYFDTGSQGGKGGGGGSQTDVPGKKGPLGGDKGEEFNDVGF
EGVKKITVGADQYSVTYIKIEYVKDGKVEIREHGTSRGELQEFSVDYPNDKITAVSGT
YKHIFTYDTTLITSLYFTTSKGFTSPLFGINSEKKGTEFEFKGENGGKLIGLHGRGGNAI

DAIGAYFDTGSQGGDGGDVPSKDGPNTDVPGKKGPLGGDKGEPFDDVGFEGVKKIT
VGVDNLSITYIKIEYVKDGKVEVREHGTARGKLQEFSVDYPNDSITEVAGTYKHNYT
YDTTLITSLYFTTSKGFTSPLFGIDSEKKGTEFEFKDENGGKLIGFHGRGGNAIDAIGAY
FDTGSKPGGNGDNGSGSNSGSSPQKLDAQGGKGGNQWDDSGDHDGVTKIHVAYSR
VIEQIKFEYVKNGETKEGPAHGVKGGARTIIGTFEISHPNEYLLSVKGWSDSSNKIVGI
QFTTNTKTSDYYGFEKYPGDEGTDVLLEVKDKKIVGFHGFADNQLNSLGAYFAPISST
PLKPSKKLQGVGGDEGASWDDGAFDGVKKIQIGQNNDGVSFVAVEYQNGSQKVVG
DGHGKQSPLGVETFELADGEYITSVGVYYDKIHAEGRGVTVVTSLIFKTNKQISQPFG
MTGGEYVELKEEGNKIVGFHGKASDWVHQIGVYVAPVTK.
>Gene0331610.1
MMLRAVIRRASTRGGGSSASGLGKSLQSSRVAASTQSFHSLSATQTLVPRGTDARSFH
HRSCPGCSECSSRTVFSSFQGTTLQKWVRPYSSDSGDVVEAVVPHMGESITDGTLANF
LKKPGDRVEADEAIAQIETDKVTIDIASPASGVIQEFLVKEGDTVEPGNKVAIISTSADA
VSHVAPSEKVAEKPAAKPSPPAEAPKVESTKVAEKPKAPSPPPPTKQSAKEPQLPPKDR
ERRVPMTRLRKRVATRLKDSQNTFALLTTFNEVDMTNLMKLRSQYKDAFFEKHGVK
LGLMSGFIKAAVSALQAQPVVNAVIDGDDIIYRDYVDISIAVGTSKGLVVPVIRGADQ
MNFADIEKTINSLAKKANEGTISIDEMAGGSFTVSNGGVYGSLISTPIINPPQSAILGMH
SIVQRPMVVGGSVVPRPMMYVALTYDHRLIDGREAVYFLRRIKDVVEDPQRLLLDI.
>Gene0157750.1
MAEEKKVAPTGVWTAVKPFVNGGASGMLATCVIQPIDMIKVRIQLGQGSAVSVTKN
MLKNDGIGAFYKGLSAGLLRQATYTTARLGSFKMLTAKAIEANDGKPLPLYQKALCG
LTAGAIGACVGSPADLALIRMQADNTLPLAQRRNYTNAFHALYRISADEGVLALWKG
CGPTVVRAMALNMGMLASYDQSAEYMRDNLGLGETSTVVGASAVSGFCAAACSLP
FDYVKTQIQKMQPDAQGKYPYTGSLDCAMQTLKSGGPLKFYTGFPVYCVRIAPHVM
MTWIFLNQITKFQKTIGL.
>Gene0122810.1
MEKAIERQRVLLEHLRPSSSSSSHSFEGSLSASACLAGDSAAYQRTSLYGDDVVIVAAH
RTALCKSKRGNFKDTYPDDLLAPVLRALIEKTNLDPSEVGDIVVGTVLAPGSQRASEC
RMSAFYAGFPETVAVRTVNRQCSSGLQAVADVAAAIKAGFYDIGIGAGLESMTTNPMA
WEGSVNPAVKKFEQAQNCLLPMGVTSENVAHRFGVSRQEQDQAAVDSHRKAAAATA

AGKFKDEIIPVKTKLVDPKTGDETPITVSVDDGIRASTTLATLGKLKPVFKKDGTTTAG
NSSQVSDGAGAVLLMRRSVATQKDFPFLVTFAAVGVDPAIMGVGPAVAIPAAVKAAGL
ELDDIDLFEINEAFASQFVYCRNKLGLDAEKINVNGGAMAIGHPLGATGARCVATLLH
EMKRRGKDCRFGVVSMCIGTGMGAAAVFERGDGVDELRNASKVEAQGFLSKDAR.

>Gene0380650.1

MASLASSTTLISSSTVLLPSKPSPFSPAASFLRTLPSTAASPSSLRSGFSSIGSLTCIPSSSR
RSFAVKAQADDLPLVGNKAPDFEAEAVFDQEFIKVKLSEYIGKKYVILFFYPLDFTFVC
PTEITAFSDRYEEFEKLNTEVLGVSVDSVFSHLAWVQTERKSGGLGDLNYPLVSDITKS
ISKSFGVLIPDQGIALRGLFIIDKEGVIQHSTINNLGIGRSVDETMRTLQALQYVQENPD
EVCPAGWKPGEKSMKPDPKLSKEYFSAI.

>Gene0123770.1

MPCLNISTNVSLDGVDTSSILSEATSSVAKIIGKPENYVMIVLKGSVPMAFGGTEDPAA
YGELVSIGGLNPDVNKQLSAAVSAILETKLSVPKSRFFLKFYDTKATLFPLSLFDAL.

>Gene0199280.1

MQIFVKTLTGKTITLEVESSDTIDNVKAKIQDKEGIPPDQQRLIFAGKQLEDGRTLADY
NIQKESTLHLVLRLRGGAKKRKKKTYTKPKKIKHKHKKVKLAVLQFYKVDGSGKVQ
RLRKECPNATCGAGTFMASHFDRHYCGKCGLTYVYQKEGAEA.

>Gene0129600.1

MEKNIKFPVIDLSKLNGEERDQTMALIDDACQNWGFFELLNHGIPYDLMDNIERMTK
EHYKKFMEQKFKEMLRSKGLDTLETEVEDIDWESTFFLHHLPQTNLYDIPNMSDEYR
TAMKDFGKRLENLAEELLDLLCENLGLEKGYLKKVFRGTKGPTFGTKVSNYPPCPNP
EMIKGLRAHTDAGGLILLFQDDKVSGLQLLKDGDWVDVPPLKHSIVINLGDQLEVIT
NGKYKSVMHRVMTQKEGNRMSIASFYNPGSDAEISPAQSLVDKESEYPSFVFDDYMK
LYSGVKFQPKEPRFEAMKNAAVTTDVNPVATVETF.

>Gene0130370.1

MAIKNLLTSALRSQRRLALNQATRASSSISALDSATHSPPPPATPILMPYDHAAEITKEK
LKRLENPDQRFLKYASPHPILASHNHILSSPETRVTTLPNGLRVATESNLSAKTATVGV
WIDAGSRFESDETNGTAHFLEHMIFKGTERRTVRALEEEIEDIGGHLNAYTSREQTTYY
AKVMESDVNQALDVLADILQNSKFEEQRINRERDVILREMQEVEGQTDEVVLDHLH
ATAFQYTPLGRTILGPAQNVKSITREDLQNYIKTHYTASRMVIAAAGAVKHEEVVEQV

KKLFNKLSSDPTSTTQLVAKEPASFTGSEVRMIDDDLPLAQFAVAFEGASWTDPDSVAL
MVMQTMLGSWNKNVGGGKHTGSALIQRVAINEIAESIMAFNTNYKDTGLFGVYAVA
KADCLDDLSYAIMHEVTKLAFRVSDDDVTRARNQLKSSLLLHMDGTSPIAEDIGRQL
LTYGRRIPTAELFARIDAVDASTVKRVANKFVYDKDIAISAIGPIQDLPDYNKFRRRTYF
NRY.
>Gene0395090.1
MACTNLTTMWVSSKLSISDSSSLSFRSILNPLPLPNHNSSPSRSSSVSPIQSSLRELRDRI
DSVKNTQKITEAMKLVAAAKVRRAQEAVVNGRPFSETLVEVLYNINEQLQTDDIDVPL
TKIRPVKKVALVVVTGDRGLCGGFNNFIIKKAEARIKELQGLGLDYTVISVGKKGNSY
FLRRPYIPVDKYLEAGTLPTAKEAQAVADDVFSLFISEEVDKVELLYTKFVSLVKSEPVI
HTLLPLSPKGEICDINGNCVDAAEDELFRLTTKEGKLTVERETFRTPTADFSPILQFEQD
PVQILDALLPLYLNSQILRALQESLASELAARMSAMSSASDNASDLKKSLSMVYNRK
RQAKITGEILEIVAGANAQA.
>Gene0256570.1
MLALVRTVIHLGSESAKMFSAQNKIHKDKGVAPTDFEQEVAQAFFDLENTNQELKSD
LKDLYINQAVQMDCSGNRKAIVIYVPFRLRKAFRKIHPRLVRELEKKFSGKDVIFVATR
RIMRPPKKGSAVQRPRNRTLTSVHEAMLEDVAYPAEIVGKRTRYRVDGTKIMKVFLEP
KERNNTEYKLETMVGVYRKLTGRDVVFEYPVQEL.
>Gene0134200.1
MKITHFVLSSLETFRVFDSLLIAAKLSSLREPRFLQSSRMDSQIKHAVVVKVMGRTGSR
GQVTQVRVKFTDSDRYIMRNVKGPVREGDILTLLESEREARRLR.
>Gene0499720.1
MKFNVANPTTGCQKKLEIDDDQKLRAFYDKRLSQEVSGDALGEEFKGYVFKIMGGC
DKQGFPMKQGVLTPGRVRLLLHRGTPCFRGHGRRTGERRRKSVRGCIVSQDLSVLNL
VIVKKGEKDLPGLTDTEKPRMRGPKRASKIRKLFNLGKDDDVRKYVNTYRRKFTNK
KGKEVSKAPKIQRLVTPLTLQRKRARIADKKKRIAKANADAADYQKLLASRLKEQRD
RRSESLAKKRSRLSSAAAKPVAA.
>Gene0247260.1
MTQGQLVGKGGDMVNGEMARKRLKISVPHFDNSDLIKSYDMTLVGRCMNPEAQKV
DSLLVMLPKFWKVEERVTGADLGMGKFQFHFEREEDIKAVLEMQPYHFDYWMLSLA

RWQPRMSKNFPSEIPFWIKVEGVPLELWSTETFQSIGDAIGVTTDVDLDFGKMRVVLD
SAKELCFETEVDFKGGEFYEDEEVLVMLKYDKLFGFCKRCFNLCHDEDHCPLNPRSP
SKKKETKEVEERKEERARSYRGVVINGDGGKQETSKDHREYYGKGKGKMYEEPEA
KWVKVPERGSKRSRARNPRWEQSRYPMQDDRNRYHRGVRREQEGPKEEGEITVTDR
RQRNLHKVENIGEKAVPVEPSVTAMDLEGEVGDRAEIENLVTGLEQAMDLVGAGKG
VADSEPLEFDGTGTLADHVEELLGGDDEFQALTDGEVEETNKLDEVPRGGAEGEVEI
GGNGGPYDQMGEEARKKGARKVLFKKPPGISVGTSKMRLVQAVLSPRKTGVSKSTK
RQGGGEGAKHTEEKGPSHPKNPKKPDV.

>Gene0135540.1

MTMAANFARRLIGSRTTVNSTSTAATSAARAFCSSTTPITATLFPGDGIGPEIAESVKKV
FTTAGVPINWEEHYVSTEIDPRTQSFLTWESLESVRRNKVGLKGPMATPIGKGHRSLN
LTLRKELNLYANVRPCYSLPGYKTRYDDVNLITIRENTEGEYSGLEHQVVRGVVESLK
IITRQASLRVAEYAFHYAKTHGRERVSAIHKANIMQKTDGLFLKCCREVAEKYPEITYE
EVVIDNCCMMLVKNPALFDVLVMPNLYGDIISDLCAGLVGGLGLTPSCNIGEDGVALA
EAVHGSAPDIAGKNLANPTALLLSGVMMLRHLKMNEQAEQIHSAIINTIAEGKYRTAD
LGGSSTTTDFTKAICDHL.

>Gene0138550.1

MATTTAAAASGIFGIRIQDPSSGAGRVQAKFNFSFGKKKPAPPPKKTKQVQNDGDRLV
WFPGANPPEWLDGSMIGDRGFDPFGLGKPAEYLQYDFDGLDQNLAKNVAGELLGVR
QDSKEISPTPFQPYTEVFGIERFRECELIHGRWAMLGVLGALAVEGLTGIAWQDAGKV
ELVEGSSYLGQPLPFSLTTLIWIEVLVVGYIEFQRNAELDPEKRIYPGGYFDPLGLAADP
EKLDTLKLAEIKHSRLAMIAFLIFGLQAAFTGKGPISFLATFSS.

>Gene0508330.1

MPPKLDPSQIVDVYVRVTGGEVGAASSLAPKIGPLGLAPKKIGEDIAKETAKEWKGLR
VTVKLTVQNRQAKVTVVPSAAALVIKALKEPERDRKKVKNIKHNGNISFDDVIEIARI
MRPRSIAKELSGTVREILGTCVSVGCTVDGKDPKDIQQEIQEGEVEIPEN.

>Gene0151250.1

MAMSILRREGRRLLRPIAAIRSPLASSDQEEGLLGVRSISTQVVRNRMKSVKNIQKITK
AMKMVAASKLRAVQGRAENSRGLWQPFTALLGDNPSIDVKKSVVVTLSSDKGLCGG
INSTVVKVSRALYKLNAGPEKEVKFVIVGEKAKAIMFRDSKNDISLTVTELNKNPLNY

AQVSVLADDILKNVEFDALRIVYNKFHSVVAFLPTVATVLSPEIIEKESEVGGKLGELD
SYEIEGGETKGEILQNLAEFQFSCVMFNAVLENACSEMGARMSAMDSSSRNAGEML
DRLTLTYNRTRQASITTELIEIISGASALEAAK.

>Gene0163810.1

MAASLQSATTFLQSAKISTAPSRGSAHLRSTQTVGKSFGLETSSARLTCSFQSDFKDFA
GKCSDAVKIAGFALATSALVVSGASAEGAPKRLTYDEIQSKTYMEVKGTGTANQCPTI
DGGSETFSFKPGKYAGKKFCFEPTSFTVKAESVSKNAPPDFQNTKLMTRLTYTLDEIE
GPFEVSSDGSVNFKEEDGIDYAAVTVQLPGGERVPFLFTVKQLDASGKPDNFTGKFLV
PSYRGSSFLDPKGRGGSTGYDNAVALPAGGRGDEEELSKENVKNTAASVGEITLKVT
KSKPETGEVIGVFESLQPSDTDLGAKVPKDVKIQGVWYGQLE.

>Gene0149610.1

MDIYSSSFHQAHNKVSLLRRQPSSPVNSVSVIGFSLPRITSPSLAKYRRKGSSTGFVRA
CVAVEEKKRTAIIRIGTRGSPLALAQAYETRANLQAKHPELTEDGAIHIEIIKTTGDKILS
QPLADIGGKGLFTKEIDEALINGHIDIAVHSMKDVPTYLPEKTILPCNLVREDVRDAFIC
LTAASLAELPAGSVVGTASLRRKSQILHKYPSLAVEENFRGNVQTRLSKLQGGKVHAT
LLALAGLKRLSMTENVASVLSLDEMLPAVAQGAIGIACRTEDDKMASYLASLNHEET
RLAVACERAFLETLDGSCRTPIAGYAAKDEEGNCFFRGLVASPDGTRVLETSRKGPYV
FEDMVKMGKDAGQELLSLAGPGFFGN.

>Gene0221480.1

MAPTLQGEWIKVQQKGGEGPGARSSHGIAVVGDKLYSFGGERTPNISIDKHLYVFDFN
THTWSIAPANGQAPNVQALGTRMVAVGTMLYLFGGRDEKKQFDDFYSYDTVKQEW
KFITKLDEEGGPEARTYHSMASDENHVYVFGGVSKGGTNKTPFRFRTIEAYNIADGK
WAQLPDPGEQFPRFERRGGAGFIVVEGKIWVVYGFATSPDPNGKNDYESDLVHYFDP
ATQKWTEVETKGEKPSPRSVFAHAAVGKYIIIFGGEVWPDPNGHLGPGTLTNEGYALN
TETLVWEKFGGGAEPGELGWPAYTTATVYGKQGLLMHGGKRPTNKRTDEMYFYAVH
SA.

>Gene0557730.1

MGKEKFHINIVVIGHVDSGKSTTTGHLIYKLGGIDKRVIERFEKEAAEMNKRSFKYAW
FETTKYYCTVIDAPGHRDFIKNMITGTSQADCAVLIIDSTTGGFEAGISKDGQTREHAL
LAFTLGVKQMICCCNKMDATTPKYSKARYDEIIKEVSSYLKKVGYNPDKIPFVPISGF

EGDNMIERSTNLDWYKGPTLLEALDQINEPKRPSDKPLRLPLQDVYKIGGIGTVPVGR
VETGMLKPGMVVTFAPSGLTTEVKSVEMHHESLVEALPGDNVGFNVKNVAVKDLKR
GYVASNSKDDPAKGAANFTSQVIIMNHPGQIGNGYAPVLDCHTSHIAVKFSEILTKIDR
RSGKEIEKEPKFLKNGDAGMVKMTPTKPMVVETFSEYPPLGRFAVRDMRQTVAVGVI
KSVDKKDPTGAKVTKAAVKKGAK.

>Gene0505150.1

MAVSTIYSTQALNSTHFFTSSSSSKQVFFYRRQNNRRFNTIITCAAQQTVVIGLAADSG
CGKSTFMRRLTSVFGGAAEPPKGGNPDSNTLISDMTTVICLDDYHSLDRTGRKEKGV
TALDPRANDFDLMYEQVKALKSGIAVEKPIYNHVTGLLDAPELIQPPKILVIEGLHPMF
DERVRDLLDFSIYLDISNEVKFAWKIQRDMAERGHSLESIKASIEARKPDFEAFIDPQK
QYADAVIEVLPTQLIPDDNEGKVLRVRLIMKEGVKYFSPVYLFDEGSTISWIPCGRKLT
CSYPGIKFNYAPDSYFDHEVSVVEMDGQFDRLDELIYVESHLSNLSTKFYGEVTQQM
LKHADFPGSNNGTGLFQTIVGLKIRDLYEQLIANKATAPAEAAKA.

>Gene0155430.1

MASVALLRALRRRELHTASVSAFKSVSTNGKTSLVGHFARPFCSRPVGNDVIGIDLGT
TNSCVAVMEGKTPRVIENAEGTRTTPSVVAINQKGELLVGTPAKRQAVTNPTNTIFGSK
RLIGRRFDDSQTQKEMKMVPYKIVKAPNGDAWVEANGQKFSPSQIGANVLTKMKET
AEAYLGKSITKAVVTVPAYFNDAQRQATKDAGKIAGLDVQRIINEPTAAALSYGMNN
KEGVIAVFDLGGGTFDVSILEISSGVFEVKATNGDTFLGGEDFDNTLLEYLVSEFKRSD
NIDLTKDKLALQRLREAAEKAKIELSSTSQTEINLPFITADASGAKHLNITLTRSKFEAL
VGKLIERTRCPCQNCLKDAGVTVKEVDEVLLVGGMTRVPKVQEIVAEIFGKSPCKGV
NPDEAVAMGAAIQGGILRGDVKELLLLDVTPLSLGIETLGGIFTRLINRNTTIPTKKSQV
FSTAADNQMQVGIKVLQGEREMAADNKSLGEFDLVGIPPAPRGMPQIEVTFDIDANGI
VTVSAKDKATGKEQQITIRSSGGLSDDEINRMVKEAELNSHKDQEKKQLIDLRNTADT
TIYSVEKSLSEYREKIPAEIASEIETAVSDLRTAMAGEEIEDIKAKLEAANKAVSKIGEH
MSKGSGSSGSSGGEGSSGTDQQTPEAEFEEASGSKK.

>Gene0545850.1

MATQGQVITCKAAVAYEPNKPLVIEDVQVAPPQAGEVRIKILFTALCHTDAYTWSGKD
PEGLFPCILGHEAAGIVESVGEGVTEVQPGDHVIPCYQAECRECKFCKSGKTNLCGKV
RSATGVGVMMNDRKSRFSVNGKPIYHFMGTSTFSQYTVVHDVSVAKIDPQAPLEKV

CLLGCGVPTGLGAVWNTAKVEPGSNVAIFGLGTVGLAVAEGAKTAGATRIIGIDIDSK
KYETAKKFGVNEFVNPKDHQKPIQEVIVDLTDGGVDYSFECIGNVSVMRAALECCHK
GWGTSVIVGVAASGQEISTRPFQLVTGRVWKGTAFGGFKSRTQVPWLVEKYMNKEIK
VDEYITHNMTLGEINKAFDLLHEGTCLRCVLSTSE.
>Gene0157630.1
MKTILSSETMDIPESVSIKVHAKVIEVEGPRGKLVRDFKHLNLDFQLIKDAETGKRKL
KIDSWFGSRKSSASIRTALSHVDNLITGVTRGFRYKMRFVYAHFPINASIGGDSKSIEIR
NFLGEKKVRKVEMLDGVTIVRSEKVKDEIVLDGNDIELVSRSCALINQKCHVKKKDI
RKFLDGIYVSEKSKIVEEE.
>Gene0453970.1
MARIKVHELRDKSKSDLQNQLQDLKAELALLRVAKVTGGAPNKLSKIKVVRKSIAQV
LTVTSQKQKSALREAYKNKKFIPLDLRPKKTRAIRRRLTKHQLSLKTEREKKKEMYFP
IRKYAIKV.
>Gene0161160.1
MASSSSFFTFSSQTTLHSHLHRNTFLTKTQFPVPTKSSESNFFGLRLSPSTSRSPLSPCSF
KSSIFAKVNKGQSAPDFTLKDQNGKPVSLKKYKGKPVVVYFYPADETPGCTKQACAF
RDSYEKFKKAGAEVIGISGDDSASHKAFASKYKLPYTLLSDEGNRVRKDWGVPGDLF
GALPGRQTYVLDKNGVVQLIYNNQFQPEKHIDETLKFLKAA.
>Gene0163880.1
MDPYKYRPSSAYNAPFYTTNGGAPVSNNISSLTIGERGPVLLEDYHLIEKVANFTRERI
PERVVHARGISAKGFFEVTHDISNLTCADFLRAPGVQTPVIVRFSTVVHERASPETMR
DIRGFAVKFYTREGNFDLVGNNTPVFFIRDGIQFPDVVHALKPNPKTNIQEYWRILDY
MSHLPESLLTWCWMFDDVGIPQDYRHMEGFGVHTYTLVSKSGKVLFVKFHWKPTC
GIKNLTDEEAKVVGGANHSHATKDLHDAIASGNYPEWKLFIQTMDPADEDKFDFDPL
DVTKIWPEDILPLQPVGRLVLNRTIDNFFNETEQLAFNPGLVVPGIYYSDDKLLQCRIF
AYGDTQRHRLGPNYLQLPVNAPKCAHHNNHHEGFMNFMHRDEEINYYPSKFDPVRC
AEKVPIPTKSYTGIRTKCIIKKENNFKQPGDRYRSWAPDRQDRFVKRWVEILSEPRLTH
EIRSIWISYWSQADRSLGQKLASRLNVRPSI.
>Gene0165540.1
MESFPIINLEKLNGEERGLTMEKIKDACENWGFFECVNHGIPHELLDRVEKMTKEHY

KKCMEDRFKESIKNRGLDSVRSEVNDVDWESTFYLKHLPASNISHVPDLDDDYRTLM
KEFAGKIEMLSEELLDLLCENLGLEKGYLKKVFYGSKSPTFGTKVSNYPPCPKPDLIK
GLRAHTDAGGIILLFQDDKVSGLQLLKDGEWVDVPPVKHSIVVNLGDQLEVITNGKY
KSVEHRVIAQTDGEGRMSIASFYNPGSDSVIFPAPELIGKENEKKDNYPKFVFEDYMK
LYSAVKFQAKEPRFEAMKAMETTVANNVGPLATA.

>Gene0282600.1

MEITNVTEYEAIAKEKLPKMVYDYYASGAEDQWTLKENRNAFARILFRPRILIDVSKI
DMTTTVLGFKISMPIMVAPTAMQKMAHPEGEYATARAASAAGTIMTLSSWATSSVEE
VASTGPGIRFFQLYVYKNRKVVEQLVRRAEKAGFKAIALTVDTPRLGRRESDIKNRFT
LPPNLTLKNFEGLDLGKMDEANDSGLASYVAGQIDRTLSWKDVQWLQTITSMPILVK
GVLTGEDARIAIQAGAAGIIVSNHGARQLDYVPATISALEEVVKATQGRVPVFLDGGV
RRGTDVFKALALGASGIFIGRPVVFSLAAEGEAGVRKVLQMLRDEFELTMALSGCRS
LSEITRNHIVTEWETPRHLPRL.

>Gene0393550.1

MEITNVTEYEAIAKEKLPKMVYDYYASGAEDQWTLQENRNAFARILFRPRILIDVSKI
DMTTTILGFKISMPIMVAPTAMQKMAHPEGEYATARAASAAGTIMTLSSWATSSVEEV
ASTGPGIRFFQLYVYKNRKVVEQLVRRAERAGFKAIALTVDTPRLGRRESDIKNRFTL
PPNLTLKNFEGLDLGKMDEANDSGLASYVAGQIDRTLSWKDVQWLQTITSMPILVKG
VITGEDARIAIQAGAAGIIVSNHGARQLDYVPATISALEEVVKATQGRIPVFLDGGVRR
GTDVFKALALGASGIFIGRPVVFSLAAEGEAGVRKVLQMLRDEFELTMALSGCRSLK
EITRNHITTEWDTPRPSARL.

>Gene0393290.1

MAISTPAACSSSSRLISPPSLRPAISTSTNLKTLNLSSSFLPSYSLSTLSASSTPSPHSPRRS
FTVRAARGKFERKKPHVNIGTIGHVDHGKTTLTAALTMALASMGNSVAKKYDEIDAA
PEERARGITINTATVEYETENRHYAHVDCPGHADYVKNMITGAAQMDGAILVVSGAD
GPMPQTKEHILLAKQVGVPDMVVFLNKEDQVDDAELLELVELEVRELLSSYEFNGD
DIPIISGSALLAVETLTENPNVKRGDNKWVDKIYELMDATELPFLLAVEDVFSITGRGT
VATGRVERGTVKVGETVDLVGLRETRNYTVTGVEMFQKILDEALAGDNVGLLLRGIQ
KADIQRGMVLAKPGSITPHTKFEAIVYVLKKEEGGRHSPFFAGYRPQFYMRTTDVTG
KVTKIMNDKDEESKMVMPGDRVKIVVELIVPVACEQGMRFAIREGGKTVGAGVIQSII

E.

>Gene0170470.1

MRGLVNKLVSRSLSVSGKWQHQQLRRLNIHEYQGAELMGKYGVNVPKGVAVSSLDE

VKNAIQQVFPNETELVVKSQILAGGRGLGTFKSGLKGGVHIVSRDQVPDIADAWTSA

RHQTNGSSRQSRQQGLCLKIILLVYLCEKLSLVNEMYFSIILDRKSAGPLIIACKKGGTS

IEDLAEKFPDMIIKVPIDVFAGITDEDAAKVVDGLAPKAADRKDSIEQVKKLYELFRK

TDCTMLEINPLAETSTNQLVAADAKLNFDDNAAFRQKDIFALRDPTQEDPREVAAAK

VDLNYIGLDGEIGCMVNGAGLAMATMDIIKLHGGTPANFLDVGGNASEHQVVEAFKI

LTSDDKVKAILVNIFGGIMKCDVIASGIVNAAKEVSLKVPVVVRLEGTNVEQGKRILK

ESGMKLITADDLDDAAEKAVKALAN.

>Gene0175390.1

MAAVGVESMRPETAMEETCNVKIAAAKQGEGLKQYYLQHIHELQRQLRQKTNNLN

RLEAQRNELNSRVRMLREELQLLQEPGSYVGEVVKVMGKNKVLVKVHPEGKYVVDI

DKSIDINKITPSTRVALRNDSYVLHLVLPSKVDPLVNLMKVEKVPDSTYDMIGGLDQQ

IKEIKEVIELPIKHPELFESLGIAQPKGVLLYGPPGTGKTLLARAVAHHTDCTFIRVSGSE

LVQKYIGEGSRMVRELFVMAREHAPSIIFMDEIDSIGSARMESGSGNGDSEVQRTMLE

LLNQLDGFEASNKIKVLMATNRIDILDQALLRPGRIDRKIEFPNPNEDSRCDILKIHSRK

MNLMRGIDLKKIAEKMNGASGAELKAVCTEAGMFALRERRVHVTQEDFEMAVAKV

MKKDTEKNMSLRKLWKRPRGTKAEKDLTVEKGALKIESDQKEGNLTVVRIASDLSE

NPCKNFPLLKTPSPSRRFSPSSQYHLPARSLTSSDSCDSENMALPNQQTVDYPSFKLVI

VGDGGTGKTTFVKRHLTGEFEKKYEPTIGVEVHPLDFFTNCGKIRFYCWDTAGQEKF

GGLRDGYYIHGQCAVIMFDVTARLTYKNVPTWHRDLCRVCENIPIVLCGNKVDVKNR

QVKAKQVTFHRKKNLQYYEISAKSNYNFEKPFLYLARKLAGDPNLHFVESPALAPPE

VHIDVAEQQKNEADLIAAAAQPLPDDDDDAFE.

>Gene0474130.1

MASVTSATVAIPSFTGLKSTISKPSAVVRIPMAATSSKLTVKSSLKDFGVAAVAAAASIA

LAGNAMAIDVLLGSGDGALAFVPNEFTIAKGEKIVFKNNAGFPHNVVFDEDEIPSGV

DASKISMDEQDLLNAAGETYEVALTEPGTYSFYCAPHQGAGMVGKVTVN.

>Gene0183410.1

MASSMLSSAAVATSPAQATMVAPFTGLKSSAAFPVTRKSNTDITSIASNGGRVSCMKV

WPPVGKKKFETLSYLPDLTDVELAKEVDYLLRNKWIPCVEFELEHGFVYREHGSTPG
YYDGRYWTMWKLPLFGCTDSSQVLKEVQECKTEYPNAFIRIIGFDNNRQVQCISFIAY
KPPSFTGA.

>Gene0548300.1

MAYSMLSSAAVVTSPAQATMVAPFTGLKSSSAFPVTRKANNDITSIVSNGGRVSCMKV
WPPVGKKKFETLSYLPDLTEVELGKEVDYLLRNKWIPCVEFELEHGFVYREHGSTPG
YYDGRYWTMWKLPLFGCTDSAQVLKEVQECKTEYPNAFIRIIGFDNNRQVQCISFIAY
KPPSFTGA.

>Gene0184540.1

MASHIVGYPRMGPKRELKFALESFWDGKTTAEDLKKVSADLRSDIWKQMSAAGIKYI
PSNTFAYYDQVLDTTAMLGAVPPRYGWNSGEIGFDVYFSMARGNASVPAMEMTKWF
DTNYFSYASHKAVNEYKEAKALGVETVPVLVGPVSYLLLSKLAKGVDKSFDLLSLLP
KILPIYKEVIAELKGAGATWIQFDEPLFVMDLEGHKLQAFSGAYAELESTLSGLNVLVE
TYFADVPAEAYKTLTSLKGVTAFGFDLIRGTKTIDLIKSSFPEGKYLFAGVVDGRNIWA
NDLAASLITLQSLEGVVGKDKLVVSTSCSLLHTAVDLVNETKLDAEIKSWLAFAAQKV
VEVDALAKALAGQTNQSFFSANAEALSSRRSSPRVTNQSVQKAAAALKGSDHRRAT
EVSARLDAQQKKLNLPILPTTTIGSFPQTVELRRVRREYKAKKISEEDYVKAIKEEIKK
VVDIQEELDIDVLVHGEPERNDMVEYFGEQLSGFAFTANGWVQSYGSRCVKPPIIYGD
VSRPKPMTVFWSSTAQSMTKRPMKGMLTGPVTILNWSFVRNDQPRHETCYQIALAIK
DEVEDLEKGGIGVIQIDEAALREGLPLRKAEHSFYLDWAVHSFRITNCGVQDSTQIHT
HMCYSHFNDIIHSIIDMDADVITIENSRSDEKLLSVFREGVKYGAGIGPGVYDIHSPRIP
STDEIADRVNKMLAVLEQNILWVNPDCGLKTRKYTEVKPALKNMVDAAKLIRSQLSS
AK.

>Gene0189130.1

MLFSQLLGHNTSRVLAEAKNHKRTERCLKAMAFLSKFGNILKQTNANGSLSSPSLFQ
AIRCMSSSKLFIGGMEYGMNEDSLREAFSKYGEVVETRVILDRETGRSRGFGFVTFTS
TEAASSAIQALDGQDLHGRIVKVNYAHDRTSGGGGYGGGGYGGGGYGSGGGGYGG
GGYGGGSGGYGGGGAGGYGGGGYGTSGGYGSSGSGYGEGSTASTGAVGGYNGSSG
YGEGSTANAGAAGGYNASSGYGNGNVYGSNNGGFAGENQVGGDNSQFSGENTQFG
VGHQSGGDAQVGGFEDDTDVAKRA.

>Gene0552070.1

MAASTMALSSPAFAGKAVKLSPSASEVLGSGRVTMRKTVAKPKGPSGSPWYGSERVK

YLGPFSGEPPSYLTGEFPGDYGWDTAGLSADPETFARNRELEVIHCRWAMLGALGCV

FPELLARNGVKFGEAVWFKAGSQIFSEGGLDYLGNPSLVHAQSILAIWATQVILMGAV

EGYRVAGEGPLGEAEDLLYPGGSFDPLGLATDPEAFAELKVKEIKNGRLAMFSMFGFF

VQAIVTGKGPLENLADHLADPVNNNAWAFATNFVPGK.

>Gene0192940.1

MYSQTKRVNVKSLSISFVNIVNKDDHPNLSLSFASSSPHQLLRIQADWFLNLCCRFIM

ASTLFNASCSFPSIKVIDCKSHVGLRSNVSQVRVASLPIATSQRRSLVVRASNGHAKKL

GRSDAECEAAVAAGDVPEAPPVPPKPVAPAGTPVIQPLNLNRRPRRNRASPAVRAAFQ

ETDISPANFVYPLFIHEGEEDTPIGAMPGCYRLGWRHGLLQEVAKARAVGVNSIVLFP

KVPEALKNPTGDEAYNDNGLVPRTIRLLKDKYPDLIIYTDVALDPYSSDGHDGIVRED

GVIMNDETVHQLCKQAVSQARAGADVVSPSDMMDGRVGAIRAALDAEGFQNVSIM

SYTAKYASSFYGPFREALDSNPRFGDKKTYQMNPANYREALIEAREDEAEGADILLVK

PGLPYLDIIRLLRDKSPLPIAAYQVSGEYSMIKAGGVLKMIDEEKVMMESLMCLRRAG

ADIILTYFALQAATYLCNQKR.

>Gene0193660.1

MIVLKWLFLTISPCDAAEPWQLGSQDAATPIMQGIIDLHHDIFFFLILILVFVLWILVRAL

WHFHYKENAIPQRIVHGTTIEILRTIFPSLISMFIAIPSFALLYSMDEVVVDPAITIKAIGH

QWYWTYEYSDYNSSDEQSLTFDSYMIPEEDLELGQSRLLEVDNRVVVPAKTHLRIIVT

SADVPHSWAVPSSGVKCDAVPGRLNQISILVQREGVYYGQCSEICGTNHAFTRAPGNI

GRLLSPLWLSRTTRGCEPPEKQAITASGWSCREPRSKAVDKIEEGARHPVEQRGRLG.

>Gene0516830.1

MATITVVKARQIFDSRGNPTVEVDVHTSTGVKVTAAVPSGASTGIYEALELRDGGSDY

LGKGVSKAVGNVNNIIGPALIGKDPTQQTAIDNFMVHELDGTQNEWGWCKQKLGAN

AILAVSLAVCKAGAVVSGIPLYKHIANLAGNPKIVLPVPAFNVINGGSHAGNKLAMQE

FMILPVGASSFKEAMKMGVEVYHNLKSVIKKKYGQDATNVGDEGGFAPNIQENKEG

LELLKTAIEKAGYTGKVVIGMDVAASEFYSSDKTYDLNFKEENNNGSQKISGDALKD

LYKSFVSEYPIVSIEDPFDQDDWEHYAKMTAECGDNVQIVGDDLLVTNPKRVAKAIAE

KSCNALLLKVNQIGSVTESIEAVKMSKRAGWGVMASHRSGETEDTFIADLSVGLSTG

QIKTGAPCRSERLAKYNQLLRIEEELGSEAVYAGANFRKPVEPY.

>Gene0199220.1

MVWFRAGSSATKLAVRRILNQGTRTPRYLPSQNRSFHSTLYRPNPQSSAAPVPRAVPL

SKLTDSFLDGTSSVYLEELQRAWEADPTSVDESWDNFFRNFVGQAATSPGISGQTIQE

SMRLLLLVRAYQVNGHMKAKLDPLGLEQREIPEDLDLALYGFTEADLDREFFLGVW

QMSGFMSENRPVQTLRSILTRLQQAYCGSIGFEYMHIADRDKCNWLREKIETPTPWR

YNRERREVILDRLAWSTQFENFLATKWTTAKRFGLEGGESLIPGMKEMFDRAADLGV

ESIVIGMSHRGRLNVLGNVVRKPLRQIFSEFSGGIRPVDEVGYTGTGDVKYHLGTSYD

RPTRGGKKIHLSLVANPSHLEAADSVVVGKTRAKQYYSKDLDRTKNLGILIHGDGSFA

GQGVVYETLHLSALPNYTTGGTIHIVVNNQVAFTTDPRAGRSSQYCTDVAKALSAPIF

HVNGDDVEAVVHACELAAEWRQTFHSDVVVDLVCYRRFGHNEIDEPSFTQPKMYK

VIKNHPSTLQIYHKKLLECGEISQQDIDRIQEKVNTILNEEFVASKDYLSKKRDWLSTN

WAGFKSPEQISRVRNTGVKPEILKTVGKAISSLPENFKPHRAVKKVYEQRAQMIETGE

GIDWALAEALAFATLVVEGNHVRLSGQDVERGTFSHRHSVLHDQETGEEYCPLDHLV

MNQDPEMFTVSNSSLSEFGVLGFELGYSMESPNSLVLWEAQFGDFANGAQVIFDQFIS

SGEAKWLRQTGLVVLLPHGYDGQGPEHSSARLERYLQMSDDNPYVIPDMEPTLRKQI

QECNWQIVNATTPANYFHVLRRQLHRDFRKPLIVMAPKNLLRHKDCKSNLSEFDDVQ

GHPGFDKQGTRFKRLIKDQNDHSDLEEGIRRLVLCSGKVYYELDDERKKVGASDVAI

CRVEQLCPFPYDLIQRELKRYPNAEIVWCQEEAMNMGAYSYITPRLWTAMRSLGRGD

MEDIKYVGRGPSAATATGFYTFHVKEQAELVHKAIGKESIS.

>Gene0400410.1

MDYMNGPGRHHLFVPGPVNIPEQVIRAMNRNNEDYRSPAIPALTKTLLEDVKKIFKTT

SGTPFMFPTTGTGAWESALTNTLSPGDRIVSFLIGQFSLLWIDQQKRLNFNVDVVESD

WGQGANLQVLASKLSQDQNHSIKAICIVHNETATGVTNDISAVRTLLDHYKHPALLLV

DGVSSICALDFRMDEWGVDVALTGSQKALSLPTGLGIVCASPKALEATKTSKSLKVFF

DWNDYLKFYKLGTYWPYTPSIQLLYGLRAALDLIFEEGLDNVIARHARLGKATRLAV

EAWGLKNCTQKEEWISNTVTAVMVPPNIDSTEIVKRAWKRYNLSLGLGLNKVAGKVF

RIGHLGHLNELQLLGCLAGVEMILKDVGYPVVLGSGVAAASTYLQHQIPLIPSRI.

>Gene0210950.1

MARKFFVGGNWKCNGTAEEVKKIVNTLNEAQVPSQDVVEVVVSPPYVFLPMVKSIL

RPDFYVAAQNCWVKKGGAFTGEVSAEMLVNLEIPWVILGHSERRALLNETNEFVGD
KVAYALAQGLKVIACVGETLEQRESGSTMDVVAAQTKAIADRVSNWSNVVIAYEPVW
AIGTGKVASPAQAQEVHDELRKWLAKNVSADVAATTRIIYGGSVNGGNCKELGGQA
DVDGFLVGGASLKPEFIDIIKAAEVKKSA.

>Gene0211080.1

MANPRVFFDMTLDGAPAGRIVMELYKDTTPNTAENFRALCTGEKGVGKKGKPLHFK
GSAFHRVIPGFMCQGGDFTAGNGTGGESIYGDKFKDENFVKKHTGAGILSMANSGPN
TNGSQFFICTAETSWLDGKHVVFGKVVEGMEVVKAIEKVGSSSGTTKKKVVVADCG
QI.

>Gene0273040.1

MVNPRVYFDMTVGDKAAGRIVMELYADTVPETAENFRALCTGEKGIGKSGKPLHYK
GSAFHRVIPKFMCQGGDFTAGNGTGGESIYGMKFKDENFVKKHTGPGILSMANAGSN
TNGSQFFICTEKTSWLDGKHVVFGQVVEGMDVVRDIEKVGSDSGRTSKKVVIADCG
QL.

>Gene0420540.1

MASATFSVAKPSLQGFSDFSGLRNSSALPFGKKSSSDEFVSFVSFQTSAMGSNGGYRK
GVTEAKLKVAINGFGRIGRNFLRCWHGRKDSPLDVIAINDTGGVKQATHLLKYDSTL
GIFDADVKPSGDSALSVDGKIIKIVSDRNPSNLPWGELGIDLVIEGTGVFVDREGAGKH
IQAGAKKVLITAPGKGDIPTYVVGVNAELYSHEDTIISNASCTTNCLAPFVKVLDQKF
GIIKGTMTTTHSYTGDQRLLDASHRDLRRARAAALNIVPTSTGAAKAVALVLPNLKG
KLNGIALRVPTPNVSVVDLVVQVSKKTFAEEVNAAFRDAAEKELKGILDVCDEPLVS
VDFRCSDVSSTIDSSLTMVMGDDMVKVIAWYDNEWGYSQRVVDLADIVANNWK.

>Gene0217460.1

MSTNDADNVQTPLNGGSGTDLHTPAADVSAANAQANAATLEEFKKMFATYEKRSEE
QDKLVNTLTKQVETLTARTQAIPRKSTDETSDIRDLRDFITKTAAEVRAVKSQIHHATS
AAPEIDRLLEGARKTPFTSRISDMRVSDPGKIKVPKYDVAIDALRKTLWYKSKIRKWIT
LDKPRTIQDTLHRATDYIIVEEETKVLSQKHKAARPSSKDVDPKGKKKNSRYDENTFC
EFHQSRGHSTTNCKVLGARLAAKLLAGELSEVTSVKDLILDSDRPPKTDRNPPAEKSP
QRNQHGDKRGRWPDDKANDNNRRRVNMII.

>Gene0542280.1

MSATLTGSGTALGFSCSSKISKRVSSSPSTRCSIKMSVSVDEKKKSFTLQKSEEAFNAA
KNLMPGGVNSPVRAFKSVGGQPVLIDSVKGSKMWDIDGNEYIDYVGSWGPAIIGHA
DDEVLAALAETMKKGTSFGAPCLLENVLAEMVISAVPSIEMVRFVNSGTEACMGVLR
LARAFTNKEKFIKFEGCYHGHANAFLVKAGSGVATLGLPDSPGVPKAATSDTLTAPYN
DIEAVAKLFEAHKGEISAVILEPVVGNSGFITPTPEFINGLRQLTKDNGALLIFDEVMTG
FRLAYGGAQEYFGITPDLTTLGKIIGGGLPVGAYGGRRDIMEMVAPAGPMYQAGTLS
GNPLAMTAGIHTLKRLKQPGTYEYLDKITKELTNGILEAGKKTGHPMCGGYISGMFG
FFFAEGPVYNFADAKKSDTEKFGKFFRGMLEEGVYFAPSQFEAGFTSLAHTSEDIQFTI
SAAERVLGRI.

>Gene0295530.1

MFSAQNKIHKDKGVAPTDFEQEVAQAFFDLENTNQELKSDLKDLYINQAVQMDISGG
RKAIVIYVPFRLRKAFRKIHPRLVRELEKKFSGKDVVFVATRRIMRPPKKGSAVQRPRN
RTLTSVHEAMLEDVAYPAEIVGKRTRYRVDGTKIMKVFLEPKERNNTEYKLETMVGV
YRKLTGRDVVFEYPTIEG.

>Gene0400910.1

MASHTFLSFSSPPRLLVSPPSTLRSPFVGVSLNLHRPQSVSFSASKKSLTVVSAAKKAVA
VLKGNSDVEGVVTLTQDDSGPTKVSVRITGLTPGPHGFHLHEFGDTTNGCISTGPHFN
PNNMTHGAPEDEIRHAGDLGNIIANADGVAETTLVDNQIPLTGPNSVVGRAFVVHELK
DDLGKGGHELSLTTGNAGGRLACGVVGLTPL.

>Gene0500150.1

MVASAFLPELWTEILIPVCAVVGIAFSLFQWFIVSRVRVSADQGASSSSSGGSKNGYGD
YLIEEEEGVNDQSVVAKCAEIQTAISEGATSFLFTEYRYVGVFMVIFAAIIFVFLGSVEG
FSTENKPCTYDETKTCKPALATAAFSTIAFVLGAVTSVLSGFLGMKIATYANARTTLEA
RKGVGKAFIVAFRSGAVMGFLLAASGLLVLYVTINVFKIYYGDDWEGLFEAITGYGL
GGSSMALFGRVGGGIYTKAADVGADLVGKIERNIPEDDPRNPAVIADNVGDNVGDIA
GMGSDLFGSYAEASCAALVVASISSFGINHDFTAMCYPLLISSMGILVCLITTLFATDFF
EIKAVKEIEPALKNQLIISTVIMTVGIAVVSWVGLPSSFTIFNFGTQKVVQNWQLFLCV
CVGLWAGLIIGFVTEYYTSNAYSPVQDVADSCRTGAATNVIFGLALGYKSVIIPIFAIAV
SIFVSFSFAAMYGVAVAALGMLSTIATGLAIDAYGPISDNAGGIAEMAGMSHRIRERTD
ALDAAGNTTAAIGKGFAIGSAALVSLALFGAFVSRAGVHTVDVLTPKVIIGLLVGAML

PYWFSAMTMKSVGSAALKMVEEVRRQFNTIPGLMEGTAKPDYATCVKISTDASIKEM
IPPGCLVMLTPLIVGFFFGVETLSGVLAGSLVSGVQIAISASNTGGAWDNAKKYIEAGV
SEHAKSLGPKGSEPHKAAVIGDTIGDPLKDTSGPSLNILIKLMAVESLVFAPFFATHGGI
LFKYL.

>Gene0489480.1

MGISRDSIHKRRATGGKQKQWRKKRKYEMGRQPANTKLSSNKTVRRIRVRGGNVK
WRALRLDTGNYSWGSEAVTRKTRVLDVVYNASNNELVRTKTLVKSAIVQVDAAPFK
QWYLQHYGVEVGRKKKNASAVAAKKDGEEGEEVAPAAAAPEETKKSNHVLRKIESR
QEGRSLDSHIEDQFASGRLLACISSRPGQCGRADGYILEGKELEFYQKKIQKKKGKGA
A.

>Gene0225980.1

MAMMSASSAFVLTSNVTASAGVSSSRNSVSFLPMRNAGSRLVVRAAEDAAPETSSSE
GAPATAVAPAAAAATKPKPPPIGPKRGSKVKILRRESYWFKNVGSVVAVDQDPKTRYP
VVVRFAKVNYANISTNNYALDEIEELKA.

>Gene0226140.1

MAAAAIGAAVSYPSSKSSSLPSKTSFVSPQRIFLNNKSTLCYREACVYGGRVKAQVST
EAPVKVAKESKKQEEGIVVNKFKPKEPYTGRCLLNTRITGDDAPGETWHIVFTTEGEV
PYREGQSIGIIPEGIDKNGKPHKLRLYSIASSAIGDFGDSKTVSLCVKRLVYTNESGEVV
KGVCSNFLCDLKPGDEAKITGPVGKEMLMPKDPNATIIMLGTGTGIAPFRSFLWKMFF
EEHEDYKFNGLAWLFLGVPTSSSLLYKEEFEKMKEKNPENFRLDFAVSREQTNDKGE
KMYIQTRMAEYAEELWELLKKDNTFVYMCGLKGMEKGIDEIMVSLAAKDGIDWFE
YKKQLKKSEQWNVEVY.

>Gene0230210.1

MASLGVSEMLGTPLNFRAVSRPSAPLASIPATFKTVALFSKKKPAPPPKAKTVSAANDE
LAKWYGPDRRIFLPDGLLDRSEIPEYLNGEVAGDYGYDPFGLGKKPENFAKYQAFELI
HARWAMLGAAGCIIPEALNKYGANCGPEAVWFKTGALLLDGNTLSYFGKNIPINLVL
AVVAEVVLLGGAEYYRITNGLDFEDKLHPGGPFDPLGLAKDPEQGALLKVKEIKNGR
LAMFSMFAFFIQAYVTGEGPVENLSKHLSDPFGNNLLTVIAGTAERAPTL.

>Gene0266080.1

MASMSAVLSKSPFLSQPLSKSPSSDLPFSAATVSFPSKSHRRGGVIRAGLISPDGGKLVE

LVVAEPRRREKKHEAGELPRVELTAIDVQWMHVLSEGWASPLGGFMRESEFLQTLHF
NSLRLDDGSVVNMSVPIVLAIDDEQKASIGESKRVALVGSDGNPVAILSDIEIYKHPKE
ERIARTWGTTAPGLPYVEEAITNAGNWLIGGDLEVLEPVKYNDGLDRFRLSPAELRKE
LEKRGADAVFAFQLRNPVHNGHALLMTDTRRRLLEMGYKNPILLLHPLGGYTKADD
VPLSWRMKQHEKVLEDGVLDPETTVVSIFPSPMHYAGPTEVQWHAKARINAGANFY
IVGRDPAGMGHPVEKRDLYDADHGKKVLSMAPGLERLNILPFRVAAYDKTQGKMAF
FDPSRPQDFLFISGTKMRTLAKNKENPPDGFMCPGGWQVLVDYYDSLTPAGKLPVPA.

>Gene0240170.1

MASANKEMAVYCFDTLVSHYNNEDSPPPAFDDANHPLFVTWKKIVNGGEPRLRGCIG
TLEARRLISGFKDYALTSALRDRRFPPIQPKELPFLQCTVSVLTDYEDAEDYLDWEVG
THGIIIEFTEPVTNIKRNATYLPEVPAHEGWTKIEAIDSLVRKAGYNGEITEAVRRRIQLT
RYQSTLFSMHYSEYLSYVKATRGLVPAINGTSKPLS.

>Gene0241630.1

MARNDPNPFADEETNPFADNKSVPPASNSYLKPLPPEPHDRGSTVDIPLDSSQDLRAK
EMELQAKENELKRKEQELKRREDAIARTGIVIEEKNWPDFFPLIHHDITNEIPIHLQKIQ
YVAFATLLGLIACLLWNIVAVTVAWINGGGPTIWLLSIIYFISGVPGAYVLWYRPLYRAT
RTDSALKFGTFFLFYLFHIAFCGFAAVAPPVIFRGKSLTGFLPALEFLTTNVVVGILYFIG
AGFFCIETLLNIWVIQQVYAYFRGSGKAAQMKREATNSMVRAL.

>Gene0255380.1

MFVITRLICRRIHGTSDVTVPKLSGFSIVSPKYVEVEYADGTKFKFSSEFLRVNSPAAD
GKIRSIGGDKVISGRRYVGIMSAEPVGNYGVRTGIYPWDYFYELGSN

>Gene0414030.1

MASKLIQLKSKACEASKFVSKHGTTYYKQLLEKNKQYIQEPATVEKCQELSKQLLYT
RLASIPGRTESFWKEVDHVKGLWKNRADLKVEDAGIAALFGLECFAWYCAGEIVGR
GFTFTGYYP.

>Gene0392000.1

MRGGSLWQLGQSITRRLAQSDKKPLSPRRHFASGADLKKTALYDFHVAHGGKMVPF
AGWSMPIQYKDSIIDSTVNCRVNGSLFDVAHMCGLSLKGKDCVPFLETLVVADVAGL
APGTGSLTVFTNEKGGAIDDSVITKVTDEHIYLVVNAGCRDKDLAHIEEHMKAFKSK
GGDVSWHIHDERSLLALQGPLAAPVLQHLTKEDLSKLYFGQFQILDINGSTCFLTRTG

YTGEDGFEISVPSEHAVDLAKAILEKSEGKVRLTGLGARDSLRLEAGLCLYGNDMEQ
HITPVEAGLTWAIGKRRRAEGGFLGADVILKQLQDGPTIRRVGFFSSGPPARSHSEVHD
ENGNKIGEITSGGFSPNLKKNIAMGYVKSGQHKNGTKVKILVRGKPYEGNITKMPFVA
TKYYKPT.

>Gene0252990.1

MASSVVTSFQPRSAFLGDRNVFKVLATPSAQVGYSRKTIQCKESRIGKQPIAVPTNVTI
ALEGQDLKVKGPLGELALTYPREVELIKEDAGVLRVKKTVETRRANQMHGLFRTLTD
NMVVGVSKGFEKKLILVGVGYRATVEGKELVLNLGFSHPVKMQIPESLKVKVEENTR
ITVSGYDKSEIGQFAATVRKWRPPEPYKGKGVKYSDEIVRRKEGKAGKKK.

>Gene0260890.1

MAREALYAYVRTYDEEYVLKRKLRSRVLNKHSRVGCQVVLRQELQGMVVAAPEAKP
CDIA.

>Gene0262760.1

MSPVRRAMALASTTLPAKTGLSLWCPTSPSLARRLPARFSSRIASRGLVTASFANENRE
FVIIGGGNAAGYAARTFVENGMADGRLCIVTKEAYAPYERPALTKAYLFPPEKKPARL
PGFHTCVGGGGERQTPDWYKEKGIEMIYEDPVTGADFEKQTLTTNTGKQLKYGSLIIA
TGCTASRFPDKIGGNLPGVHYIREVADADSLISSLGKSKKVVIVGGGYIGMEVAAAAV
AWNLDTTIVFPEDQLLQRLFTPSLAQRYEELYRQNGVKFVKGASINNLEAGSDGRVTA
VKLADGSTIEADTVVIGIGAKPAIGPFETLAMNKSIGGIQVDGLFRTSTPGIFAIGDVAA
FPLKIYDRMTRVEHVDHARRSAQHCVKSLLTAHTDTYDYLPYFYSRVFEYEGSSRKV
WWQFYGDNVGETVEVGNFDPKIATFWIDSGRLKGVLVESGSPEEFQLLPKLARSQPIV
DKAKLASASSVEEALEIAQAALQS.

>Gene0279090.1

MSTAPLSGFFLTSLSPSQSSLKKVSLRSSPTVASLPSSSSSSSSSSRSVPTLIRNEPVLAA
PAPIITPYWSEEMGSESYQEATEALKKLLIEKEELKTVAAAKVEQITAELKTGAPSDKK
AFDPVENIKQGFITFKKEKYETNPALYGELAKGQSPKYMVFACSDSRVCPSHVLNFQP
GDAFVVRNIANMVPPFDKVKYGGVGAAIEYAVLHLKVENIVVIGHSACGGIKGLMSF
PLDGNNSTDFIEDWVKICLPAKSKVKSEVGDSAFEDQCSRCEREAVNVSLANLLTYPF
VREGLVKGTLALKGGYYDFIKGAFELWGLEFGLSETSSVKDVATILHWKL.

>Gene0262810.1

MATAPLSGFFLTSLSPSQPSLQKQTLRSSPTVACLPSSSSSSSSSSSSRSVPTLIRNEPVFA

APAPIITPYWSEEMGSEAYEEAIEALKKLIIEKEELKTVAAAKVEQATAALQTGTSSDK

KAFDPVENIKQGFITFKKEKYETNPALYGELAKGQSPKYMVFACSDSRVCPSHVLNFQ

PGEAFVVRNIANMVPPFDKVKYGGVGAAIEYAVLHLKVENIVVIGHSACGGIKGLMS

FPLDGNNSTDFIEDWVKICLPAKSKVISELGDSAFEDQCGRCEREAVNVSLANLLTYPF

VREGLVKGTLALKGGYYDFIKGAFELWGLEFGLSETSSV.

>Gene0399650.1

MAVPLLTKKVVKKRSAKFIRPQSDRRITVKESWRRPKGIDSRVRRKFKGVTLMPNVG

YGSDKKTRHYLPNGFKKFVVHNTSDLELLMMHNRTYCAEIAHNVSTKKRKAIVERA

SQLDIVVTNRLARLRSQEDE.

>Gene0484520.1

MATAALLRSIRRRELASAPFSAYKFIQSSGKASLNGQNWRSFSRAFSLLEMTSSVLTW

VLLTHNPKVIENAEGARTTPSVVAFNPKGELLVGTPAKRQAVTNPTNTLFGTKRLIGR

KFDDPQTQKEMKMVPYKIVRAPNGDAWVEANGQQYSPSQVGAFVLTKMKETAEAY

LGKSVKKAVVTVPAYFNDAQRQATKDAGRIAGLDVERIINEPTAAALSYGMTNKEGL

IAVFDLGGGTFDVSILEISNGVFEVKATNGDTFLGGEDFDNALLDFLVNEFKTSEGIDL

AKDRLALQRLREAAEKAKIELSSTSQTEINLPFITADASGAKHFNITLTRSRFEALVNGL

IERTRDPCKNCLKDAGISAKEVDEVLLVGGMTRVPKVQTVVAEIFGKTPSKGVNPDEA

VAMGAALQGGILRGDVKELLLLDVTPLSLGIETLGGVFTRLITRNTTIPTKKSQVFSTA

ADNQTQVGIRVLQGEREMASDNKLLGEFDLVGIPPSPRGIPQIEVTFDIDANGIVTVSA

KDKTTGKEQQITIRSSGGLSEDDIQKMVRDAELHAQKDKERKDLIDTKNTADTTIYSI

EKSLGEYREKIPSEVAKEIEDAVADLRTASSGDDVNEIKAKIDAANKAVSKIGEHMSGG

GGSGGDSTPGGGGAQGGSDQTPEADYEEVKK.

>Gene0275610.1

MGSQSYEDALEALKKLVIEKDDLKTVAAAKVEQITAELKKISSSDGKPFDPVERIKEG

FVTFKKEKYDTNPALYGELAKGQSPKYMVFACSDSRVCPSHVLNFHPGDAFVVRNIA

NMVPPYDQVKYAGVGAAIEYAVLHLKVENIVIIGHSACGGIKGLMSFALDGNNSTDFI

EDWVKICLPAKSKVLAEAESSAFEDQCGRCEREAVNVSLANLLTYPFVREGVVKGTL

ALKGGYYDFVKGAFELWELQFGLSPVHPI.

>Gene0340240.1

MALEYESLNENVKKCQYAVRGELYLRASELQKEGKKIIFTNVGNPHALGQKPLTFPR
QVVALCQAPFLLDDPNVGMIFPADAIARAKHYLSLTSGGLGAYSDSRGLPGVRKEVAE
FIQRRDGYPSDPELIFLTDGASKGVMQILNCVIRGERDGILVPVPQYPLYSATISLLGGS
LVPYYLDESENWGLDVNNLRQSVAQARSQGISVRAMVIINPGNPTGQCLSEANLREIL
RFCHSEKLVLLGDEVYQQNIYQDERPFISSKKVLMDMGSPFSKEVQLVSFHTVSKGY
WGECGQRGGYFEMTNFPPRVVEEIYKVASIALSPNVSAQIFMGLMVSPPKPGDISYDQ
FARESKGILESLRRRAKIMTDGFNSCKNVVCNFTEGAMYSFPQIKLPPGALQAAKQA
GKVPDVFYCLKLLEATGISTVPGSGFGQKEGVFHLRTTILPAEEEMPEIMDSFKKFNDE
FMTQYENSFGYSRM.
>Gene0285620.1
MATVKLSLSSPSSIQSKLSAASFIPNAARAFNATPLSAKASRSRFMGKCLRSTPMITHR
GSRSGGIKCSASSSPVTLPSALLFDCDGVLVDTEKDGHRISFNDTFKERDLGVTWDVD
LYGELLKIGGGKERMTAYFNKVGWPEKAPKDEAERKAFIAGLHKQKTELFMVLIEKK
LLPLRPGVAKLVDQALTNGVKVAVCSTSNEKAVSAIVSCLLGPERAENIKIFAGDVVPK
KKPDPAIYNLAAETLGVDPSKCVVVEDSAIGLAAAKAAGMTCIVTKSGYTADEDFVN
ADAVFDCIGDPPEERFDLSFCGSLLQKQFVS.
>Gene0290110.1
MAMMSASSVFLLPANVTAPAGASSSRNSVSFLPMRNAGSRLVVRAADEAAPEPAAPE
GAPATTAAPAAAAATKPKPPPIGPKRGAKVKILRRESYWFKSVGSVVAVDQDPKTRYP
VVVRFAKVNYANISTNNYALDEIEEVKA.
>Gene0354140.1
MIARRIWRSHRFLRPFSSSSVCAPPLLAPYHSQSFASPTSRPFLVPSLSVMKWSGGGGS
RSLFSNEAMSIDSNAGGGFIDVPLAQTGEGIAECELLKWFVKEGDPVEEFQPLCEVQS
DKATIEITSRFKGKVALISHAPGDIIKVGETLVKLAVEDANDALQVSSDTPKNVEPICSK
PKLDTLVGALSTPAVRTLAKDLGIDINLVIGSGKDGRVLKEDVLRFGGQKENVTEDPVI
RGDSVSTNFEDQIVPLRGFNRAMVKTMTMATKVPHFHFVEEINCDALVKLKHFFKEH
NTDSTVKHTFLPTLIKSLSMALTKYPYVNGCFNEESLEIVLKGSHNIGVAMATEHGLV
VPNIKNVQSLSLLEITKEMSRLQHLATNNKLSPEDVTGGTITLSNIGAIGGKFGSPLLNL
PEVAIIALGRIEKVPKFKEDGSVYPASTMMVNIAADHRVLDGATVARFCCQWKEYIEK
PELLMLQMR.

>Gene0290320.1

MGKGPGLYSEIGKKARDLLYKDYQGDQKLSITTYSSTGVAITTSGTNKGDLFLGDVV

TQIKNKNFTADIKVASDSSILTTFTYDEATPGLKAIVSAKVPDQKSAKVELQYMHPHA

GICTSVGLTANPVVNFSGVIGTSVLALGTDVSFDTESGNFKHFNTGVSFTKDDLIASLT

LNDKGEKLTASYYHIVNPLKNTVVGAEVSHNLKSQVNSITVGTQHALDPLTTVKARV

NNAGIANALIQHEWRPKSFITISGEVDSKAIEKSAKVGFALALKP.

>Gene0412400.1

MHCIRSSILQHLRLRVPVRSVLLLEKENVFISKMNFTTEHDGGQDQVLSKVIELVKKY

DTANASKVTETADFKKDLSLDSLDRVEIVMAIEEEFSVEIPDEKADKLTCCADIASFIVS

ETQSKASES.

>Gene0368210.1

MYKTTSSSLLRAASSRSPLLSSRSSLTQSSSSASPSPPSSLLGRRSFGTSSPAFRSLPRWS

HCLHSRPSPFRLSSQIRAVSPGLDRLERNFSSMASEHPFKGIFTTLPKPGGGEFGKFYSL

PALNDPRIDKLPYSIRILLESAIRNCDNFQVTKEDVEKIIDWEKTAPKQVEIPFKPARVL

LQDFTGVPAVVDLACMRDAMNKLGSDSNKINPLVPVDLVIDHSVQVDVARSENAVQ

ANMELEFQRNKERFAFLKWGSTAFQNMLVVPPGSGIVHQVNLEYLGRVVFNTQGVL

YPDSVVGTDSHTTMIDGLGVAGWGVGGIEAEATMLGQPMSMVLPGVVGFKLSGKM

RNGVTATDLVLTVTQILRKHGVVGKFVEFYGDGMSGLSLADRATIANMSPEYGATMG

FFPVDHVTLQYLKLTGRSDETVAMIEAYLRANNMFVDYNEPQQDRAYSSYLELNLDN

VEPCISGPKRPHDRVPLKEMKADWNSCLDSKVGFKGFAIPKEAQEKVANFSFNGKPA

EITHGSVVIAAITSCTNTSNPSVMLGAGLVAKKACDLGLQVKPWIKTSLAPGSGVVTK

YLLKSGLQEYLNQQGFNIVGYGCTTCIGNSGEINESVGAAITENDIVAAAVLSGNRNF

EGRVHPLTRANYLASPPLVVAYALAGTVNIDFETEPIGTGKNGKDVFLRDIWPTTEEIA

EVVQSSVLPDMFRATYESITKGNPMWNELSVPENTLYSWDPKSTYIHEPPYFKDMTM

EPPGPHSVNDAYCLLNFGDSITTDHISPAGNIQKDSPAAKYLMERGVDRKDFNSYGSR

RGNDEIMARGTFANIRIVNKLMNGEVGPKTVHIPSGEKLSVFDAAMRYKSSGEDTIIL

AGAEYGSGSSRDWAAKGPMLQGVKAVIAKSFERIHRSNLVGMGIIPLCFKSGEDADT

LGLTGHERYTIHLPTDISEIRPGQDVTVTTDNGKSFTCTVRFDTEVELAYFNHGGILPY

VIRNLSKQ.

>Gene0296800.1

MAAAFSSRFLNTVRCNRLASVSSVYQNGMMRYSSSVPSDSDTHDDFKPTQKVPPGG
STDSLKDLVENDVKENPVMLYMKGVPEAPQCGFSSLAVRVLQQYNVPIGARNILEDP
ELKNAVKSFSHWPTFPQIFIKGEFIGGSDIILNMHKEGELEEKLKDVSANHKSQ.

>Gene0300820.1

MERARRLAYRGIVRRLVNESKRHRNGEITPHHVPSVVPHAPSRYISSLSPFLSNNHHN
RSVNLPKHHNHNQTRSISVDAVKAGDTFPRRHNSATPDEQAHMAKYCGYDHIDSLV
DDTVPKQIRIDSMKFSKFDGGLTESEMIAHMTELASKNKVFKSFIGMGYYNTHVPTVI
IRNILENPAWYTQYTPYQAEISQGRLESLLNFQTMITDLTGLPMSNASLLDEGTAAAEA
MAMCNNIQKGKKKTFVIASNCHPQTIEICKTRADGFDLKVVTKDLKEIDYSCGDVCG
VLVQYPGTEGEVLDYGEFVKNAHANGVKVVMATDLLALTVLKPPGEFGADIVVGSA
QRFGVPMGYGGPHAAFLATSSEYKRMMPGRIIGVSVDSTGKPALRMAMQTREQHIR
RDKATSNICTAQALLANMAAMYAVYHGPAGLKSIAERVHGLAGIFSLGLKKLGVAEV
QDLPYFDTVKIKCSDAHAIADAAVKSEMNLRVVDSNTITASFDETTTLDDVDKLFKVF
ASGKPVPFTAESLAPEVQNSIPSNLTRESTYLTHPIFNMYHTEHELLRYIHKLATKDLSL
CHSMIPLGSCTMKLNATTEMMPVSWPSFTDIHPFAPIDQAQGYQEMFSTLGDLLCTIT
GFDSLSLQPNAGASGEYTGLMVIREYHRSRGDHHRNVCIIPVSAHGTNPASAAMCGM
KIITVGTDAKGNINMEEVRKAAEDNKDSLAALMVTYPSTHGVYEETIDEICKIIHDNG
GQVYMDGANMNAQVGLTSPGFIGADVCHLNLHKTFCIPHGGGGPGMGPIGVKKHLA
PFLPSHPVIPTGGFPQPEKTEPLGPIAAAPWGSALILPISYSYIAMMGSRGLTEASKIAIL
NANYMAKRLEKHFPVLFRGANGTVAHEFIIDLRGFKNTAGIEAEDVAKRLMDYGFHA
PTMSFPVSGTLMIEPTESESKAELDRFCDTLISIREEIAQIEKGNADVQNNVLKGAPHSP
AMLMSDTWKKPYSREYAAFPTPWLRSAKFWPTTGRVDNVYGDRKLVCTLLPEEEQV
AAAVSA.

>Gene0301160.1

MVFFRSVAAFTRLRSRVGQQSSLGSSVRWIQMQSSTDVDLKSQLQELIPEQQDRLKKL
KSEHGKVQLGNITVDMVIGGMRGMTGLLWETSLLDPEEGIRFRGLSIPECQKVLPVA
QSGGEPLPEGLLWLLLTGKVPSKEQVEALSKDLANRAAVPDYVYNAIDALPSTAHPM
TQFASGVMALQVQSEFQKAYENGIHKSKFWEPTYEDCLNLIARVPVVAAYVYRRMY
KNGDSIPSDKSLDYGANFSHMLGFDDAKMKELMRLYITIHSDHEGGNVSAHTGHLVG
SALSDPYLSFAAALNGLAGPLHGLANQEVLLWIKSVVEECGENISKDQLKEYVWKTL

NSGKVVPGYGHGVLRKTDPRYVCQREFALKHLPDDPLFQLVSKLYEVVPPVLTELGK
VKNPWPNVDAHSGVLLNHYGLTEARYYTVLFGVSRSLGICSQLIWDRALGLALERPK
SVTMDWLEAYCEKAKASSP.

>Gene0308110.1

MACPSLLQSSASSFHGRFTSLAAPSSVRVLPPPLRNVVKVSASGTVLVEKSEAEKVQR
LKTAYLERIIPALKEEFKYINIHQVPKVQKIVVNCGIGDAAQNDKGLEAAMKDIALITG
QKPVKTRARASIATFKIREDQPLGIAVTLRGDVMYSFLDRLINLALPRTRDFQGVSPSS
FDGNGNYSIGVKDQSVFPEIRFDAIGKARGMDVCISTTAKTDQEGQKLLALMGMPFR
EGSSANTGAPVRKKKLKSHHFDAKGKGKGKR.

>Gene0315380.1

MANSTILLDYWPSMFCMRARVALREKGVVFEAREEDLTNKSPLLLQSNPIHKKVPVLI
HNGKPICESLNVVQYVDEAWSDKNPFFPSDPYGKAQARFWADFVDKKFSDAQFKIW
GKKGEEQAAGVKEFIEAVKILEAELGDKPYFGGDSFGYVDIALITFYSWFGAYEKFGN
FSIEAESPKLIAWAKRCMEKESVSKSLPDQEKIVAYAAEFRKNNL.

>Gene0318540.1

MAATQLTASPVTVSARSLASLRASSAKFSSFGTLKPGTLRQSLFRPLVVRAASVVAPK
YTSIKPLGDRVLVKIKEAEEKTMGGILLPSTAQSKPQGGEVVAVGEGRTIGKNKIDINV
PTGAQIIYSKYAGTEVEFNDVKHLILKEDDIVGLLETDDIKDLKPLNDRVFIKVAEAEE
KTAGGLLLTETTKEKPSIGTVIAVGPGSLDEEGKLQPLPISTGSTVLYSKYAGNDFKGK
DGSNYIALRASDVMAILS.

>Gene0318750.1

MALAGRIRSGISFFKNISVSDARSYPRGGSLIPSLRDYATASAQNTVNVKVPVALVGES
GNFASWLYIAAVKMNSLEKIETDLSELVEAMKTSPIFAQFTKDPSVPRETRLAAIVDVC
DKAKFAEPTKNFLSLLAENGKLKNLDVIVKKFMQLTTAHRGDVKVLVTTVMPLPPAE
EKELKDTLQEIIGEGKKVTVEQKIDPSIYGGLIVEFQQKVLDMSIRTRAQQMERLLREP
VDFTNL.

>Gene0514970.1

MATDTPIRIGVMGCAEIARKVSRAIHLAPNATIAAVASRSLEKAKSFASSNGYPESTKIH
GSYESILEDPEVDALYVPLPTSLHVEWAIRAAEKGKHILLEKPVAMNVAEFDKIVAACE
ANGVQIMDGTMWVHNPRTAKLKEFLSDSDRFGQLKTVQSCFSFAGDEDFLKNDIRV

KPGLDGLGALGDAGWYAIRATLLANNFELPNTVTAFPGAVLNEAGVILSCGASLTWE
DGRTATIYCSFLANLTMEITAIGTNGTLRVHDFIIPFQETQASFTTSTKAWFNELVTAWV
NPPSEHTVKTELPQEACMVREFARLVGEIKNKGAKPDGFWPSISRKTQLVVDAVKESV
DKNYEQISLSGR.

>Gene0324910.1

MGNNIDTGNFGHLGITTAEMESLASFCEAVLPTVSPPEEYSGGDDHYRNKETLRSFFF
TSGSRTPVVRESIELITKRGTVETYLVTRLVLFLLATRLGTLLICGTECLVSRWPFVEKF
SELSLEKRERVLQKQFRNWLLTPIRAAFVYIKVAFLFCFFSRVSPNGQNPAWEAIGYNV
NTNENKPSETHQERPLEKGMVETMQETEQTLLESLSQKGLEAETDHDTIRIKCDAVV
VGSGSGGGVAASVLAKAGMKVVVMEKGSYYTPSTYPSTEGPGMDKLYENGGILPTI
DGNMMVLAGATVGGGSAVNWSACIKTPKSVLQEWSEDRKIPLYATKEYVSAMELVW
KRMGVTETCELEGFKNQVLRKGCENLGINVENVARNSSERHYCGSCGYGCRQGDKK
GSDRTWLVDAVSHGAVILTGCKAERFILEKNGNNVGRKKMKCLGVMAKSLNGNIAK
KIKIEARVTVSAAGSLLTPPLMIKSGLKNRHIGKNLHLHPVLMTWGYFPNKESSSFNF
KGRSYEGGIITSMSKVLSEDSEVRAIIETPALGPASFSVLCPWISGLDMKKRMSRYSRT
ANLITIVRDRGSGEVRTEGRISYVVDKIDRENITAGLRQSLRILIAAGAEEVGTHRSDG
QKLICKGVDEKLIEEFLDSVSAEEGPKAMTENWSVYSSAHQMGSCRIGVDEDEGAID
LDGESWEADKLFVCDGSVLPSAVGVNPMITIMSTAYCISTRIVKSINEL.

>Gene0327380.1

MSGAGKKVADVAFKASRTIDWEGMAKVLVTDEARREFSNLRRAFDEVNTQLQTKFS
QEPEPIDWDFYRKGIGSGIVDMYKEAYDSVEIPKYVDNVTPEYKPKFDALLVELKEAE
QKSLKESERLEKEIIDVQEISKKLSTMTADEYFEKHPELKKKFDDEIRNDYWGY.

>Gene0330500.1

MAIKSDNKTRSSVQIFIVFSLCCFFYILGAWQRSGFGKGDSIALEMTNSASAADCNIVP
SLNFETHHAGESSISSAKVKAFEPCAARYTDYTPCHDQKRAMTFPRESMIYRERHCVP
ENEKLRCLVPAPKGYVTPFSWPKSRDYVPYANAPYKALTVEKAIQNWIQYEGEVFRF
PGGGTQFPQGADKYIDQLASVIPMENGTVRTALDTGCGVASWGAYLWSRNVRAMSF
APRDSHEAQVQFALERGVPAVIGVLGSIKLPYPTRAFDMAHCSRCLIPWGANDGMYL
MEVDRVLRPGGYWILSGPPINWKINYKAWQRPREDLEEEQRKIEEAAKLLCWEKKY
EHGEIAIWQKRVNDEACRSRQDDPRANFCKTDDTDDVWYKKMEACITPYPETSSSDE

VAGGELQVFPDRLNAVPPRISSGSVSGVTADAYEDDNRQWKKHVKAYKRINGLLDTG
RYRNIMDMNAGFGGFAAAIESQKLWVMNVVPTIAEKNRLGVVYERGLIGIYHDWCE
AFSTYPRTYDLIHANHLFSLYKNKCNADDILLEMDRILRPEGAVIIRDDVDTLIKVKRII
SGMRWDSKLVDHEDGPLVNEKVLIAVKQYWVTNSTASH.

>Gene0332720.1

MEFRGDANKRIAMISAHLQPSFIPQLEAKNSVMGRENCRAKGGNPGFKVAILGAAGG
IGQSLSLLMKMNPLVSLLHLYDVVNAPGVTADVSHMDTGAVVRGFLGAKQLEDALT
GMDLVIIPAGVPRKPGMTRDDLFKINAGIVKTLCEGVAKCCPNAIVNLISNPVNSTVAI
AAEVFKKAGTYDPKKLLGVTTLDVARANTFVAEVLGLDPREVDVPVVGGHAGVTIL
PLLSQVKPPSSFTPSEIEYLTNRIQNGGTEVVEAKAGAGSATLSMAYAAAKFADACLR
GLRGDANVIECSFVASQVTELAFFATKVRLGRTGAEEVFQLGPLNEYERVGLEKAKEE
LAGSIQKGVDFIRK.

>Gene0333600.1

MSPQTETKASVGFKAGVKEYKLNYYTPEYETKDTDILAAFRVTPQPGVPPEEAGAAV
AAESSTGTWTTVWTDGLTSLDRYKGRCYHIEPVPGEETQFIAYVAYPLDLFEEGSVTN
MFTSIVGNVFGFKALAALRLEDLRIPPAYTKTFQGPPHGIQVERDKLNKYGRPLLGCTI
KPKLGLSAKNYGRAVYECLRGGLDFTKDDENVNSQPFMRWRDRFLFCAEAIYKSQA
ETGEIKGHYLNATAGTCEEMMKRAIFARELGVPIVMHDYLTGGFTANTSLAHYCRDN
GLLLHIHRAMHAVIDRQKNHGMHFRVLAKALRLSGGDHVHAGTVVGKLEGDRESTL
GFVDLLRDDYVEKDRSRGIFFTQDWVSLPGVLPVASGGIHVWHMPALTEIFGDDSVL
QFGGGTLGHPWGNAPGAIANRVALEACVQARNEGHDLAVEGNEIIREACKWSPELA
AACEVWKEITFNFPTIDKLDGQDYKLD.

>Gene0465750.1

MFRSALVRSSASAKQSLLRRSFSSGSVPERKVAILGAAGGIGQPLALLMKLNPLVSSLS
LYDIANTPGVAADVGHINTRSQVVGYMGDDNLAKALEGADLVIIPAGVPRKPGMTRD
DLFNINAGIVKNLCSAIAKYCPHALVNMISNPVNSTVPIAAEIFKKAGMYDEKKLFGV
TTLDVVRAKTFYAGKANVPVAEVNVPVIGGHAGVTILPLFSQATPQANLSGDVLTALT
KRTQDGGTEVVEAKAGKGSATLSMAYAGALFADACLKGLNGVPDVVECSYVQSTIT
ELPFFASKVRLGKNGVEEVLDLGPLSDFEKEGLEALKPELKSSIEKGVKFANQ.

>Gene0337450.1

MASTSSLALSQALLARAISHHGSDQRISLPSPFSRASASSRRRSNAATTKLRSLRPLVV
RAAAVDTLEPTTDASIVDKSVNTIRFLAIDAVEKAKSGHPGLPMGCAPMAHILYDEVM
RYNPKNPYWFNRDRFVLSAGHGCMLLYALLHLAGYDSVLEEDLKSFRQWGSKTPGH
PENFETPGIEVTTGPLGQGIANAVGLALAEKHLAARFNKPDAEVVDHYTYVILGDGC
QMEGISNEAASLAGHWGLGKLIAFYDDNHISIDGDTEIAFTENVDQRFEALGWHVIW
VKNGNTGYDEIRAAIKEAKAVTDKPTLIKVTTTIGYGSPNKANSYSVHGAALGEKEV
EATRNNLGWPYEPFKVPDEVKSHWSRHTPDGKALESDWNASFAAYEKKYPEEAAEL
KSIITGELPAGWEKALPTYTPESPGDATRNLSQQCLNALAKVVPGFLGGSADLASSNM
TLLKAFGDFQKATPEERNLRFGVREHGMGAICNGIALHSPGLIPYCATFFVFTDYMRG
AMRISALSEAGVIYVMTHDSIGLGEDGPTHQPVEHLASFRAMPNTLMFRPADGNETA
GAYKIAVTKRKTPSILALSRQKLPQLPGTSIEGVEKGGYTISDNSSGNKPDVILVGTGSE
LEIAAQAAEVLRKEGKTVRVVSFVCWELFDEQTDEYKESVLPSGVSARVSIEAASTFG
WGKIVGGEGKSIGINSFGASAPAPLLYKEFGITVEAVVDAAKSFF.

>Gene0342230.1

MGVGILASRAIRPASRLLRSQPSNLFLRTIVSKPELQSPEAAAVSQPEPPKNQILPPRNP
VGGARVHFTNPEDAIEVFVDGYAVKVPKGFTVLQACEVAGVDIPRFCYHSRLSIAGNC
RMCLVEVEKSPKPVASCAMPALPGMKIKTDTPIAKKAREGVMEFLLMNHPLDCPICD
QGGECDLQDQSMAFGSDRGRFTEMKRSVVDKNLGPLVKTVMTRCIQCTRCVRFASE
VAGVQDLGMLGRGSGEEIGTYVEKLMTSELSGNVIDICPVGALTSKPFAFKARNWEL
KGTETIDVSDAVGSNIRVDSRGPEVMRIIPRLNEDINEEWISDKTRFSYDGLKRQRLSD
PMIRDSDGRFKAVSWRDALAVVGDIIHQVKSDEIVGIAGQLSDAESMMVLKDFVNRM
GSDNVWCEGTAVGVDADLRYSYLMNSSISGLENADVFLLVGTQPRVEAAMVNARIC
KTVRASNAKVAYIGPPADFNYDCKHLGTGPDTLKEIAEGRHPFCAALKNAKNPAIIVG
AGLFNRTDKDAILAAVESIAQANNVVRPDWNGLNYLLLYAAQAAALDLGLIQQSAK
ALESAKFVYLMGADDVDVDKIPKDAFVVYQGHHGDKAVYRANVILPASAFTEKEGT
YENTEGFTQQTVPAVPTVGDARDDWKIVRALSEVSGVNLPYNSIEGVRSRIKSVAPNL
VHTDEREPAAFGPSLKPECKETMSTTPFKPVVENFYMTNAITRASKIMAQCSAVLLKK
.

>Gene0346820.1

MAMSILKLRNSPALRSAASSARIGVSSRAFSKLSEGTDITSAAPGVSLQKARSWDEGV

SSKFSTTPLSDIFKGKKVVIFGLPGAYTGVCSQQHVPSYKSHMDKFKAKGIDSIICVSV

NDPYTINGWAEKIGAKDAIEFYGDFDGKFHKSLGLDKDLSAALLGPRSERWSAYVED

GKVKAVNVEEAPSDFKVTGAEVILGQI.

>Gene0408850.1

MADAAKDLASGTVGGAAQLIVGHPFDTIKVKLQSQPAPSPGQPPRYTGAIDAVKQTV

ASEGPKGLYKGMGAPLATVAAFNAVLFTVRGQMEGLLRSEPGVPLTISQQFVCGAGA

GFAVSFLACPTELIKCRLQAQGAGGGASTTGSVVAAVKYGGPMDVARHVLRSEGGAR

GLFKGLFPTFAREVPGNATMFAAYEAFKRFLAGGSDTSSLGQGSLIMAGGVAGASFW

GFVYPTDVVKSVLQVDDYKNPKYKGSMDAFRKILKAEGVKGLYKGFGPAMARSVP

ANAACFLAYEMTRSSLG.

>Gene0358710.1

MATHSALAVSRIPVKPRLQSKSAIHSFPAQCSSKRLEVAAFSGLRVSSNGGEASFFDAIA

AQITPKAVGTSSPVRGETVAKLKVAINGFGRIGRNFLRCWHGRKDSPLEVVVLNDSGG

VKNASHLLKYDSMLGTFKADVKIVDNETISVDGKFIKVVSNRDPLKLPWAELGIDIVI

EGTGVFVDGPGAGKHIQAGASKVIITAPAKGADIPTYVVGVNEQDYSHDVANIISNAS

CTTNCLAPFAKVLDEEFGIVKGTMTTTHSYTGDQRLLDASHRDLRRARAAALNIVPT

STGAAKAVSLVLPQLKGKLNGIALRVPTPNVSVVDLVINVEKKGLTAEDVNEAFRKA

AAGPLKGVLEVCDTPLVSCDFRCSDVSTTIDSSLTMVMGDDMVKVVAWYDNEWGY

SQRVVDLAHLVAAKWPGEVAAGSGDPLEDFCKTNPADEECKVYEA.

>Gene0390270.1

MEETKENYDLTPRVAPNLDRHLVFPILEFLQERQLYPDEQILKFKIELLNKTNMVDYA

MDIHKSLYHTEDAPQDMVERRAEVVARLKSLEEAAAPLVTFLLNPNAVQELRADKQ

YNLQMLKERYQIGPDQIEALYQYAKFQFECGNYSGAADYLYQYRTLCSNLERSLSAL

WGKLASEILMQNWDIALEELNRVKEIIDSKSFASPLNQVQNRIWLMHWGLYIFFNHD

NGRTQIIDLFIQDKYMNAIQTSAPHLLRYLATAFIVNKRRRPQLKEFIKVIQQEHYSYK

DPIVEFLACVFVNYDFDGAQKKMKECEEVIVNDPFLGKRVDDGNFSTVPLKDEFLEN

ARLFIFETYCRIHQRIDMGVLAEKLNLNYEEAERWIVNLIRTSKLDAKIDSESGTVIME

PTQPNVHEQLVNHTKALSGRTYKLVTQLLEHSQGQAAR.

>Gene0468220.1

MAYSACFLHQSALASSAARSSSSSPSQRYVSLSKPVQIVCKAQQPHEDDNSAVSRRLA

LTLLVGAAAVGSKVSPADAAYGEAANVFGKPKKNTDFTPYSGDGFQVQVPAKWNPS
REVEYPGQVLRYEDNFDATSNLNVMVTPTDKKSITDYGSPEEFLSQVNYLLGKQAYF
GETASEVNFPCIYNVSDVGGKPYYYLSVLTRTADGDEGGKHQLITATVNGGKLYICKA
QAGDKRWFKGANKFVEKAATSFSVA.

>Gene0364770.1

MQTRNTFSWIREEITRSISVSLMIYIITWASISSAYPIFAQQNYENPREATGRIVCANCHL
ASKPVDIEVPQAVLPDTVFEAVVKIPYDMQLKQVLANGKKGALNVGAVLILPEGFEL
APPDRISPEMKEKIGNLSFQNYRPNKKNILVIGPVPGQKYSEITFPILAPDPATNKDVHF
LKYPIYVGGNRGRGQIYPDGSKSNNTVYNATAGGIISKILRKEKGGYEITIVDASNERQ
VIDIIPRGLELLVSEGESIKLDQPLTSNPNVGGFGQGDAEIVLQDPLRVQGLLFFLGSVV
LAQIFLVLKKKQFEKVQLSEMNF.

>Gene0366030.1

MALRMWASSTANALKLSSSASRSHLLPAFSISRCFSSVLEGLKYANSHEWVKHEGSVA
TIGISDHAQDHLGEVVFVELPEEKSSVTKEKNFGAVESVKATSEIISPISGEVIEVNTKL
ADSPGLINSSPYEDGWMIKVKPSNPAELESLMGPKEYTKFCEEEDAAH.

>Gene0376100.1

MKKLLEFGRKAMFYVRVLSGYEERRIRNYRLQLEKRIQQAQQRKAEINRLPEKVVLS
EVRRMVEQMQNLNKQIENTEAQIEDYFKPIDKQAGTIMEVQLESEKKTMGTMMNAT
QEETIRKIEEAERLARANATAETNMGEKIQDSESSANEKAQAK.

>Gene0404840.1

MNTALATTTATTPALRRETPPLRHCSLAKPSVFRLNRVGFKSGVQTIGKSLLRISASNQ
SASAAVNVATNASIPSEMKAWVYGEYGGVDVLKLESNIAVPEVNDDQVLIKVVAAAL
NPVDAKRRQGKFKATDSPLPTVPGYDVAGVVVKVGSAVKDFKEGDEVYANVSEKAL
EGPKQSGSLAEYTAVEEKLLALKPKNIDFAQAAGLPLAIETADEGLVRTEFSAGKSILV
LNGAGGVGSLAIQLAKHVYGASKVAATASTGKLELVRSLGADLAIDYTKENIEDLTEK
FDVVFDAIGMCDKAVKVIKEGGKVVALTGAVTPPGFRFVVTSNGDVLKKLNPYIESG
KVKPVVDPKGPFPFSRVADAFSYLETNHATGKVVVYPIP.

>Gene0380280.1

MGTLGRVIYTVGNWIRGSGQALDRVGSLLQGSHRLEEHLSRHRTLMSVFDKSPLVDK
DVFVAPSASVIGDVQIGKGSSIWYGCVLRGDVNNISVGSGTNIQDNSLVHVAKTNLAG

KVLPTTIGDNVTVGHSAVIHGCTVEDEAFVGMGATLLDGVVVEKHAMVAAGSLVRE
NTRIPSGEVWGGNPAKFMRKLTDEEVAYISKSAENYINLAHIHAAENSKSFEEIEVERA
LRKKYARKDEDYDSMLGIVREIPAELILPDNVLPEKTTTRVPTTHY.
>Gene0380400.1
MKMMIINIFFILSLVSSISFASVQDFCVADPSGPQGPSGFSCKNPDQVTANDFAFSGLAK
AGNTSNMIKAAVTTAFAPAFSGVNGLGISVVRLDLAAGGVVPLHIHRGASEVLIVIEG
TIRGGFISSDNKVYLKTLQKGEVIVFPQGLLHFALNNGTGPALAFAALGSSNPGVQLVP
SALFASDLPSELVEATTFLSREEIRRLKRVFGGSN.
>Gene0380450.1
MLRLSKRSVSSFLRSGDRSFRVSSAATSISRSSPSITDSKRGESESRWYSSLTNGKCMK
NDSLPHLNMKTNWFMGYRNESSAAASDSASQAPPPVEKYEYQAEVSRLMDLIVNSL
YSNKEVFLRELISNASDALDKLRYLSVTNPELSKDVADLDIRIYADKENGVITLTDSGI
GMTRQELVDCLGTIAQSGTAKFLKALKDNKDAGGDNNLIGQFGVGFYSAFLVADRVT
VSTKSPKSDKQYVWESEADSSSYTIKEETDPQLIIPRGTRITLHLKRDEKGYADPERIQ
KLVKNYSQFVSFPIYTWQEKGYTKEVEVDDDPAESKKDDQDDQTERKKKTKKVVER
YWDWELTNETQPIWLRNSKEVTTEEYNEFYRKTFNEYLDPLASSHFTTEGEVEFRSIL
YVPPVSPMGKDDVVNQKTKNIRLYVKRVFISDDFDGELFPRYLSFIKGVVDSHDLPLN
VSREILQESRIVRIMKKRLVKKAFDMILGISLSENREDYEKFWDNFGKHLKLGCIEDRE
NHKRLAPLLRFFSSQSENDMISLDEYVENMKAEQKAIYYIASDSVTSAKNAPFLEKLT
EKELEVLYLVEPIDEVAIQSLKSYKDKDFIDISKEDLDLGDKNEEKEAAVKKEFGQTCD
WIKKRLGDKVASVQISSRLSSSPCVLVSGKFGWSANMERLMKAQAGGDTTSLEFMK
GRRVFEINPDHSIIKNINAAYKSNPNDEDAMRAIDLMYDAALVSSGFTPENPAELGGKI
YEMMGIALSGKWSSPEVQPQQQHMAHSHNAELLEAEVVEPVEVDGKK.
>Gene0380560.1
MALVLHTYKGNKGAEKALIAAEYTGVKIDVPDFEMGVSNKTPEFLKMNPIGKVPVL
ETPEGPIFESNAIARYVSRLNGESSLNGSSLIEYAQIEQWIDFSSLEIFGSIFMWFGARIG
YKPYSVPGEEAAISALKRALDALNTHLASKTFLVGHSITLADIITVCNLSLGYTTVMT
KSFTSAFPHVERYFWTVINQPNFKKVVGDVKQTEAVPPVASKKPAQPAKAKEEPKKK
AAPAAEAPKPVEEEEAPKPKAKNPLDLLPPSPMVLDEWKRLYSNTKSNFREVAIKGF
WDMYDPEGYSLWFCDYKYNDENMVSFVTLNKVGGFLQRMDLARKYAFGKMLICG

SEGPFKVKGLWLFRGPEIPKFVMDEVYDMELYEWTKVDISDEAQKERVSQMIEDAEP
FEGEALLDAKCFNLFFQSSFKDKAAIVNDGGVRRRESWPLRLLVWSKSTCLIRSLIPFG
.
>Gene0381400.1
MAYASRFLSRSKQLQGSLGVLQQQHNAIPVRAFAKEAARPTFKGDEMLKGVFTEIKN
KFLAAVDILRKEKITLAPEDPAAVKQYANVMKTIRQKADMFSESQRIKYDIENETKEIP
DARAYLLKLKDIRTRRGLTDELGAEAMMFEALEKVEKDIKKPLLRSDKKGMDLLVAE
FEKGNKKLGIRKEDLPKYEENLELSIAKAQLDELKSDALEAMESQKKKEEFKDEEMP
DVKSLDIRNFM.
>Gene0384470.1
MIGQKKYPLTHKESLSLHLSFTTSFPFSPTMASTSSLALSQALLARAISLHGSDQRISLP
SSFSRASASSRRRNAASMTKLRSIRPLVRAAAVETLETTTDSSIIDKSVNSIRFLAIDAVE
KAKSGHPGLPMGCAPMAHILYDEVMRYNPKNPYWFNRDRFVLSAGHGCMLLYALL
HLAGYDSVLEEDLKSFRQWGSKTPGHPENFETPGIEVTTGPLGQGIANAVGLALAEK
HLAARFNKPDAEVVDHYTYVILGDGCQMEGISNEAASLAGHWGLGKLIAFYDDNHI
SIDGDTEIAFTENVDQRFEALGWHVIWVKNGNTGYDEIRAAIKEAKTVTDKPTLIKVT
TTIGYGSPNKANSYSVHGAALGEKEVEATRNNLGWPYEPFQVPEEVKSHWSRHTPEG
KALESDWNATFAAYEKKYPEEAAELKSIITGELPAGWEKALPTYTPESPGDATRNLSQ
QCLNAIAKVVPGFLGGSADLASSNMTLLKASGDFQKATPEERNLRFGVREHGMGAIC
NGIALHSPGLIPYCATFFVFTDYMRGAMRISALSEAGVIYVMTHDSIGLGEDGPTHQPI
EHIASFRAMPNTLMFRPADGNETAGAYKIAVTKRKTPSILALSRQKLPQLPGTSIEGVA
KGGYTISDDSTGNKPDVILIGTGSELEIAAQAAEVIRKEGKTVRVVSFVCWELFDEQT
DEYKESVLPSGVSARVSIEAASTFGWGKIVGGKGKSIGINSFGASAPAPLLYKEFGITV
EAVVDAAKSFF.
>Gene0385830.1
MLRRVIGNIAVRRQLQRALSSKAGGGSGKPSDVSAAVDSMLLRSLKEHYLEVSKMTP
PPKVTPPSPFEIVKGSLEGTGAVLRKSIGNEEINLFVMRLAHGGDDEDEDGINQLFLHV
AVSKPNQAESLHFLCGLYPDALGIHSVSMRPKLEDLELSDDPARYTGPSFEELDEKMR
DVFHSFLEERGVNESLFPFLQAWLYVKDHRNLLRWFKSVGTYVHETPSAENNA.
>Gene0393530.1

MLVYTDLLTGDELLSDSFPYKEIENGILWEVEGKWTTKGCVEVNIGANPSAEEGGED
EGVDDSVEKVVDIVDTFRLQEQPTYDKKGFIAYIKKYIKLLTPKLTPEQQEEFKKGIEG
ATKYLLPKLKDFQFFVGEGMHDDSTIVFAYYKEGATNPTFLYFAHGLKEVKC.
>Gene0395720.1
MAKMMMMQQSQPSLSLLTSSVSDFNGAKLHLQVQYKRKVQQPKGALYVSASSEKK
ILIMGGTRFIGIFLSRLLVKEGHQVTLFTRGKSPIAKQLPGESDQDFADFSSKILHLKGD
RKDYDFVKSSLSAEGFDVVYDINGREAEEVEPILDALPKLEQYIYCSSAGVYLKSDVL
PHCEVDAVDPKSRHKGKLETESLLQSKGVNWTSIRPVYIYGPLNYNPVEEWFFHRLK
AGRPIPVPNSGIQISQLGHVKDLATAFLAVLGNEKASREIFNISGEKYVTFDGLARACA
KAGGFPEPEIVHYNPKEFDFGKKKAFPFRDQHFFASVEKAKHVLGWKPEFDLVDGLT
DSYNLDFGRGTFRKEADFTTDDMILSKKLVLQ.
>Gene0399090.1
MHIADRDKCNWLRDKIETPTPRQYNSERRVVIYDRLTWSTQFENFLATKWTTAKRFG
LEGAESLIPGMKEMFDRAADLGVENIVIGMPHRGRLNVLGNVVRKPLRQIFSEFSGGT
RPVDEVGLYTGTGDVKYHLGTSYDRPTRGGKHLHLSLLANPSHLEAVDPVVMGKTR
AKQYYTKDESRTKNMGILIHGDGSFAGQGVVYETLHLSALPNYCTGGTVHIVVNNQV
AFTTDPRAGRSSQYCTDVAKALDAPIFHVNADDIEAVVHVCELAAEWRQTFHSDVVV
DLVCYRRFGHNEIDEPSFTQPKMYKVIRSHPSSLQIYQEKLVESGQVTKEDIDKIQKKV
SSILNEEFEASKEYIPQKRDWLASHWTGFKSPEQISRIRNTGVKPEILKNVGKAISTFPE
NFKPHRGVKRVYEQRAQMIESGEGIDWGLGEALAFATLVVEGNHVRLSGQDVERGT
FSHRHSVLHDQETGKEYCPLDHLTMNQDPEMFTVSNSSLSEFGVLGFELGYSMENPN
SLVIWEAQFGDFANGAQVMFDQFISSGEAKWLRQTGLVVLLPHGYDGQGPEHSSGRL
ERFLQMSDDNPFVIPEMDPTLRKQIQECNWQIVNVTTPANYFHVLRRQIHRDFRKPLI
VMAPKNLLRHKKCVSNLSEFDDVKGHPGFDKQGTRFKRLIKDQSGHSDLEEGIRRLV
LCSGKVYYELDEERQKSGTNDIAICRMEQLCPFPYDLIQRELKRYPNAEIVWCQEEPM
NMGAYQYIAPRLCTAMKALNRGSFNDIKYVGRLPSAATATGFYQLHVKEQTDLVHK
ALQPDPITPILP.
>Gene0496440.1
MAMANLARRKAYFLTRNVTTSHTDALRFSFSLSRGFASSGSDENDVVVIGGGPGGYV
AAIKAAQLGLKTTCIEKRGALGGTCLNVGCIPSKALLHSSHMYHEAKHAFAHHGIKL

ASVEVDLPAMLAQKDNAVKNLTRGIEGLFKKNKVTYVKGYGKFLSPNEVSVETIDGD
NTVVKGKHIIVATGSDVKSLPGITIDEKKIVSSTGALSLSEVPKKLIVIGAGYIGLEMGS
VWGRLGSEVTVVEFAGDIVPSMDGEIRKQFQRSLEKQKMKFMLKTKVVSVDASGDG
VKLTVEPSEGGDQTTLEADVVLVSAGRTPFTSGLDLEKIGVETDKGGRILVNERFSTN
VSGVYAIGDVIPGPMLAHKAEEDGVACVEFIAGKHGHVDYDKVPGVVYTHPEVASV
GKTEEQLKKDGVSYRVGKFPFMANSRAKAIDNAEGLVKILADKETDKILGVHIMSPN
AGELIHEAVLAINYDASSEDIARVCHAHPTMSEALKEAAMATYDKPIHI.

>Gene0399970.1

MAMASLARRKAYLLTRNISTAPLRSSFSLSRGFASSGSDENDVVIIGGGPGGYVAAIKA
AQLGLKTTCIEKRGALGGTCLNVGCIPSKALLHSSHMYHEAKHVFANHGVKVSSVE
VDLPAMLAQKDTAVKNLTRGIEGLFKKNKVNYVKGYGKFLSPSEVSVETTDGETTVV
KGKHIIVATGSDVKSLPGITIDEKKIVSSTGALSLSEIPKKLIVIGAGYIGLEMGSVWGR
LGSEVTVVEFAADIVPSMDGEIRKQFQRSLEKQKMKFMLKTKVVGVDSSGDGVKLIV
EPAEGGDQTTLEADVVLVSAGRSPFTSGLDLDKIGVETDKVGRILVNERFSTNVSGVY
AIGDVIPGPMLAHKAEEDGVACVEFIAGKHGHVDYDKVPGVVYTYPEVASVGKTEE
QLKKDGVSYRVGKFPFMANSRAKAIDTAEGLVKILADKETDKILGVHIMSPNAGELIH
EAVLAINYDAASEDIARVCHAHPTMSEAIKEAAMATYDKPIHM.

>Gene0402240.1

MNALAATNRNFRLASRLLGLDSKLEKSLLIPFREIKVECTIPKDDGTIASFVGFRVQHD
NARGPMKGGIRYHPEVEPDEVNALAQLMTWKTAVANIPYGGAKGGIGCDPSKLSISE
LERLTRVFTQKIHDLIGIHTDVPAPDMGTGPQTMAWILDEYSKFHGYSPAVVTGKPIDL
GGSLGRDAATGRGVMFGTEALLNEHGKSISGQRFVIQGFGNVGSWAAKLISEQGGKI
VAVSDITGAIKNKDGIDIESLLNYTKEHRGVKGFDGAHPIDANSILVEDCDILIPAALGG
VINRMRMRLKQSSSLKLLTIQLILMLMSKKGVVILPDIYANSGGVTVSYFEWVQNIQG
FMWEEEKVNDELKTYMNRGFKDMKEMCKTHSCDLRMGAFTLGVNRVARATVLRG
WGA.

>Gene0402300.1

MATANALSSPSVLCSSRQGKLSGGSQQKGQRVSYRKANRRFSVRANVKEIAFDQSSR
AALQAGIDKLADAVGLTLGPRGRNVVLDEFGSPKVVNDGVTIARAIELPDAMENAGA
ALIREVASKTNDSAGDGTTTASVLAREIIKHGLLSVTSGANPVSLKRGIDKTVQALIEE

LEKRARPVKGGSDIKAVATISAGNDELIGAMIADAIDKVGPDGVLSIESSSSFETTVEVE
EGMEIDRGYISPQFVTNPEKLLVEFENARVLITDQKITAIKDIIPILEKTTQLRAPLLIIAE
DVTGEALATLVVNKLRGVLNVVAVKAPGFGERRKAMLQDIAILTGAEYQALDMGLL
VENTTIDQLGIARKVTISKDSTTLIADAASKDELQARISQLKKELFETDSVYDSEKLAE
RIAKLSGGVAVIKVGAATETELEDRKLRIEDAKNATFAAIEEGIVPGGGATLVHLSTVIP
AIKETFEDADERLGADIVQKALVAPAALIAQNAGIEGEVVVEKIMFSEWELGYNAMT
DTYENLLEAGVIDPAKVTRCALQNAASVAGMVLTTQAIVVDKPKPKAPAAAAPEGL
MV.

>Gene0410190.1

MAMTTTTLFHALVPSNTYKPGAVSSSFVSVPRSSTPPSSPSLQFRSLVSDTTSIYGRRKF
TGNLRRVSLTVSAAAAAEPLTVLVTGAGGRTGQIVYKKLKERADQFVARGLVRTKES
KEKIGGEDEVFVGDIRDPEAIAPAVQGIDALVILTSAVPKMKPGFDPSKGERPEFYFEEG
AYPEQVDWIGQKNQIDAAKAAGVKQIVLVGSMGGTNINNPLNSIGNANILVWKRKA
EQYLADSGIPYTIIRAGGLQDKEGGIRELIVGKDDEILETEMRTIARADVAEVCVQALQ
LEEAKFKALDLASKPEGTGTPTKDFKALFAQVTARF.

>Gene0410990.1

MALEVCVKAAVGAPDALGDCPFSQRVLLTLEEKNLPYKMHLINISDKPQWFLAISPEG
KVPVLKNDDKWVSDSDVITGILEEKYPEPSLKTPPEFASVGSKIFGTFVTFLKSKDSSD
GSEKALLDELEALETHLKTHDGPFIAGGKVSAVDLSLAPKLYHLKVALGHYKSWSVP
ESLPHVHGYMKALFSLDSFEKTKTEERYVIAGWEHKVNP.

>Gene0414230.1

MAATALSTASSILSSSSPPLTSAHPFLSRPTTLEFPSRFGLSSSSSTLTHRATHLRPLAAVE
APEKIEKIGSEISSLTLEEARILVDYLQDKFGVSPLSLAPAAAAVAAPGDGGGAAAVVE
EQTEFDVVINEVPSSSRIAVIKAVRALTSLALKEAKELIEGLPKKFKEGVTKDEAEEAK
KQLEEAGAKVSIA.

>Gene0425710.1

MSSYLIFTFIILLCLLYSSFSSSASLHNATNESVTFRPQHEIQKLKLIREHLQKINKPAIKT
IQSSDGDIIDCVPSHHQPAFDHPLLQGQRPMDPPEMPKGQSQENESHEDFQLWSLTGE
FCPEGTIPIRRTTEQDMLRASSVRKFGRKIRRVRRDSSSNGHEHAVGYVSGSKYYGAK
ANVNVWTPHVSSKYEFSLSQIWVIAGSFADDLNTIEAGWQVSPELYGDTNPRFFTYW

TSDAYQATGCYNLLCSGFIQTNNRIAIGAAISPVSSYKGGQFDISLLIWKDPKHGHWW
LQFGSGTLVGYWPVSLFTHLMEHGNMVQFGGEIVNTQPDGSHTSTQMGSGHFAGEG
FGKASYFRNLEVVDWDNTLIPISNLRVLADHPNCYDIRGGVNRVWALQLAELLVSSV
VHILFGLYLFSSAIAGDLTQTFLESIFKPKPIVEVKQGNTTTQVNDLTPIVLVHGIFGFGK
GRLGGLSYFAGAEKKDERVLVPDLGSLTSVHDRARELFYYLKGGRVDYGEDHSKAC
GHSQFGRFYDKGEYQEWDEDHPIHFVGHSAGAQVVRVLQQMLADEMFDGHENTNE
NWVLSLTSLSGALNGSTRTYLDGIQPEDGKSLKPICLLQICRFGSIIYDWLDISWLKSY
YNIGFDHFNMSWKKTGLRGLVDCLLGNAGPFASSSGDWILPDLSIQETMKLNANLKT
FPNTFYFSYATKRTRKPPLGGVMGIHPLLSIRVLQMSQWRYPHEDWQDNDGALNTVS
MTHPRIPVEHSNLVVRSDSDCLPLQPGIWYYKIVEADHIMFILNRERAGEMALEYDLL
NENVKKCQYAVRGELYLRASELQKEGKKIIFTNVGNPHALGQKPLTFPRQVVALCQA
PFLLDDPNVGMLFPADAIARAKHYLSLTSGGLGAYSDSRGLPGVRKEVAEFIQRRDGY
PSDPELIFLTDGASKGVMQILNCVIRGEGDGILVPVPQYPLYSATISLLGGSLVPYYLDE
SENWGLDVNNLRQSVAQARSQGISVRAMVIINPGNPTGQCLSEANLKEILKFCYNEKL
VLLGDEVYQQNIYQDERPFISSKKVLMDMGSPFSKGVQLVSFHTVSKGYWGECGQR
GGYFEMTNIPPRVVEEIYKVASIALSPNVSAQIFMGLMVSPPKPGDISYDQFARESKGIL
ESLRRRAKIMTDGFNSCKNVVCNFTEGAMYSFPQIRLPPGALQAAKQAGKVPDVFYC
LKLLEATGISTVPGSGFGQKEGVFHLRTTILPAEEEMPEIMDSFKKFNDEFMAQYENGF
GYSRM.

>Gene0432050.1

MVLLLLLRRAAIARTSSLLRARLLTPVSGFHTRSISFSAPPPPPLTFFLEFLLRFGLQNFSS
TELHLTHFPLMFVDGRCNRSYKILPLILFSHGNIAKWLKKEGDKVEVGDVLCEIETDK
ATVELESQEEGFLAKIVVTEGSKDIPVNAPIAIMVEEEDDIQNVSAVEGGQVGKEETSA
PQEMKSEESTQQRDSIQPDASDLPPHVVLEMPALSPTMIEVGDVIGEIETDKATLEFDS
LEEGYLAKILIPEGSKDVAVGKPIALIVEDAESIEAIKSSSAGGSEAVTEKQAPQSVADK
SGEKKAGFTKISPAAKLLILEHGLEASSIEASGPYGTLLKSDVVAAIASGKTSKKSVSTE
KKQPSKENISKSSSVSRPESKSSLTPSNDYEDFPNSQIRKIIAKRLLESKQKIPHLYLSSD
VVLDPLLAFRKELQENHGVKVSVNDIVIKAVAVALRNVRQANAFWDAEKGEIVMLE
DVDISIAVATEKGLMTPIIRNADQKSISAISLEVKELAQKARSGKLAPHEFQGGIFSISNL
GMYPVDQFCAIINPPQAGILAVGRGNKVVEACIGADGVEKPSVITKMNVTLSADHRIF

DGQVGASFLSELRSNFEDVRRLLL.

>Gene0532070.1

APVRGILGLQRAVSVWKESNRLAPALRSFSTQAASTSTTPQPPPPPPPPEKTHFGGLKD

EDRIFTNLYGLHDPFLKGAMKRGDWHRTKDLVLKGTDWIVNEMKKSGLRGRGGAG

FPSGLKWSFMPKVSDGRPSYLVVNADESEPGTCKDREIMRHDPHKLLEGCLIAGVGM

RASAAYIYIRGEYVNERLNLEKARREAYAAGLLGKNACGSGYDFDVYIHFGAGAYIC

GEETALLESLEGKQGKPRLKPPFPANAGLYGCPTTVTNVETVAVSPTILRRGPEWFSSF

GRKNNSGTKLFCISGHVNKPCTVEEEMSIPLKELIERHCGGVRGGWDNLLAIIPGGSS

VPLIPKNICEDVLMDFDALKAVQSGLGTAAVIVMDKSTDVVDAIARLSYFYKHESCG

QCTPCREGTGWLWMIMERMKVGNAKLEEIDMLHEVTKQIEGHTICALGDAAAWPV

QGLIRHFRPELERRIRERAERELLQAAA.

>Gene0441590.1

MSHSVKIYDTCIGCTQCVRACPTDVLEMIPWDGCKAKQIASAPRTEDCVGCKRCESA

CPTDFLSVRVYLWHETTRSMGLAY.

>Gene0449980.1

MDFHAMNRKNLQILCKKHGIPANLKNVEMANRLSSLIIQKEEEEEEVVASRKAKKVR

FSPETDNQVFEFTRSVKKSVRTRKAPQAGGGIELRRSKRIGSIGSVQEEGLLPGDRDIQ

DERRSTRLAARIEKACVEGGTSKAVALLPAAKRSKRSGSGGSSTQEEGEDNDLNAPER

VEDRDVQGGRRSRRLAAKTEKSSEEGGMSKSVTLLPAAKRSKGLVDVANKEDERET

GEPNRKGGGSKVEMVRRRSMRFVNEQTSAQDQRRSVRLKASVEKTLVGQAKNDSV

KASRVVKGNLVDKKTDENLVKSKRVTRNMKRGRSGEPEVESGAASNQSNLTLKKTL

NEFAHFEQEEACGADVKAGGSSKNQKCIEDKPQGIIIIEDSPSSSKTKAAESVEKVFDP

TLDKSADSSQRSNSREINCESVEGDCEEKLERETVSMPVMEEDKEEVSPRSLSSPKDK

LHVPTGHIIVQDIASTFIAEESTKTKDKTLIYSPESELKENSCIAKLANVEESLENSTER

WKEIHSGKDDEKGSLENDVQAENLHGNVSECNTESSSAEEEMEISKIGGLSVAHCVN

LIPEKFVGEYSQLEPEEAERPNVEARSSCQKVKKIVAQEFVKDKPQEMAEDSPSTSET

KATEPTVISENVLDSTRTVSGETSAVRNSHELNSEVLEEGREEKHEQAKTKKDKGETS

SLSEFLTERSEVKTCLDNRITSCSLSVEATLSPASVQLAMSNPEADLGVPTGNEEETLIL

TPTSELKEDNAVAKISKVEAILGNSAECCKEDEKGSLEKDVQAENLHVNFSECNTAKS

SSEEVEISKDGSMSVNLTPEKLLDTYTQLVPEEAGGPNVEIRSSSKKMKIVSSECPREN

PQGMAEESPSTFVTKTAETLMMSENFSVDISPVGNTQELNHELRDEEREEKQELDIVL
VAETEKEKKKASSPSELFVETTPPPPSLVQIAVSNPESELSVPTGHILGKDIVSAVIAEEA
IKTEEVSKSVQSFVAKFAETDVLENSAECSSKSLSSKDDGRGSLEKEKQSAKLHGNFS
EYNSENISAEEQADICKVGRISPGHCVHRETLDEVEDESLMKSVQTISSARGCKPNAL
ELSGSFSTDFASLSHKKENVSDCLEEEEMKALSQPIPIQKAASNVLERSSLFTTPERNL
MLMEQQSESGKICEADIVTQHNDEAVESHAVVFTTPEKVLLLGDSWLDDVGKEGEHT
ARDFPDESDVLNTSANEAFNEGERIVVELHDESNIAASPLRHSRVGNFKEERTERNEE
KRTVELHFESGTFTGPDKHDGAENSEGNKAMELYEESVDFTGLEERHELFRDSGKDK
AREDELKMDAQFYEEAGLSTEMHKDLPLADPELGEAGWLGINNDDKSGSLEGKLLY
GDSEQKKAEKAPAEFHDESAVPSIPERHPFPEESELEEAAKSEENNALESQADCDNFT
DAIVNAKSHDMSDVLTAPESHSIMGAFEPDGEENKDVELLGESNISTNEESGQDGKIK
YNTAATCEESSFFNSPERRHHLGNTGPHTAGKQERKEVEFKDESAFFTRLETRLLLGE
STQDRLDNGKSGSAKYQSHRDSSRKMILKDDSVARECQVAAPDFRENTIADFSGSIAS
KVSYSHEFSAGEVSAGAEFMPKASQVENVAGLDATQGTSKQSRGNSPNVDTCHSME
ADTIMDAGRNVSFSSYVLSLPAEGNSETIGEISNHIEVAGTCSLVSEESGPSTDIQNQIH
DAVEELAVTDEIVDAKLIKNSQSDSKTISLPAEGDSKSIGENSSQLEATGTCSMVSEKSI
PSMDIQNHINDALEEELAVTDNSKADELIEAKVTKNVDSSDGSGKTCVLTGTEDEHLC
YNSEVADPVDTAFEKDISLTPDESTLQKNKNQEEISADDEMLKREDTPSPAETFNESSE
NIGESSEKQVRREQVLFYRTQAKPKTHDMKENAPNSKIVDNLNVTAPRTSKRQPLQD
LRKN.

>Gene0450290.1

MAYASRILNHSKKLKDVSTLLRREHAASIRYFSSTNRAPPLSREDASRARLGFSPVER
MTKCSTDIVPVSISFATTRTTLSSTVGRPRLGQEFSCSMQSVRGFSSGSDLPPHQEIGMP
SLSPTMTEGNIAKWLKKEGDRVAPGEVLCEVETDKATVEMECMEEGFLAKIVKAEGS
KEIQVGEVIAITVEDEEDIGKFKDYTPSSTTDATPPKEEPAPPPPKEEKVEQASPPPEPKT
SKPSPPPSGDRVFASPLARKLAEDNNVPLSSIKGTGPEGRIVKADIEDYLASGGKEATA
KQSKVTDSKVPALDYVDIPHSQIRKVTASRLAFSKQTIPHYYLTVDACVDKLMGLRN
QLNSFQEASGGKRISVNDLVVKAAALALRKVPQCNSSWTDDYIRQFKNVNINVAVQT
ENGLYVPVVKDADKKGLSTIGEEVRLLAQKAKENTLKPEDYEGGTFTVSNLGGPFGI
KQFCAVVNPPQAAILAVGSAEKRVVPGNGPDEYNFASYMPVTLSCDHRVVDGAIGAE

WLKAFKGYIENPESMLL.

>Gene0454250.1

MEAHVLPNLPHEIVCKIIELVGEESFYNLGPFLRAGKRGYALAHEPSVLKKCDVSEME

DRFVTCQIRQGCQFREFHLKCVSAGNRKAIYYDGLLTAPSIGLEESIKILEPNRAKCFS

CSQHYHHDLRSDETSEMGESIENQLKAFGAEDPNCNKYGESFKFPDDGLIKTPSKIPS

MSTSSSSDGASYVDMLYAVNDSNFGIPDRCRCGSAIIIQISTEAAAIPKKYFVCNDFKN

DGLHRKQEWTAAIEDETRRLKKTVDDHESRIRILGRVEYRIDRIDQDAQKNDGEIAHL

GYQIHEMEKVLKKNAEEIALLKEIIEKL.

>Gene0455000.1

MFRFVSSLASKARIASNTRQVSSRMSWSRNYAAKEIKFGVEARALMLRGVEELADAV

RVTMGPKGRTVVIEQSWGAPKVTKDGVTVAKSIEFKDKVKNVGASLVKQVANATND

VAGDGTTCATVLTRAIFTEGCKSVAAGMNAMDLRRGISMAVDAVVTNLKSRARMIST

SEEIAQVGTISANGEREIGELIAKAMEKVGKEGVITIQDGKTLINELEVVEGMKLDRG

YTSPYFITNQKTQKCELEEPLILIHEKKISSINSIVKVLELAMKKQRPLLIVSEDVESEAL

ATLILNKLRAGIKVCAIKAPGFGENRKANLQDLAALTGGEVITDELGMNLEKVDLGM

LGTCKRVTVSKDDTVILDGAGDKTAIEERCEQIRSAIELSTSDYDKEKLQERLAKLSG

GVAVLKIGGASEAEVGEKKDRVTDALNATKAAVEEGILPGGGVALLYAARELEKLPTA

NFDQKIGVQIIQNALKTPVHTIASNAGVEGAVIVGKLLEQDNTDLGYDAAKGEYVDM

VKAGIIDPLKVIRTALVDAASVSSLLTTTEAVVVDLPKDESESAGAGGMGGMGGMDY.

>Gene0458560.1

MAIRSLASRRTLAGLKETSSRLLGLRSIQTFTLPDLPYDYSALEPAISGEIMQIHHQKHH

QAYVTNYNNALEQLDQAVNKGDASTVVKLQSAIKFNGGGHVNHSIFWKNLAPVKE

GGGEPPKGALGGAIDTHFGSLEGLVKKMSAEGAALQGSGWVWLGLDKELKKLVVD

TTANQDPLVTKGGSLVPLVGIDVWEHAYYLQYKNVRPEYLKNVWKVINWKYASEVY

EKECK.

>Gene0500980.1

MALVVEKTSSGREYKVKDMSQADFGRLELELAEVEMPGLMACRTEFGPSQPFKGAR

ITGSLHMTIQTAVLIETLTALGAEVRWCSCNIFSTQDHAAAAIARDSAAVFAWKGETLQ

EYWWCTERALDWGPGGGPDLIVDDGGDATLLIHEGVKAEEIFEKTGQVPDPTSTDNP

EFQIVLSIIKEGLQVDPKKYHKMKERLVGVSEETTTGVKRLYQMQETGALLFPAINVN

DSVTKSKFDNLYGCRHSLPDGLMRATDVMIAGKVVVVCGYGDVGKGCAAAMKTA
GARVIVTEIDPICALQAMMEGLQVLTLEDVVSEADIFVTTTGNKDIIMVDHMRKMKN
NAIVCNIGHFDNEIDMLGLETFPGVKRITIKPQTDRWVFPDTKSGIIVLAEGRLMNLGC
ATGHPSFVMSCSFTNQVIAQLELWNEKSSGKYEKKVYVLPKHLDEKVAALHLGKLG
AKLTKLTKDQSDYVSIPIEGPYKPAHYRY.

>Gene0474760.1

MASTFTATSSIGSMVAPNGHKSDKKLMNKLSSSSFGRRQNVCPRLRRSSPAIVCAAKE
LHFNKDGTTIRRLQAGVNKLADLVGVTLGPKGRNVVLESKYGSPRIVNDGVTVAREV
ELEDPVENIGAKLVRQAAAKTNDLAGDGTTTSVVLAQGFIAEGVKVVAAGANPVLIT
RGIEKTAKALVAELKKMSKEVEDSELADVAAVSAGNNEEIGSMIAEAMSRVGRKGVV
TLEEGKSAENALYVVEGMQFDRGYISPYFVTDSEKMSVEFDNCKLLLVDKKITNARD
LVGVLEDAIRGGYPILIIAEDIEQEALATLVVNKLRGTLKIAALRAPGFGERKSQYLDDI
AILTGATVIREEVGLSLDKAGKEVLGHAAKVVLTKETSTIVGDGSTQDAVQKRVTQIK
NLIEQAEQDYEKEKLNERIAKLSGGVAVIQVGAQTETELKEKKLRVEDALNATKAAVE
EGIVVGGGCTLLRLASKVDAIKATLENDEEKVGADIVKRALSYPLKLIAKNAGVNGS
VVSEKVLSNENVKYGYNAATGKYEDLMAAGIIDPTKVVRCCLEHAASVAKTFLMSD
CVVVEIKEPEPVPAGNPMDNSGYGY.

>Gene0478940.1

MAIRSVASRRTLSSLKESSSKLLGLRGIQTFTLPDLPYDYSALEPAISGEIMQIHHQKHH
QTYVTNYNNALEQLDQAVNKGDASTVVKLHSAIKFNGGGAFLTFSSFISDLEVVVSC
EPFDFLEESCSCQCEFQGGGEPPKGALGGAIDTHFGSLEGLVKKMSAEGAALQGSGW
VWLGLDKELKKLVVDTTANQDPLVTKGGSLVPLVGIDVWEHAYYLQYKNVRPDYLK
NVWKVINWKYASEVYESECK.

>Gene0483570.1

MSKRGRGGTSGNKFRMSLGLPVAATVNCADNTGAKNLYIISVKGIKGRLNRLPSACV
GDMVMATVKKGKPDLRKKVLPAVIVRQRKPWRRKDGVFMYFEDNAGVIVNPKGEM
KGSAITGPIGKECADLWPRIASAANAIV.

>Gene0493780.1

MAPQEDAMQKQSSNNNSDVIFRSKLPDIYIPNHLPLHDYIFQNISEFASKPCLINGPTG
HVYTYSDVHVASRRIAAGFQKLGVNKNDVVMILLSNCPEFVLSFLAASFRGATATAA

NPFFTPAEIAKQAKASNSKLIVTESRYVDKIKDLQNDGVIIVCTDEEPSPIPEGCLRFTEL
TQSTEIETVEISSDDVVALPYSSGTTGLPKGVMLTHRGLVTSVAQQVDGDNPNLYFHS
DDVILCVLPLFHIYALNSIMLCGLRVGASILIMPKFEINLLLELIQRCKVTVAPMVPPIVL
AMAKSPETEKYDLSSIRVVKSGAAPLGKELEDAVSAKFPNAKLGQGYGMTEAGPVL
AMSLGFAKEPFPVKSGACGTVVRNAEMKIIDPDTGDSLSKNKPGEICIRGHQIMKGYL
NNPAATAETIDKDGWLHTGDIGLIDDDDELFIVDRLKELIKYKGFQVAPAELEALLIGH
QDITDVAVVAMKEEAAGEVPVAFVVKSKDSELSEDDVKQFVAKQVVFYKRINKVFFV
ESIPKAPSGKILRKDLRAKLANGLV.
>Gene0496330.1
MAQILAASPTCQMRLTKPSSIASSKLWNSVVLKQKKQSSSKVRSFKVMALQSDNSTIN
RVESLLNLDTKPFTDRIIAEYIWIGGSGIDLRSKSRTLEKPVEDPSELPKWNYDGSSTG
QAPGEDSEVILYPQAIFRDPFRGGNNILVICDTYTPAGEPIPTNKRARAAEIFSNKKVNE
EIPWFGIEQEYTLLQPNVNWPLGWPVGAYPGPQGPYYCGVGAEKSWGRDISDAHYK
ACLYAGINISGTNGEVMPGQWEFQVGPSVGIEAGDHVWCARYLLERITEQAGVVLTL
DPKPIEGDWNGAGCHTNYSTKSMREDGGFEVIKKAILNLSLRHMEHISAYGEGNERR
LTGKHETASIDQFSWGVANRGCSIRVGRDTEKKGKGYLEDRRPASNMDPYIVTSLLAE
TTLLWEPTLEAEALAAQKLSLKV.
>Gene0500950.1
MAGLMKLACLVLACMIVAGPITSNAALSCGTVSGYVAPCIGYLAQGAPALPRACCSG
VTSLNNLARTTPDRQQACRCLVGAANAFPTLNAARAAGLPKACGVNIPYKISKTTNC
NSVK.
>Gene0508280.1
MAMKSLSPVPKLLLSTTPSSVLSSDKNFFFVDFVGLYCKSKRTRRRLRGDSSSSTSRAS
HLSRLSSVRAVIDLERVHDKDLSSPSYLKPQVANLEDILSERGACGVGFIANLDNIPSH
GVVKDALIALGCMEHRGGCGADNDSGDGSGLMSSIPWDFFNVWAKEQGLAPFDKL
HTGVGMIFLPQEDTFMQEAKQVIENIFEKEGLEVLGWRDVPVNAPIVGKNARETMPN
IQQVFVKIAKDDSTDDIERELYICRKLIERAVAAETWGTELYFCSLSNQTIVYKGMLRS
EALGLFYLDLQNELYTSPFAIYHRRYSTNTSPRWPLAQPMRFLGHNGEINTIQGNLNW
MQSREASLKSSVWNGRENEIRPFGNPRGSDSANLDSAAEILIRSGRTAEEALMILVPEA
YKNHPTLSIKYPEVLDFYDYYKGQMEAWDGPALLLFSDGKTVGACLDRNGLRPARY

WKTSDNFVYVASEVGVVPVDEAKVTMKGRLGPGMMIAADLVNGQVYENTEVKKR
VSSLNPYGKWIKENLRFLKPVNFKSSTVMENEEILRTQQAFGYSSEDVQMVIESMAS
QGKEPTFCMGDDIPLAGLSQRPHMLYDYFKQRFAQVTNPAIDPLREGLVMSLEVNIG
KRGNILELGPENASQVILSNPVLNEGGIEELMKDTYLKPKVLSTFFDIRKGVEGSLQK
ALYYLCEAADDAVRSGSQLLILSDRTDSLEPTRPAIPIMLAVGAVHQHLIQNGLRMSAS
IVADTAQCFSTHQFACLIGYGASAVCPYLALETCRQWRLSNKTVALMRNGKIPTVTIE
QAQKNYTKAVNAGLLKILSKMGISLLSSYCGAQIFEIYGLGQEVVDLAFTGSVSKISGL
TFDELARETLSFWVKAFSEDTTKRLENFGFIQFRPGGEYHSNNPEMSKLLHKAVREKS
ETAYAVYQQHLANRPVNVLRDLLEFKSDRAPIPVGKVEPAVSIVQRFCTGGMSLGAIS
RETHEAIAIAMNRIGGKSNSGEGGEDPIRWKPLTDVVDGYSPTLPHLKGLQNGDIATS
AIKQVASGRFGVTPTFLVNADQLEIKVAQGAKPGEGGQLPGKKVSAYIARLRSSKPGV
PLISPPPHHDIYSIEDLAQLIFDLHQINPNAKVSVKLVAEAGIGTVASGVAKGNADIIQIS
GHDGGTGASPISSIKHAGGPWELGLTETHQTLIENGLRERVILRVDGGLKSGVDVLMA
AAMGADEYGFGSLAMIATGCVMARICHTNNCPVGVASQREELRARFPGVPGDLVNY
FLYVAEEVRGILAQLGYSKLDDIIGRTELLKPRDISLVKTQHLDLSYLLSSVGVPSMSST
EIRKQEVHTNGPVLDDDILEDPLVKDAIENEKVVDKTVKICNIDRAACGRVAGVIAKK
YGDTGFAGQVNLTFLGSAGQSFGCFLIPGMNIRLVGEANDYVGKGMAGGEIVVTPVD
KIGFVPEEATIVGNTCLYGATGGQIFARGKAGERFAVRNSLAEAVVEGTGDHCCEYMT
GGCVVVLGKVGRNVAAGMTGGLAYLLDEDDTLLPKINREIVKIQRVTAPAGELQLKS
LIEAHVEKTGSSKGETILKEWEKYLPLFWQLVPPSEEDTPEASAAYVRTATGEVTFQSA
.
>Gene0508920.1
APKRGVKVVAKKKTEKVTNPLFERRPKQFGIGGALPPKKDLTRYIKWPKSIRLQRQK
RILKQRLKVPPALNQFTKTLDKNLATQLFKVLMKYRPEDKAAKKDRLLKKAQAEAE
GKPSESKKPIVVKYGLNHVTYLIEQNKAQLVVIAHDVDPIELVVWLPALCRKMEVPY
CIVKGKSRLGTVVHQKTAACLCLTTVKNEDKLEFSKILEAIKANFNDKYEEYRKKWG
GGIMGSKSQAKTKAKERCKLYLYSIEDFSFLTLAFCRVFCSRPMEEDTNGKTEEEEFNT
GPLSVLMMSVKNNTQVLINCRNNRKLLGRVRAFDRHCNMVLENVREMWTEVPKTG
KGKKKALPVNRDRFISKMFLRGDSVIIVLRNPK.
>Gene0512900.1

MEEDEDMFLSSLGVTSANPADIEQTILDEATKKLDNDESVEERLEGSNLLPSSQSELLN
KLRAVKFEIDAVASTVEQAEEEDGLQSGSVLQNALAKDRLRSLRKRKNELEKELSGL
HGQGGTSGGADRGDILRDLVKGEPSSRKRKLKEVRKPSKREGKKVKVVSFHEDTDF
DAVFDAASAGFVETERDELVRKGILTPFHKLEGFERRLQQPGPSNTRNLPEGEDDNED
SSSIDRAVQSMSLAAKARPTTKLLDAHDLPKLEATPVPFRRLRKLYKTNDSSDSDAKK
SKGGKGKKKRPLPKRKWTKRISNEDSSLQENEDGRRISATSSCEEEELDDFDEVDDSE
KSSVPLEGGLNIPEGIFIKLFDYQRVGVQWLWELHCQKAGGIIGDEMGLGKTVQVLSF
LGSLHFSKMYKPSIVICPVTLLRQWRREARTWYPDFHVEILHDSAKDSNGKGRVDAS
ESDYDSEVSADGDHEQKSKNTKKWSSLINRVLNSDSGLLITTYEQLRLHGEKLLNIEW
GYAVLDEGHRIRNPNAEITLVCKQLQTVHRIIMTGAPIQNKLTELWSLFDFVFPGKLGV
LPVFEAEFSVPITVGGYANASPLQVSTAYRCAVVLRDLIMPYLLRRMKADVNAHLTK
KTEHVLFCSLTVEQRSTYRAFLASSEVEDIFDGNKNSLYGIDVMRKICNHPDLLEREHS
HQNPDYGNPERSGKMKVVAEVLKVWKQQGHRVLLFSQTQQMLDILESFLVANEYSY
RRMDGLTPVKQRMALIDEFNNSDDVFVFVLTTKVGGLGTNLTGANRVIIFDPDWNPS
NDMQARERAWRIGQKKDVTVYRLITRGTIEEKVYHRQIYKHFLTNRILKNPQQRRFF
KARDMKDLFILNDDGDSNASTETSNIFSQLSEDINIVGAQTENTTDSTTQLDTHDAAE
ELSGEKDAETNGEPVDEETNILKSLFDAHGIHSAVNHDAIINANDEEEKMRLEHQASQ
VAQRAAEALRQSRMLRSRESISVPTWTGRSGCAGAPSSVRRRFGSTVNSRLTTADKSS
AVKNGISAGLSSGKAPSSAELLNKIRGSREQAIGVGLEQTQTSSSSSSRVGSLQPEVLIR
QICSFVQRKGGSTDTSSIVNHFSDRVPAKDVPLFKSLLKEIATLRKDPNGSVWVLKSEY
KD.
>Gene0529200.1
MASLAQHFTGLRCSPLSSSSRLTRRAAKNFPQNKSSSVSPTIVAAVAMSSGQTRERLEL
KKMFEDAYERCRTAPMEGVAFTVDDFAAAIEQYDFNSEIGTRVKGTVFKTDANGALV
DISAKSSAYLSVEQACIHRIKHVEEAGIVPGMVEEFVIIGENESDDSLLLSLRMIQYELA
WERCRQLQAEDVVVKAKVIGANKGGLVAMVEGLRGFVPFSQISSKAAAEELLEKEIP
LKFVEVDEEQTKLVLSNRKAVADSQAQLGIGSVVLGVVQSLKPYGAFIDIGGINGLLH
VSQISHDRVSDIATVLQPGDTLKVMILSHDRDRGRVSLSTKKLEPTPGDMIRNPKLVFE
KAEEMAQTFRQRIAQAEAMARADMLRFQPESGLTLSSEGILGPLGSDLPDDGVDLTV
GDIPPAVVDL.

>Gene0547670.1

MAATLGRDQYVYMAKLAEQAERYEEMVNFMEKLVTGATPSSELTVEERNLLSVAYK

NVIGSLRAAWRIVSSIEQKEESRKNEEHVALVKDYRSKVETELSSVCEGILKLLEENLIP

SAAASESKVFYLKMKGDYHRYMAEFKAGEERKAAAEDTMVAYKAAQDIAAADMA

PTHPIRLGLALNFSVFYYEILNSSDKACDMAKQAFEEAIAELDTLGEESYKDSTLIMQL

LRDNLTLWTSDMQILIFLFVVDQSKSIVMEFQFNPFRLHESRQVDQ.

>Gene0551100.1

MYRRATAGVRSASTTLTRLSSSSLASAPAASSSAPSASVINQTSGSRSFSSALRSYRVCS

ASTRWSHGGSWRSPASLRAQARVSAPVMERLERRYASMASEHTYQDILTSLPKPGGG

EYGKYYSLPALNDPRIDKLPYSVRILLESAIRNCDNYQVTKDDVEKILDWENTSTKQV

EIAFKPARVILQDFTGVPALVDLASMRDAVKNLGSDPNKINPLVPVDLVVDHSVQVDF

ARSEDAAQKNMELEFKRNKERFAFLKWGSTAFQNMLVVPPGSGIVHQVNLEYLGRV

VFNSGGFLYPDSVVGTDSHTTMIDGLGVAGWGVGGIEAEAAMLGQPMSMVLPGVV

GFKLDGKLKEGVTATDLVLTVTQILRKHGVVGKFVEFYGEGMSELSLADRATIANMS

PEYGATMGFFPVDHVTLEYLKLTGRSDETVSMIESYLRANNMFVDYNEPQQERAYTS

YLQLDLGHVEPCISGPKRPHDRVPLKDMKADWHACLDNPVGFKGFAVPKEKQGEVV

KFSYDGQPAEIKNGSVVIAAITSCTNTSNPSVMIGAALVAKKAFDLGLKVKPWVKTSL

APGSRVVEKYLDRSGLREDLNKQGFQIVGYGCTTCIGNSGDLDKSVAAAIEGTDIIPAA

VLSGNRNFEGRVHPQTRANYLASPPLVVAYALAGTVDIDFETEPLGTGKDGKNVFLR

DIWPSNEEVAKVVQYSVLPSMFKSSYETITEGNPLWNELSAPGSTLYSWDSNSTYIHEP

PYFKNMTANPPGPREVKDAYCLLNFGDSVTTDHISPAGNIQKTSPAAKFLMDRGVSQ

TDFNSYGSRRGNDEVMARGTFANIRLVNKLLKGEVGPKTVHVPTGEKLSVFDAASRY

MNAGQDTVILAGAEYGSGSSRDWAAKGPLLLGVKAVIAKSFERIHRSNLAGMGIIPLC

FKAGEDADTLGLTGHERYTVHLPTKVSDIKPGQDVTVTTDTGKSFVCTLRFDTEVEL

AYYDHGGILPYVIRSLSAK.

>Gene0553250.1

MRSLIFLAILSLLALTFPVAIASDPSPVQDFCVGVNTPSNGVFVNGKFCKDPKLATIDDF

FFTGLDRQRVATNAVGTNVTAVFADNLPGLNTLGIAFARVDYAPNGLIPPHTHPRASEF

LIVQEGSLYAGFVSSDQDGNRLFCKILNKGDLIVFPVGLIHFHVNVGRGPAVAFTAFNS

QNPGLITIAKTVFGSNPRINPNALAKAFQLDPRIVMSLQTKF.

>Gene0554940.1

MSHLVSTLHLYNIANVKRVVADLSLCNTHSQVLAFTGPSELADCLKDVNVMVIPAGV
PRKHSMTRNTVNSTVPIAAQVLTKKGVYDPKKLFGVTTLDVVRANTFVSQKKKLKHI
IVDVPVIGGHAGITILPIFSKTKPSASLTDEEFQELTASDSECWN.

>Gene0555070.1

MAAQASGLFSSAVTTAATSGVKKLHLFTTSHRPRSLSFPKTAIRAEKADSAAAAPAAA
VKEEAPVGFTPPQLDPNTPSPIFAGSTGGLLRKAQVEEFYVITWNSPKEQIFEMPTGGA
AIMREGPNLLKLARKEQCLALGTRLRSKYKINYQFYRVFPNGEVQYLHPKDGVYPEK
ANAGREGVGQNMRSIGKNVSPIEVKFTGKQSYDLYSTSFLDFVRKSRNGFLINKSDHP
QEDAYLGIQAALEWIEAKEDLKLDNSDIQATSYLETDTKFSSFTLNN.

>Gene0555280.1

MIIRSPEPEVKILVDRDPIKTSFEEWAKPGHFSRTIAKGPDTTTWIWNLHADAHDFDSH
TSDLEEISRKVFSAHFGQLSIIFLWLSGMYFHGARFSNYEAWLSDPTHIGPSAQVVWPI
VGQEILNGDVGGGFRGIQITSGFFQLWRASGITSELQLYCTAIGALVFAALMLFAGWF
HYHKAAPKLAWFQDVESMLNHHLAGLLGLGSLSWAGHQVHVSLPINQFLNAGVDP
KEIPLPHEFILNRDLLAQLYPSFAEGATPFFTLNWSKYSDFLTFRGGLDPVTGGLWLTD
TAHHHLAIAILFLIAGHMYRTNWGIGHGLKDILEAHKGPFTGQGHKGLYEILTTSWHA
QLSLNLAMLGSLTIVVAHHMYSMPPYPYLATDYATQLSLFTHHMWIGGFLIVGAAAH
AAIFMVRDYDPTNRYNDLLDRVLRHRDAIISHLNWVCIFLGFHSFGLYIHNDTMSALG
RPQDMFSDTAIQLQPVFAQWIQNTHALAPGVTAPGETASTSLTWGGGELVAVGGKVA
LLPIPLGTADFLVHHIHAFTIHVTVLILLKGVLFARSSRLIPDKANLGFRFPCDGPGRGG
TCQVSAWDHVFLGLFWMYNSISVVIFHFSWKMQSDVWGSISDQGVVTHITGGNFAQ
SSITINGWLRDFLWAQASQVIQSYGSSLSAYGLFFLGAHFVWAFSLMFLFSGRGYWQE
LIESIVWAHNKLKVAPATQPRALSIVQGRAVGVTHYLLGGIATTWAFFLARIIAVG.
