# Supplementary material for: Quantitative Succinyl-Proteome Profiling of Turnip (Brassica rapa var. rapa) in Response to Cadmium Stress
Source: Cells. 2022 Jun 17;11(12):1947. doi: 10.3390/cells11121947 (PMC9221971; doi:10.3390/cells11121947)
Supplement: Supplementary file 1 [file cells-11-01947-s001.zip › Supplementary Figures.pdf]

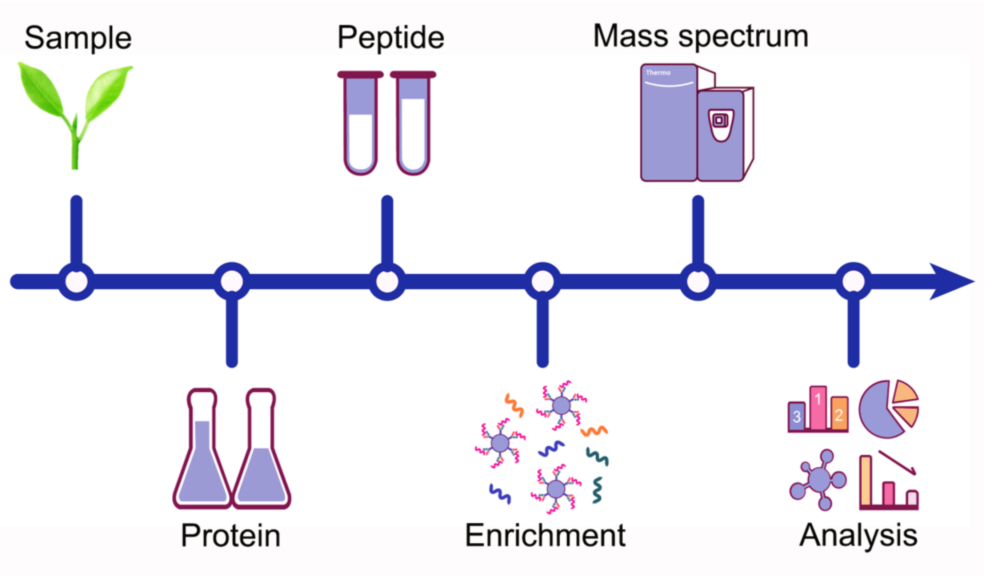

Figure S1. Workflow for global lysine succinylation identification in shoots of turnip seedlings.

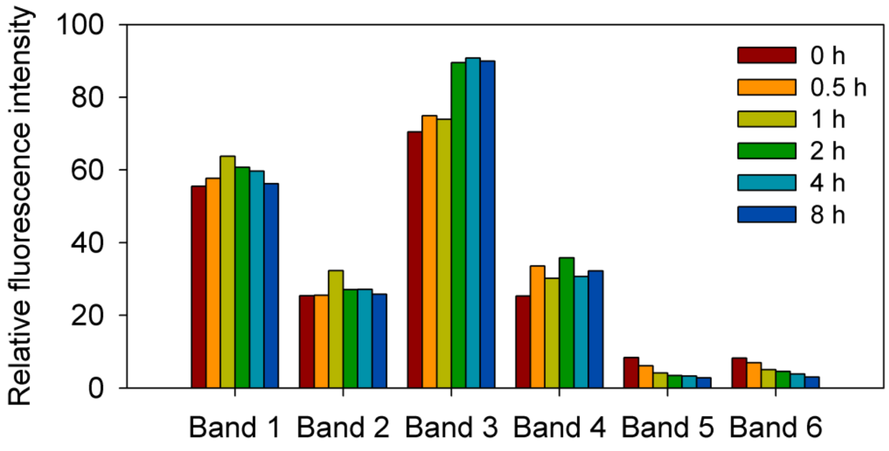

Figure S2. The relative fluorescence intensity of the main western blotting bands of acetylation modification under different Cd treatment times. The number of bands is corresponding to that in Figure 1D.

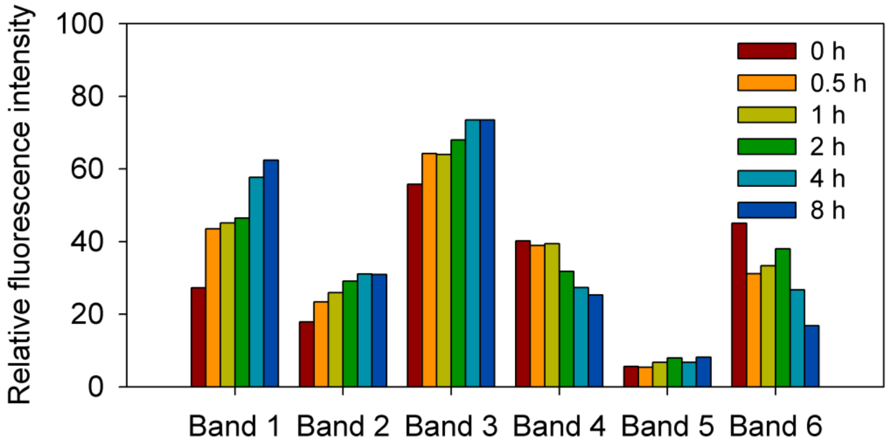

Figure S3. The relative fluorescence intensity of the main western blotting bands of succinylation modification under different Cd treatment times. The number of bands is corresponding to that in Figure1E.

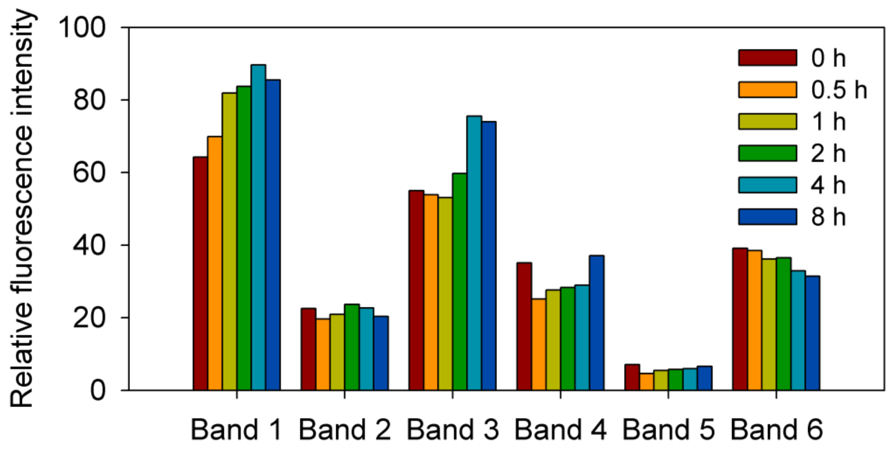

Figure S4. The relative fluorescence intensity of the main western blotting bands of crotonylation modification under different Cd treatment times. The number of bands is corresponding to that in Figure 1F.

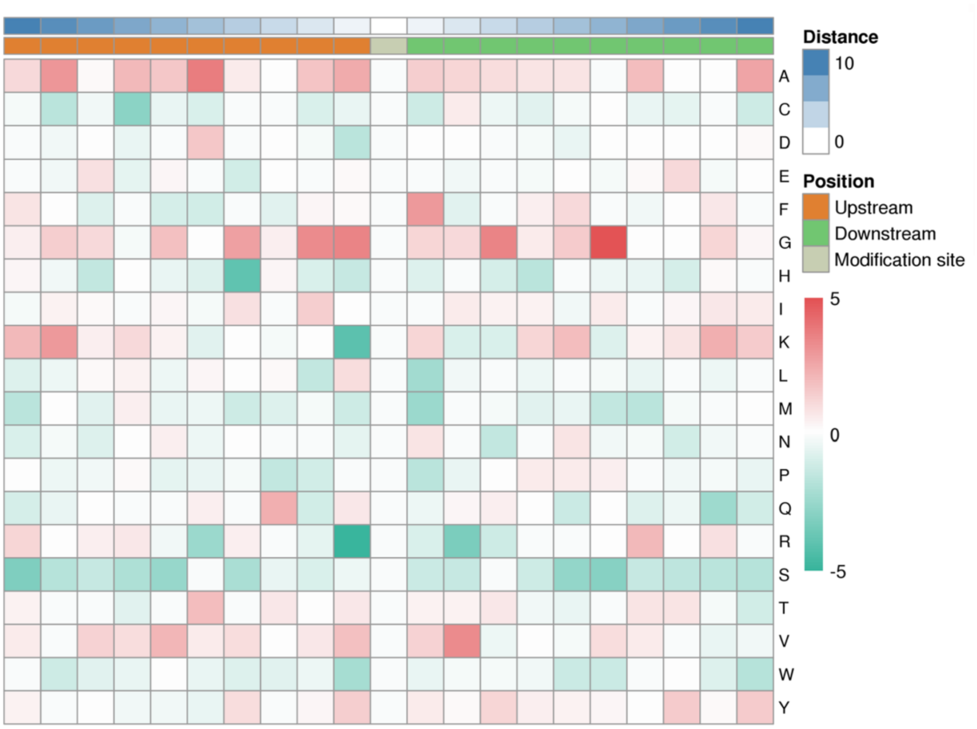

Figure S5. Heatmap analysis of the amino acid compositions around the succinylated sites. Red indicates an amino acid that is overrepresented, while green indicates an amino acid that is underrepresented.

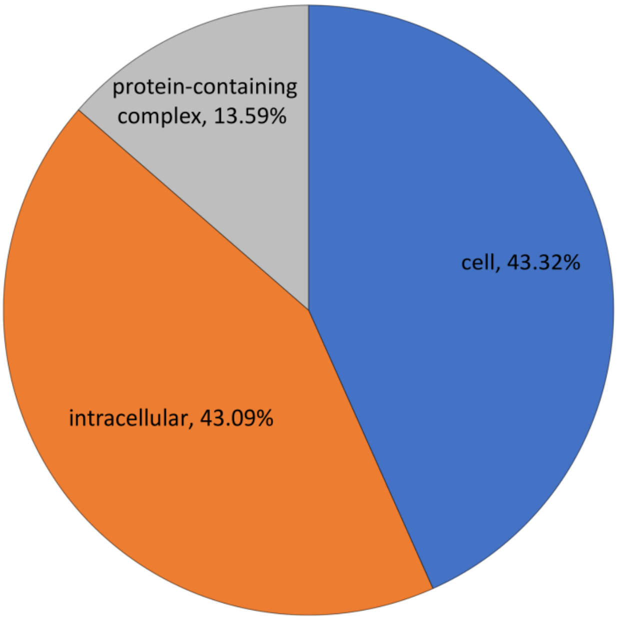

Figure S6. GO-based classification analysis of the succinylated proteins in the shoots of turnip seedlings in the categories cellular component.
